# Mapping the native interaction surfaces of PREP1 with PBX1 by cross-linking mass-spectrometry and mutagenesis

**DOI:** 10.1038/s41598-020-74032-w

**Published:** 2020-10-08

**Authors:** Chiara Bruckmann, Simone Tamburri, Valentina De Lorenzi, Nunzianna Doti, Alessandra Monti, Lisa Mathiasen, Angela Cattaneo, Menotti Ruvo, Angela Bachi, Francesco Blasi

**Affiliations:** 1 IFOM (Foundation FIRC Institute of Molecular Oncology), Via Adamello 16, 20139 Milan, Italy; 2 Institute of Biostructures and Bioimaging (IBB)-CNR, Via Mezzocannone 16, 80134 Naples, Italy; 3 Present Address: Department of Experimental Oncology, European Institute of Oncology, Via Adamello 16, 20139 Milan, Italy; 4 Present Address: Center for Nanotechnology Innovation@NEST, Istituto Italiano di Tecnologia, Piazza San Silvestro 12, 56124 Pisa, Italy; 5 Present Address: Cogentech S.R.L. Benefit Corporation IT, Via Adamello 16, 20139 Milan, Italy

**Keywords:** Biochemistry, Developmental biology, Structural biology

## Abstract

Both onco-suppressor PREP1 and the oncogene MEIS1 bind to PBX1. This interaction stabilizes the two proteins and allows their translocation into the nucleus and thus their transcriptional activity. Here, we have combined cross-linking mass-spectrometry and systematic mutagenesis to detail the binding geometry of the PBX1-PREP1 (and PBX1-MEIS1) complexes, under native in vivo conditions. The data confirm the existence of two distinct interaction sites within the PBC domain of PBX1 and unravel differences among the highly similar binding sites of MEIS1 and PREP1. The HR2 domain has a fundamental role in binding the PBC-B domain of PBX1 in both PREP1 and MEIS1. The HR1 domain of MEIS1, however, seem to play a less stringent role in PBX1 interaction with respect to that of PREP1. This difference is also reflected by the different binding affinity of the two proteins to PBX1. Although partial, this analysis provides for the first time some ideas on the tertiary structure of the complexes not available before. Moreover, the extensive mutagenic analysis of PREP1 identifies the role of individual hydrophobic HR1 and HR2 residues, both in vitro and in vivo.

## Introduction

PREP1 (Pbx-regulating protein 1, aka PKNOX1), and MEIS1 (Myeloid ecotropic insertion site 1) are transcription factors belonging to the three amino acids loop extension (TALE) homeodomain family^1^, and play essential roles during embryonic development and cancer by associating to pre-B-cell leukemia (PBX) cofactors^2,3^. PREP1 or MEIS1 dimerize with PBX1 and direct its DNA binding to specific and only partially overlapping sequences^4–8^. Unveiling the PBX-interaction surface of PREP or MEIS would allow not only to better understand their molecular function but would also represent the basis for searching inactivating therapeutic molecules.

PREP1 is essential in embryonic development^9,10^ and in the adult functions as a tumour-suppressor^11^. The tumour-suppressor role of PREP1 is associated with the maintenance of genomic stability^12^, control of DNA replication timing^13^, and protection of the nuclear envelope structure (Purushotaman D. et al., in preparation). Prep1 hypomorphic (*Prep1*^i/i^) mouse fibroblasts accumulate DNA damage, chromosomal abnormalities and display increased basal and genotoxicity-induced apoptosis^12^. Indeed, down-regulation of PREP1 induces DNA damage and consents oncogenes like Ras or Meis1 to evade senescence and transform^12^. *Prep1*^i/i^ mice develop spontaneous tumours also at the heterozygous state and Prep1 haplo-insufficient mice accelerate the development of oncogenes-dependent tumours^9,11,14^. Human tumours are low in PREP1^11,15^, while PREP1 overexpression slows-down the growth of tumour cells in vitro and in vivo^4^. Because of these properties PREP1 is considered a tumour suppressor. In particular, overexpression of PREP1 inhibits the growth of MEIS1-dependent tumour cells, competing in vivo for the MEIS1 DNA-binding sites^4,5^. All known functions of PREP1 depend on the association with PBX.

MEIS1 has a crucial role in embryonic hematopoiesis^16,17^, and is involved in leukemogenesis in children and adults. When overexpressed, MEIS1 is able to transform tumour suppressor-deficient mouse cells in the absence of other oncogenes^4^ and to accelerate the occurrence of HoxA9-dependent Acute Myeloid Leukemia (AML)^18–20^. In human, the overexpression of MEIS1 is critical in Mixed Lineage Leukemia (MLL) caused by the fusion of the *MLL* to several other genes^19^. Thus MEIS1 inhibition represents a potentially good therapeutic target for MLL treatment^20–22^. MEIS1 overexpression has been also implicated in promotion of cell proliferation, resistance to chemotherapy of leukemic cells^23^ and in skin cancer^24^. In ovarian cancer^25^ and in neuroblastomas^26^ MEIS1 is appreciably overexpressed compared to other cancer types.

Both the tumorigenic activity of MEIS1 and the anti-tumorigenic activity of PREP1 require the interaction with the cofactor PBX^4^, which thus acts as an oncogene or as a tumour-suppressor, depending on its partner, MEIS1 or PREP1. In fact, PREP1 and MEIS1 compete molecularly and functionally^4,5^. PREP1 and MEIS1 bind PBX1 in the cytoplasm, therefore in the absence of DNA; this interaction allows the nuclear localization of the complexes^27^.

Structural knowledge of target transcription factors is potentially a tool for drug design, but in TALE proteins this is currently limited to the crystallographic and NMR structures of the HOX-PBX1 DNA-binding motif (the homeodomain) and short flanking regions^28,29^, to the NMR studies of the PREP1 homeodomain^30^ and the crystal structure of MEIS1 homeodomain^31^. No crystallographic information is available for the PREP-PBX or MEIS-PBX interaction site. Therefore, we have tried to better define the minimal PREP1-PBX1 and MEIS1-PBX1 interaction interface using biochemical, cellular and genetic techniques.

N-terminal to the homeodomain of MEIS1 and PREP1, is present a highly conserved MEINOX region^32^ which is divided into two subdomains, separated by a flexible linker. The MEINOX domains of MEIS1,2,3 and of PREP1,2 are split into two motifs called homology region 1 (HR1, aka MEIS-A) and homology region 2 (HR2, aka MEIS-B), whose secondary structure predictions suggest a α-helical structure^1^. Besides the homeodomain and HR1/HR2 domains, PREP1 and MEIS1 proteins do not share high sequence similarity^33^. However, in HR1 and HR2, both PREP and MEIS present very highly conserved heptad leucine/isoleucine-rich motifs^2,34^, previously described as leucine zippers^20,35^. The almost-identity makes it difficult to discriminate between PREP1 vs. MEIS1 binding to PBX, but on the other side it allows to transfer to MEIS1 the information obtained from PREP1.

The PBX proteins contain at their amino terminus two motifs, PBC-A and PBC-B, which together are known as the PBC domain. Deletion of the HR1-HR2 domains in PREP1 indicates that they are required for the dimerization to the PBC domain of PBX^36,37^ as it abolishes the formation of the PREP1-PBX1 complex and prevents nuclear localization^4^. Mutating L63 and L67 (HR1 domain of PREP1) into alanine decreases the binding to PBX1^36^. In MEIS1, band shift experiments have shown that deletions within the HR1 region (ΔL71-L96) impair dimerization with PBX1^38^. Deletion of 3 or 4 residues in the heptad repeats of HR1 or HR2 domains of MEIS1 (HR1ΔLFPLL or HR2ΔLLEL) has been reported to be insufficient to disrupt the interaction with PBX, while their combination (HR1ΔLFPLL/HR2ΔLLEL) or deletion of more residues within the heptad repeat of the HR2 domain (HR2ΔLRF/ΔLLEL or HR2ΔIQVL/ΔLLEL) completely destroys the interaction with PBX and prevents leukemogenesis, without altering MEIS1 expression^39^. In addition, mutations of 5 hydrophobic residues into alanine in the HR2 151-VLRFHLLELE-160 stretch of MEIS1^20^ abolish the co-immunoprecipitation with PBX.

In PBX proteins the highly conserved 75-residues-long PBC-A and 88-residues long PBC-B are identified as PREP or MEIS binding regions. However, so far only the direct involvement in heterodimerization of the PBC-A has been demonstrated^2^.

As PREP1 and MEIS1 heterodimerize with the same surface of PBX, association with PBX is mutually exclusive. Therefore, PREP1 competes with MEIS in the oncogenic function^4^. In this paper we have reanalysed the PREP1-PBX1 interaction using more detailed and alternative approaches and better defined the specific interaction surface. In addition, we have performed experiments on MEIS1 that have allowed us to identify differences in the interaction mechanisms between PREP1 and MEIS1.

## Results

### Identification of the intra- and inter-molecular interacting sites in the PBX1-PREP1 and PBX1-MEIS1 complexes

Cross-linking coupled with mass-spectrometry (XLMS)^40–46^ enables visualization of the interacting regions by bridging two proteins with a cross-linker and creating hybrid peptides that are identified by mass-spectrometry following protein dissection by enzymatic proteolysis. The chemical cross-linker covalently connects two functional groups (i.e. lysines) in close proximity and exposed on the protein’s outer surface. Since distant or buried residues are not able to react with the cross-linker, XLMS allows to map protein complex interfaces.

In these experiments we used PREP1 and PBX1 constructs lacking the C-terminal region not involved in heterodimerization^3,4^ and very prone to degradation in solution^47^. On the contrary, we used the full-length MEIS1 for studying the MEIS1-PBX1 complex. As cross-linker, we used the commercial BS^3^, a homobifunctional cross-linker containing a 11.3 Å spacer, able to link two lysine residues (see “Materials and methods” for details; for the workflow of the experiment see Supplementary Information Figure S1A). Purified PREP1-PBX1 and MEIS1-PBX1 complexes were cross-linked separately with BS^3^ (Supplementary Information Figure S1B) prior to in-gel double digestion with Glu-C and trypsin. Cross-linked peptides were analysed by nLC-ESI–MS/MS, identified using the pLink 2 software and manually curated. The results derive from two biological replicates for each protein complex. The same procedure was applied to the individual proteins to identify potential intra-molecular cross-linking. All the spectra are deposited at the PeptideAtlas repository (PASS01497).

In the case of PREP1-PBX1, the identified cross-linked peptides are listed in Tables 1, 2, and 3 while the full-list with the statistical significance is available in Supplementary Information Tables S4–S7. The XLMS analysis revealed 12 unique intra-PBX1, 3 intra-PREP1 and 12 inter-protein cross-links, in addition to 6 loop-linked sites (2 from PBX1 and 4 from PREP1). With the MEIS1-PBX1 dimer a total of 24 non-redundant cross-linked sites was obtained: 8 inter-, 11 intra-molecular cross-linked sites and 5 loop-linked sites (see Tables 4, 5 and 6 for the summary of peptides identified, and Supplementary Information Tables S8–S11 for the full list of redundant peptides and their statistical significance).

**Table 1:** Summary of PBX1 and PREP1 intermolecular peptides. The number under brackets are the lysine residues cross-linked, in the first column refers to the residue in the full-length protein, in the second column to the residue in the peptide. The peptides here reported correspond to peptide identified in two biological replicates of the XLMS experiment.

Cross-link	Peptides identified
PBX1(87)-PREP1(55)	KTVLSIR(1)-SQTPMDVDKQAIYR(9)
PBX1(308)-PREP1(134)	ANIYAAK(7)-LEKVNE(3)
PBX1(308)-PREP1(268)	ANIYAAK(7)-GVLPKHATNVMR(5)
PBX1(87)-PREP1(73)	KTVLSIR(1)-KCEQSTQGSE(1)
PBX1(308)-PREP1(140)	ANIYAAK(7)-LCKDFCSR(3)
PBX1(153)-PREP1(268)	AKLSQIR(2)-GVLPKHATNVMR(5)
PBX1(65)-PREP1(134)	KHALNCHR(1)-LEKVNE(3)
PBX1(87)-PREP1(25)	KTVLSIR(1)-LKTEQD(2)
PBX1(195)-PREP1(268)	TRPISPKEIER(7)-GVLPKHATNVMR(5)
PBX1(153)-PREP1(151)	AKLSQIR(2)-YIACLKTK(6)
PBX1(65)-PREP1(25)	KHALNCHR(1)-LKTEQDPNCSEPDAE(2)
PBX1(153)-PREP1(153)	AKLSQIR(2)-TKMNSE(2)

**Table 2:** Summary of PBX1 and PREP1 intramolecular peptides.

Cross-link	Peptides identified
PBX1(87)-PBX1(195)	KTVLSIR(1)-TRPISPKEIER(7)
PBX1(242)-PBX1(308)	NFNKQATE(4)-EANIYAAK(8)
PBX1(65)-PBX1(195)	KHALNCHR(1)-TRPISPKEIER(7)
PBX1(195)-PBX1(308)	TRPISPKEIER(7)-ANIYAAK(7)
PBX1(153)-PBX1(308)	AKLSQIR(2)-ANIYAAK(7)
PBX1(153)-PBX1(242)	AKLSQIR(2)-NFNKQATE(4)
PBX1(87)-PBX1(308)	KTVLSIR(1)-ANIYAAK(7)
PBX1(87)-PBX1(153)	KTVLSIR(1)-AKLSQIR(2)
PBX1(65)-PBX1(308)	KHALNCHR(1)-ANIYAAK(7)
PBX1(65)-PBX1(74)	KHALNCHR(1)-MKPALFNVLCE(2)
PBX1(65)-PBX1(87)	KHALNCHR(1)-KTVLSIR(1)
PBX1(74)-PBX1(195)	MKPALFNVLCE(2)-TRPISPKE(7)
PREP1(134)-PREP1(140)	LEKVNE(3)-LCKDFCSR(3)
PREP1(140)-PREP1(151)	LCKDFCSR(3)-YIACLKTK(6)
PREP1(134)-PREP1(268)	LEKVNE(3)-GVLPKHATNVMR(5)

**Table 3:** Summary of PBX1 and PREP1 looped peptides.

Cross-link	Peptides identified
PREP1 (260)(262)	LSILHQDDGSSKNKR(12)(14)
PREP1 (102)(105)	KEGKPFFCEDPE(1)(4)
PREP1 (334)(335)	KKTAQNRPVQR(1)(2)
PREP1 (331)(333)	SSCSETPKTKK(8)(10)
PBX1 (292)(293)	YKKNIGK(2)(3)
PBX1 (85)(87)	IKEKTVLSIR(2)(4)

**Table 4:** Summary of PBX1 and MEIS1 intermolecular peptides.

Cross-link	Peptides identified
MEIS1 (195)-PBX1 (153)	GGSKSDSEDITR(4)-AKLSQIR(2)
MEIS1 (195)-PBX1 (242)	EGGSKSDSEDITR(5)-NFNKQATE(4)
MEIS1 (178)-PBX1 (153)	YISCLKGK(6)-AKLSQIR(2)
MEIS1 (161)-PBX1 (65)	LEKVHE(3)-KHALNCHR(1)
MEIS1 (195)-PBX1 (308)	EGGSKSDSEDITR(5)-ANIYAAK(7)
MEIS1 (195)-PBX1 (297)	EGGSKSDSEDITR(5)-NIGKFQEE(4)
MEIS1 (195)-PBX1 (87)	EGGSKSDSEDITR(5)-KTVLSIR(1)
MEIS1 (161)-PBX1 (308)	LEKVHE(3)-ANIYAAK(7)

**Table 5:** Summary of PBX1 and MEIS1 intramolecular peptides.

Cross-link	Peptides identified
PBX1 (87)-PBX1(195)	KTVLSIR(1)-TRPISPKEIER(7)
PBX1 (153)-PBX1 (242)	AKLSQIR(2)-NFNKQATE(4)
PBX1 (153)-PBX1 (308)	AKLSQIR(2)-ANIYAAK(7)
PBX1 (297)-PBX1(308)	NIGKFQEE(4)-ANIYAAK(7)
PBX1 (242)-PBX1(308)	GGSKSDSEDITR(4)-ANIYAAK(7)
PBX1 (195)-PBX1 (308)	TRPISPKE(7)-ANIYAAK(7)
PBX1 (87)-PBX1(242)	KTVLSIR(1)-NFNKQATE(4)
PBX1(87)-PBX1 (153)	KTVLSIR(1)-AKLSQIR(2)
MEIS1 (178)-MEIS1 (195)	YISCLKGK(6)-GGSKSDSEDITR(4)
MEIS1 (126)-MEIS1 (132)	DIAVFAKQIR(7)-AEKPLFSSNPE(3)
MEIS1 (161)-MEIS1 (195)	LEKVHE(3)-EGGSKSDSEDITR(5)

**Table 6:** Summary of PBX1 and MEIS1 looped peptides.

Cross-link	Peptides identified
PBX1 (85)(87)	IKEKTVLSIR(2)(4)
PBX1 (292)(293)	YKKNIGKFQEE(2)(3)
PBX1(293)(297)	YKKNIGKFQEE(3)(7)
MEIS1 (270)(271)	PDKDKKR(5)(6)
MEIS1(305)(306)	QKKQLAQD(2)(3)

**Figure 1:**
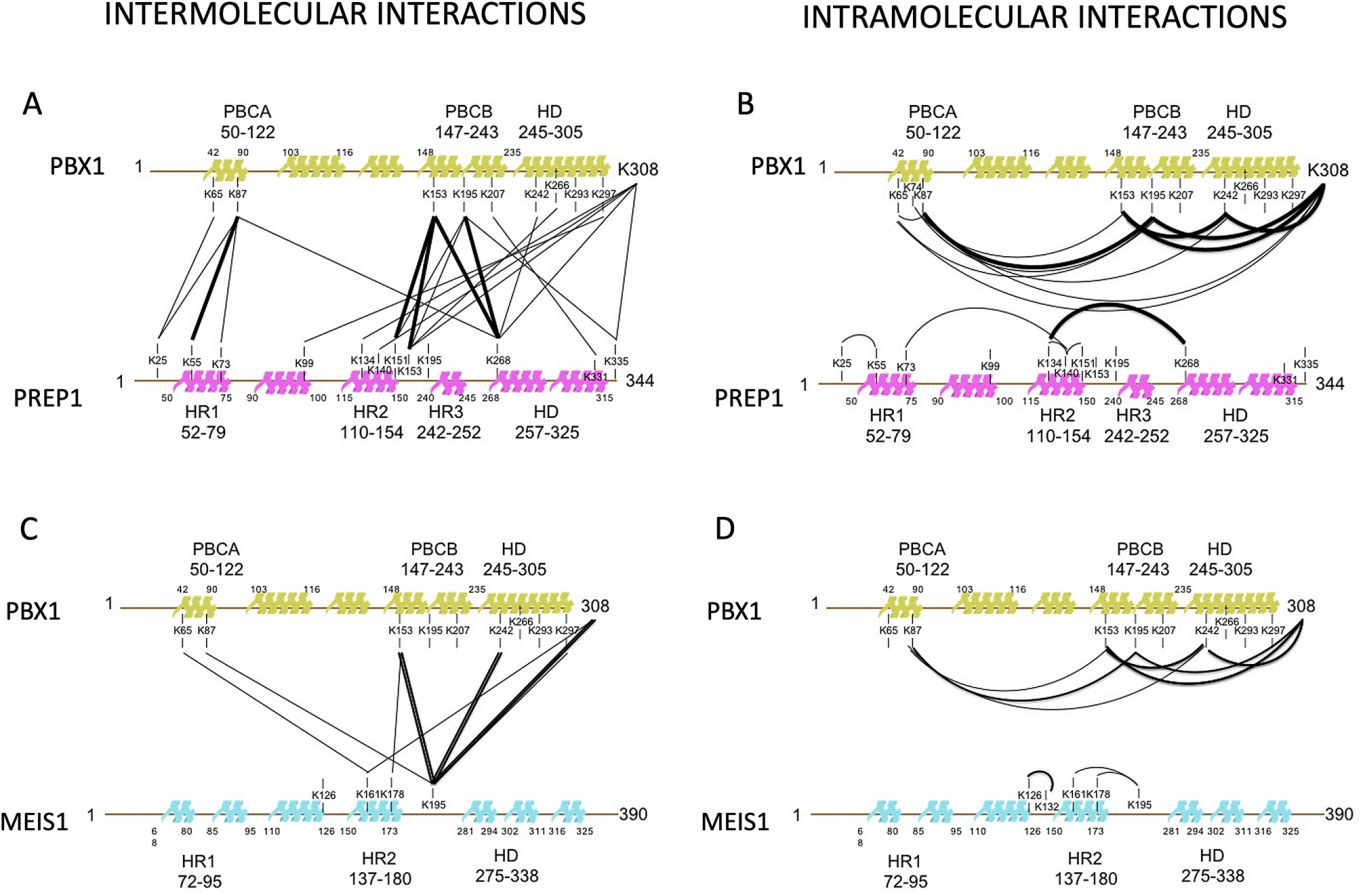
Two-dimensional mapping of the intra- and inter-protein cross-links identified by XLMS in the PREP1-PBX1 and MEIS1-PBX1 complexes. Alpha-helical domain are represented by colored cartoon on the linearized sequences and labelled according to amino acid position (N- to C-terminal). Residues (lysine) involved in cross-links are indicated. Lines in bold designate peptides found in both biological replicates of the experiment. **Panel A**: Inter cross-linking in the PREP1-PBX1 complex. **Panel B**: Intra cross-linking in the PREP1-PBX1 complex. **Panel C**: Inter cross-linking in the MEIS1-PBX1 complex. **Panel D**: Intra cross-linking in the MEIS1-PBX1 complex.

To visualize the regions of interaction, since no high-resolution structural data on full-length PREP1, MEIS1 or PBX1 are available, we mapped the cross-linked peptides on linearized molecules (Fig. 1), also evidencing the reactive lysines (Supplementary Information Figures S2A, S2B, and S2C). In Fig. 1, thick lines represent cross-links identified in both experimental replicates while thinner ones refer to those detected in single experiments, filtered with the stringent criteria defined in the Methods section. The XLMS analysis identified in PREP1 the following cross-linked lysines: K55(HR1), K151 and K153(HR2) and K268 (homeodomain); those identified in PBX1 were K87(PBC-A) and K153 and K195 in PBC-B. These cross-linked residues connect the HR1 and HR2 domains of PREP1 to PBC-A and PBC-B of PBX1, respectively. In addition, K87 on PBX1 was also found cross-linked with K268 of PREP1, suggesting a special proximity of PBC-A to the homeodomain of PREP1. Likewise, the homeodomain of PBX1 was found cross-linked with the homeodomain [(K242(PBX1)-K268(PREP1)] and with the HR2 domain (K266(PBX1)-K153(PREP1) of PREP1. The C-terminal residues K297(PBX1) and K308(PBX1) were found cross-linked with several PREP1 lysines mainly located in the central part of the protein (K99, K134, K140, K153, K268 and K335), possibly reflecting a flexibility of this region. Altogether the data suggest that the main interacting regions in the PREP1-PBX1 complex are included in the HR1-HR2 domains of PREP1 and in the PBC-A-PBC-B of PBX1.

In MEIS1, the HR2 domain (K161, K178 and K195) was cross-linked with the PBC-A (K65 and K87), the PBC-B (K153), the homeodomain (K242) and with the C-terminal region (K297 and K308) of PBX1. No MEIS1 residue in the HR1 cross-linked with PBX1, suggesting that the HR1 domain of MEIS1 is not or less involved in the heterodimerization. We thereby conclude that in the MEIS1-PBX1 complex, the HR2 of MEIS1 and the PBC-B of PBX1 are the domains mostly involved in the formation of the heterodimer. It must be noticed that K195 in the PBC-B of PBX1 was not found in any inter cross-linked peptide with MEIS1, while it was very reactive in the complex with PREP1, in particular with the PREP1 homeodomain (K268). The lack of reactivity of the homeodomain of MEIS1 might be due to an inherent limit of the technology. Indeed, our XLMS analysis revealed that K270, K271, K305 and K306 of MEIS1 were present in the looped cross-linked peptides (see Supplementary Information Figure S2B and Table 6), and therefore must be exposed and reactive. On the other hand, the homeodomain region of MEIS1 is very rich in K, R, E, and D (see Supplementary Information Figure S2B) which would lead to an extreme fragmentation of peptides upon digestion with trypsin and Glu-C. For these reasons, any intra or inter-protein interaction occurring C-terminally of the HR2 (K195) of MEIS1 might be invisible by XLMS. Therefore, we have used an alternative approach (TR-FIA analysis with PBX1 deletion mutants, see below) to gain information on the importance of 197–207 stretch of PBX1 in the interaction with MEIS1 (see below).

Assuming that the proteins under study are in a heterodimeric form, intramolecular cross-links identified by XLMS are also useful to examine the entire protein folding and to further validate the intermolecular connections. In our experiments, we find that PBX1 retains the same overall folding by binding to PREP1 and to MEIS1. In fact, in both cases we detect the same intramolecular peptides (see Tables 2 and 5 and Supplementary Information Tables S5, S7, S9, and S11). Also, a visual inspection of the PBX1 cross-linked peptides position suggests that PBC-A is localized near PBC-B and the homeodomain. To make the results of the experiments clearer to the reader, we have designed the interacting regions in a cartoon form (Supplementary Information Figures S1C and S1D).

### Potential PBX1-interaction interfaces of PREP1 and MEIS1

Given the previous literature data on the PREP1-PBX1 and MEIS1-PBX1 interactions^20,35,36,38,39,47^, it is clear that PBX1-PREP1 and PBX1-MEIS1 complexes are held together by hydrophobic interactions through leucines and isoleucines located in their N-terminal moiety in regions called HR1 and HR2. These regions are almost identical in PREP1 and MEIS1 (Supplementary Information Figures S2A and S2B) (20/25 residues in HR1 and 18/22 in HR2). We noticed in the PREP subfamily a third conserved leucine-rich region upstream of the homeodomain that we called HR3. This region, absent in the MEIS subfamily, lacks lysine residues in PREP1 and therefore is not found among the cross-linked hybrid peptides (Table 1). However, it is close to K268 which was found cross-linked to PBC-B (Fig. 1A and Table 1). Therefore, its involvement in the interaction with PBX1 cannot be excluded by considering only the cross-linking results.

The secondary structure of PBX1 predicts the PBC-A and PBC-B to be mainly helical; three leucine/isoleucine rich repeats highly conserved among PBX isoforms are present in the PBC-A and one in the PBC-B (Supplementary Information Figure S2C).

### Identification of residues in the hydrophobic α-helical interphases of the HR1 and HR2 domains of PREP1 that are essential for dimerization with PBX1.

**Figure 2:**
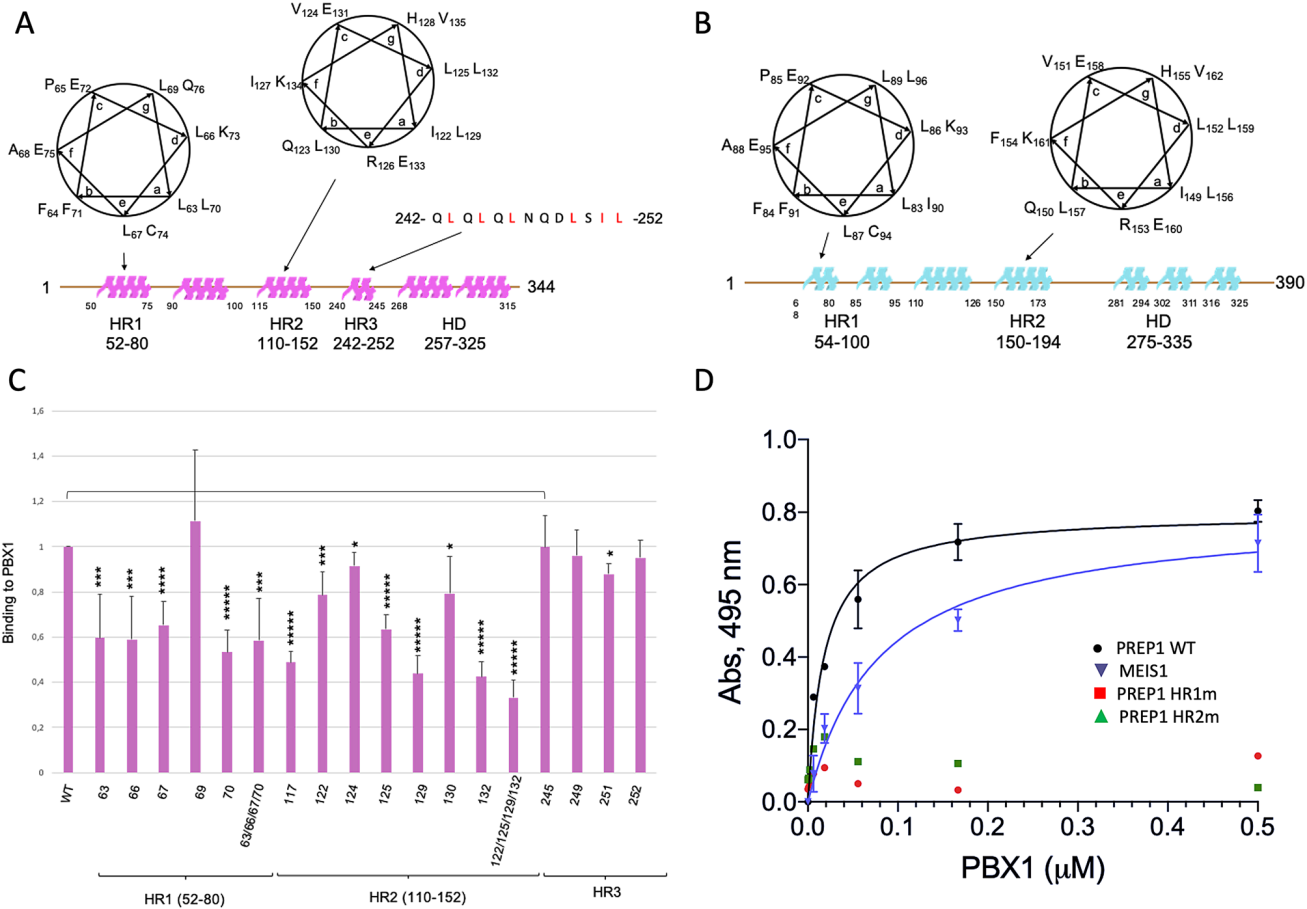
Characterization of PREP1 leucine and isoleucine mutants. **Panel A**: Mapping of the heptad repeats within HR1, HR2 and HR3 regions of PREP1. The areas in magenta represent predicted alpha-helical regions within PREP1**.** The heptad repeats of HR1 and HR2 domains are represented as alpha-helical turns above the linear sequence of the protein, highlighting the hydrophobic residues pointing toward the same face of the helix. The position and sequence of the hydrophobic “HR3” stretch are indicated. **Panel B**: Mapping of the heptad repeats within HR1 and HR2 of MEIS1. Predicted alpha-helical regions are represented as light-blue areas above the linear sequence. **Panel C**: Binding of recombinant wild-type and mutants PREP1 to recombinant wild-type PBX1 measured by Time-resolved fluorescence immunoassay (TR-FIA). Single mutations analysed in the assay are indicated; HR1m is the quadruple L63A/L66A/L67A/L70A mutant; HR2m is the quadruple I122A/L125A/L129A/L132A mutant. HR1, HR2 and “HR3” domains are mapped below the bars for clarity. The Y axis reports the binding to PBX1 of the PREP1 mutants, normalized to wild-type PREP1 taken as 1.0. The results represent the average of four independent assays, + /− the standard deviation. T-TEST was performed as described in the Methods section. **Panel D** ELISA determination of the affinity between N-terminal GST-fused wild-type PREP1 (black line), GST-fused wild-type MEIS1 (blue line), GST-fused HR1m (L63A/L66A/L67A/L70A, red squares) and HR2m (I122A/L125A/L129A/L132A, green triangles) PREP1 mutants. Excess proteins were added to plates coated with increasing concentrations of wild-type PBX1. The values on the Y axis are the signals obtained with the individual proteins subtracted of the GST used as control. Results represent the average of three independent assays + /− the standard deviation.

HR1 and HR2 in the PREP subfamily contain each a predicted helix-turn-helix motif, with hydrophobic stretches rich of leucines and isoleucines (Fig. 2A). The leucines and isoleucines heptad repeats are 80% conserved in the MEIS subfamily. Secondary structure predictions suggest the occurrence of α-helical conformations within the HR1 and HR2 regions of PREP1 and MEIS1, represented in cartoon form in Figs. 2A and 2B. In PREP1, positions “a” of the first heptad repeat of HR1 are occupied by L63 and L70 residues, and positions “d” by L66 and K73. The whole amphipathic helix counts additional hydrophobic residues (L67, L69, F71 and F64) surrounding positions “a” and “d”. We have therefore systematically mutated each of the hydrophobic residues within the heptad repeats substituting them with alanine. Mutants were GST-affinity purified and their binding ability was compared with the wild-type protein in an in vitro binding immunoassay using time-resolved fluorescence (TR-FIA). Figure 2C represents the average + /− SD of four independent experiments.

In the HR1 domain of PREP1, single mutation L69A, in position “g” of the helix, did not reduce significantly the binding to PBX1, while point mutants L63A, L66A, L67A and L70A each reduced significantly the binding to PBX1 to 50–60% (Fig. 2C).

In HR2, positions “a” and “d” within the L117-L132 heptad, are occupied by the hydrophobic residues I122 and L129 in position “a” and L125 and L132 in position “d”. We thus mutated these and other hydrophobic residues in positions “b” and “c” (L117A, V124A and L130A) into alanine. Among all the mutants, L129A and L132A which both sit in the second turn of the helix, most affected PBX1 binding; in particular, L132A reduced the binding to 40%.

We also mutated four hydrophobic residues within the HR3 domain, but none of them affected significantly the binding of PREP1 to PBX1 (Fig. 2C).

To evaluate the effect of mutating the entire set of residues in HR1 and in HR2 that reside on the same side of the α-helix, we generated the quadruple mutants HR1m and HR2m bearing the L63A/L66A/L67A/L70A and I122A/L125A/L129A/L132A mutations, respectively. As shown in Fig. 2C, HR1m bound PBX1 with the same efficiency as the single mutants, while binding of HR2m was further reduced to 30%. Overall, the data indicate that both HR domains of PREP1 are strongly involved in the recognition of PBX1. However, it appears that residues I122, L125, L129 and L132 of HR2, likely arrange to form a large and continuous hydrophobic patch that covers two helix turns, playing a relevant role for the interaction.

On the basis of these observations, we next measured the affinity for PBX1 of recombinant wild-type PREP1 and mutants HR1m and HR2m. For comparison, also the affinity of wild-type MEIS1 was determined under the same experimental conditions. Affinity was measured by dose-dependent ELISAs, by coating the different proteins on plate wells and probing the interaction with soluble PBX1. As shown in Fig. 2D, soluble PBX1 bound both PREP1 and MEIS1 although with different affinities: the K_D_ of PBX1 for PREP1 was estimated to be 18 ± 4 nM, the K_D_ of MEIS1 was almost fivefold higher (80 ± 17 nM), in agreement with the results of XLMS that indicate two major sites of interaction for PREP1 and only one for MEIS1. Importantly, in line with the mutagenesis data, PREP1 HR1m and HR2m mutants displayed no measurable binding for PBX1 under the experimental conditions used here.

We used CD spectroscopy on the wild-type protein and on the HR1m and HR2m mutants to test whether the mutations had grossly modified the global protein fold. Differences were observed in the CD spectra of HR1m and HR2m compared to the wild-type protein (Supplementary Information Figure S3D); however, the spectra were changed only in terms of intensity and not in terms of intrinsic CD signals, indicating that the secondary structure of the proteins was globally maintained. In fact, the CD spectrum of PREP1 wild-type showed the typical signatures of α-helix-rich structures with two minima centred at ~ 208 and ~ 222 nm, and a maximum at ~ 195 nm, in agreement with data reported in the literature^48^. Similarly, the conformational analysis of HR1m and HR2m quadruple mutants of PREP1 indicated a persistence of the α-helical structures with minima at ~ 208 and ~ 222 nm, and a positive peak at ~ 195 nm. Data thus show that the conformation of the three proteins, was globally preserved also in the presence of four mutations, in agreement with previous studies showing that PREP1 is endowed with a remarkable structural stability^48^ and possesses a high tolerance to conformational changes.

In conclusion, the CD data allow to conclude that the deficiency in PBX-interaction of PREP1 mutants is due to the substitution of the hydrophobic residues and not to an overall unfolding effect.

### The contribution of the PBC domain of PBX1 in the interaction with PREP1

**Figure 3:**
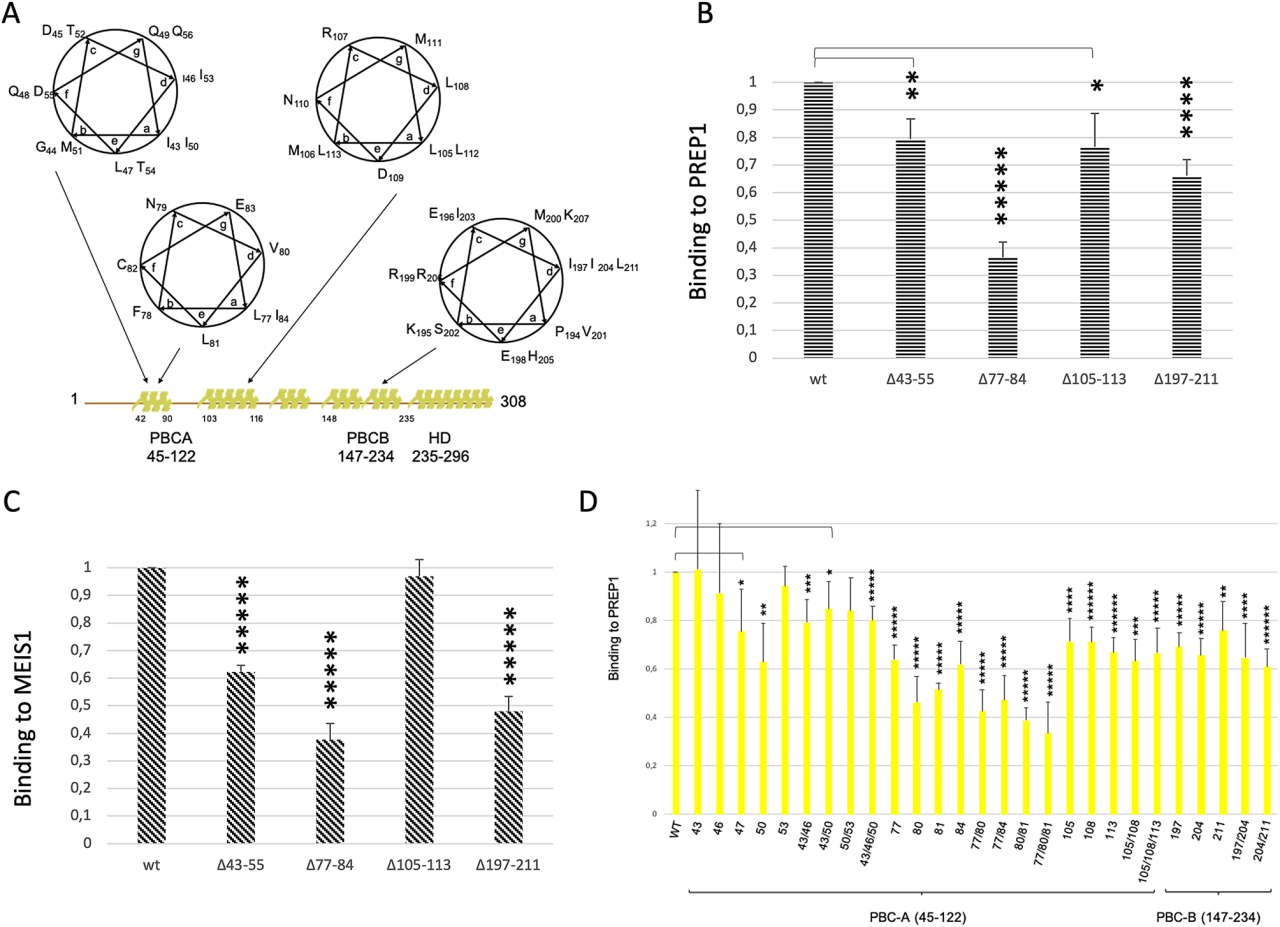
Characterization of PBX1 mutants. **Panel A**: Mapping of the heptad repeats of PBX1within PBC-A and PBC-B. Predicted alpha-helical regions are represented as yellow areas above the linear sequence. Boundaries of PBC-A, PBC-B and homeodomain are defined below the linearized sequence. **Panel B**: Time-resolved fluorescence immunoassay (TR-FIA)-measured binding of wild-type and mutant PBX1 to wild-type PREP1. The Y axis reports the binding to PREP1 of the PBX1 deletion mutants, normalized for the binding of wild-type PBX1 taken as 1. The results represent the average of three independent assays, + /− the standard deviation. The extent of deletions is indicated below the ordinates bar. **Panel C**: TR-FIA-measured binding of wild-type and mutant PBX1 to wild-type MEIS1. The Y axis reports the binding to MEIS1 of the PBX1 deletion mutants, normalized for the binding of wild-type PBX1 taken as 1. The results represent the average of three independent assays, + /− standard deviation. **Panel D**: TR-FIA-measured binding of wild-type and point mutants of PBX1 to wild-type PREP1. Single and multiple mutations analysed are indicated below the bars. PBC-A and PBC-B are mapped below the ordinates bar. The Y axis reports the binding to PREP1 of the PBX1 mutants, normalized for the binding of wild-type PBX1 taken as 1. The results represent the average of four independent assays, + /− the standard deviation. T-TEST was performed as described in the Methods section.

It has been previously reported that partial deletions within PBC-A of PBX1 affect the binding to PREP or MEIS^2,49^. PBX1 contains four leucine/isoleucine rich-stretches arranged in heptad repeats, three in PBC-A and one in PBC-B (Fig. 3A). We have generated deletion mutants lacking each entire hydrophobic stretch together with a number of single and double substitution mutants, and tested them for binding to PREP1 by TR-FIA. The region I43-D55 contains two heptad repeats, with the “a” and “d” positions of the amphipatic helix occupied by isoleucines (I43, I53) that create a hydrophobic interface with L46, I50. Positions “c”, “f” and “g” are occupied by polar residues (D45, Q48, Q49, T52, D55, and Q56), while in position “e” a leucine residue (L47) is present, highly conserved within the PBX family (Supplementary Information Figure S2C). Binding of PBX1Δ43-55 to PREP1 and MEIS1 was decreased to 80% and 60%, of wild-type, respectively (Fig. 3B and 3C). The second stretch (L77-L90) has a single heptad repeat, with L77 in position “a” and V80 in position “d” or, alternatively, with L81 in position “a” and I84 in position “d”. In fact, the whole stretch is rich in hydrophobic residues (L77, F78, V80, L81, I84, V89, L90; see Supplementary Information Figure S2C) and might constitute an apolar interface for the interaction with PREP1/MEIS1. Indeed, deletion Δ77−84 reduced the binding to both PREP1 and MEIS1 to 35%. In the third stretch, the leucine residues in this region (L105, L108, L112) occupied all the “a” or “d” positions of the helix (see Fig. 3A). Δ105-113 deletion mutant was able to decrease the binding to PREP1 to 75% but had no effect on MEIS1. This is consistent with the XLMS data, where we could not observe any PBX1 peptide from the 105–113 stretch cross-linking PREP1 or MEIS1. In PBC-B, a single hydrophobic heptad repeat is present with V201 in position “a” and I197 and I204 in position “d”. The PBX1Δ197-204 deletion (Fig. 3B) reduced to about 65% the binding of PREP1 and to 45% that of MEIS1.

Figure 3D shows the effect of single substitutions in PBX1. In the 43–53 stretch, substitution of single (and some double) hydrophobic isoleucine residues gave variable results but all mutants bound in the range of the wild-type. Thus, hydrophobic residues within this stretch appear to have a partial, if any, role in the interaction with PREP1, in agreement with the results on the deletions (Fig. 3B). In the second stretch, single mutations of most hydrophobic residues (V80, L81 and I84) suppressed significantly the binding to PREP1 to 40–60%; in double or triple mutants (L77A/V80A, L77A/I84A, V80A/L81A, L77A/V80A/L81A) the effect was slightly increased, reducing the binding to PREP1 to 30–40%. Because of the stronger effects of V80A on the binding to PREP1, we hypothesize that the heptad repeat may consist of L77 and I84 in position “a” and V80 in position “d”. In the 105–113 deletion, single alanine substitution of the leucine residues in this region (L105, L108, L112) had only a minor effect even in the double and triple-substituted mutants (L105A/L108A and L105A/L108A/L112A) (Fig. 3D). These data overall confirm the importance of PBC-A for the binding to PREP1 and/or MEIS1 and highlight the major role played by its second hydrophobic repeat (77–84).

In PBC-B, substitution of the single isoleucine residues (I197A, I204A or I211A) reduced the binding to PREP1 only moderately (to about 65%). Substitution of both 204 and 211 residues had a stronger effect (reduction to about 50%).

In conclusion, the interaction of PBX1 with PREP1/MEIS1 occurs not only through PBC-A (43–55 and 77–84 stretches), but also through PBC-B (residues 197–204). However, we can hypothesize that the most important interaction of the PBC-B region 197–207 occurs with the homeodomain of PREP1 and MEIS1 that in MEIS1 is invisible by XLMS due to the intrinsic limit of the technique.

Structural data obtained by CD spectroscopy on these proteins again show that mutations do not alter the overall protein fold and that the reduced affinity derives mostly from side chain replacements and not from impairment of the global protein folding (Supplementary Information Figure S3).

### The hydrophobic residues of both HR1 and HR2 domains of PREP1 are essential to drive PREP1 to the nucleus

**Figure 4:**
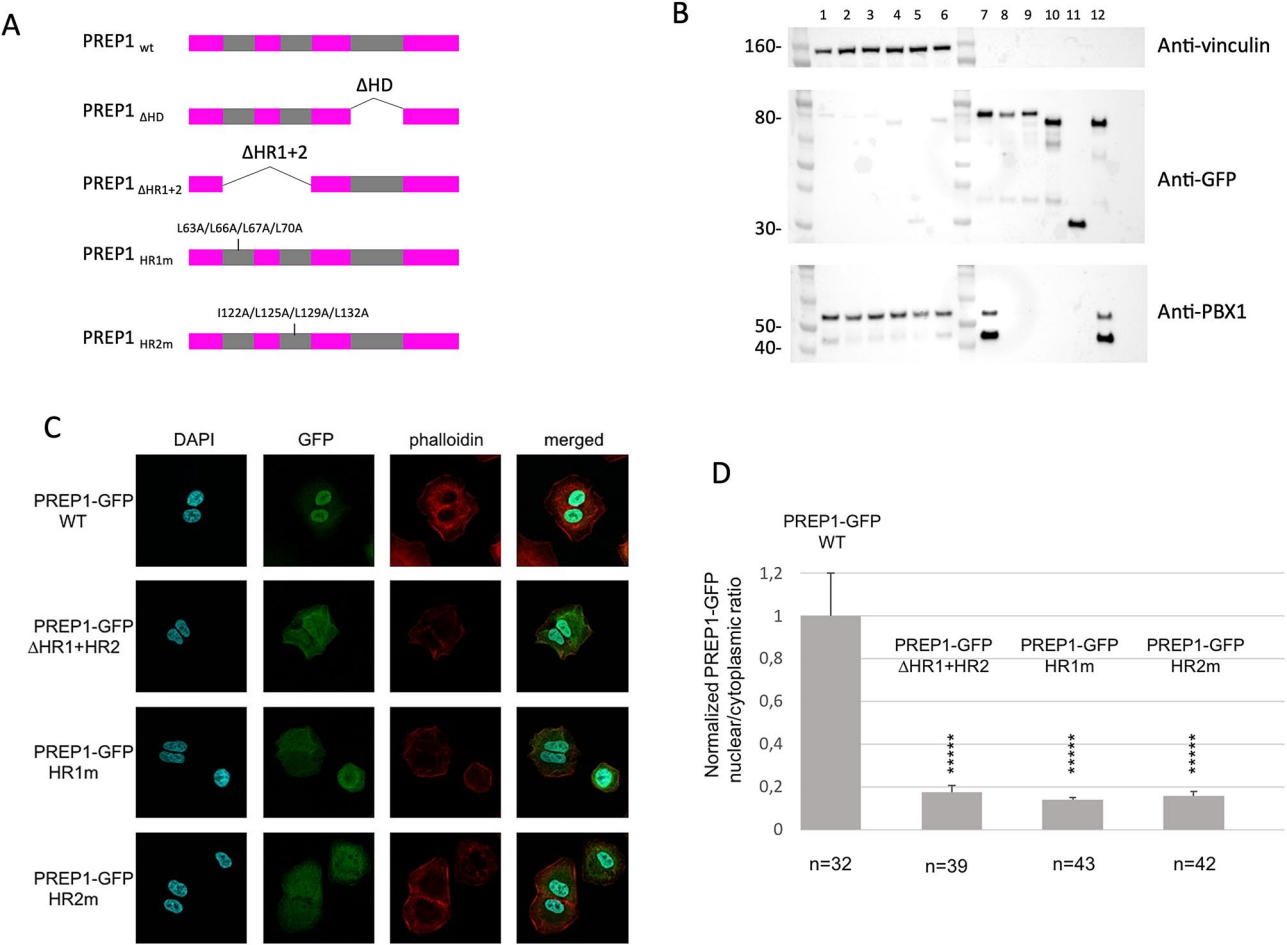
Confocal analysis of PREP1-GFP HR1m and HR2m subcellular localization. **Panel A**: Schematic representation of PREP1-GFP constructs used for immunofluorescence and pull-down experiments. In the scheme, the deleted regions are indicated along with the positions of the quadruple PREP1 mutations HR1m (L63A/L66A/L67A/L70A) and HR2m (I122A/L125A/L129A/L132A). **Panel B**: PBX1 pull-down in total lysates of A549 cells overexpressing wild-type or mutants PREP1-GFP. This panel shows cropped parts of a single immunoblot membrane (the full-length blots are shown in Supplementary Information Figure S4D) stained with different antibodies. The top part shows the anti-vinculin staining, the middle part the anti-GFP and the bottom part the anti-PBX1 staining, as indicated. In all cases, lanes 1–6 are the pull-down inputs; lanes 7–12 the pull-down results. All lysates were supplemented with a total of 1 µg of purified recombinant PBX1. Lanes 1 and 7: lysates of PREP1-GFP wild-type expressing cells; lanes 2 and 8, lysates of PREP1-GFP L63A/L66A/L67A/L70A expressing cells (HR1m); lanes 3 and 9 lysates of PREP1-GFP I122A/L125A/L129A/L132A (HR2m) expressing cells; lanes 4 and 10 lysates of PREP1-GFP ΔHR1 + 2 expressing cells; lanes 5 and 11 lysates of cells expressing GFP alone; lanes 6 and 12 lysates of PREP1-GFP ΔHD expressing cells. **Panel C**: A549 cells were transfected with a plasmid encoding wild-type, or mutants (HR1m, HR2m, ΔHR1 + HR2, or ΔHD) PREP1-GFP. The figure shows illustrative confocal immunofluorescence microscopy images in which the nucleus is identified by DAPI fluorescence and PREP1 by GFP immunofluorescence. The transfected constructs are indicated on the left. PREP1-GFP is in green, DAPI in blue, phalloidin in red. The merged DAPI, GFP and red channels are shown on the right. PREP1-GFP mutants reveal a cytoplasmic localization, compared to the exclusively nuclear localization of PREP1-GFP wild-type. **Panel D**: Bar graphs showing the intracellular distribution of wild-type and mutants PREP1-GFP. Plotted is the normalized nuclear/cytoplasmic GFP quantification, using a minimum 32 individual cells for each construct.The number of cells quantified is reported at the bottom of each histogram bar. T-TEST was performed as described in the Methods section.

Dimerization with PBX1 allows PREP1 and MEIS1 to reach the nucleus and suppresses PBX1 nuclear export through its nuclear export signal^50^. PREP1 has no nuclear localization signal, and no nuclear localization signal has been reported for MEIS, except the homology with Drosophila *Hth*^51,52^. We have therefore tested the ability of PREP1 quadruple mutants to reach the nucleus. We infected lung carcinoma A549 cells, which express low levels of PREP1^3^, with a retroviral vector overexpressing GFP-tagged wild-type or PREP1mutants, and created stably expressing lines. We compared wild-type PREP1-GFP, the previously tested PREP1-GFP ∆HR1 + 2 and PREP1-GFP ∆HD, to the PREP1-GFP HR1m and HR2m mutants described above. All PREP1-GFP-tagged constructs were successfully expressed in A549 cells as judged by immunoblotting (Supplementary Information Figure S4A) and flow-cytometry (Supplementary Information Figure S5). Immunoblot of total lysates of A549 cells overexpressing PREP1 ∆HR1 + 2, HR1m and HR2m showed a lower level of PBX1 (Supplementary Information Figure S5B), with respect to cells overexpressing wild-type PREP1 or PREP1∆HD, in line with PBX1 stabilization by association with PREP1^53^. Therefore, we tested the interaction of the PREP1 mutants with PBX1, by measuring the amount of PREP1-bound PBX1 by GFP pull-down and PBX1 immunoblotting (Fig. 4B). As the PBX1 expression levels are uneven in cells expressing the different PREP1 constructs, before pull-down we incubated the cell lysates with 1 µg of purified recombinant human PBX1. As expected on the basis of previous observations^4^, PREP1 wild-type and PREP1 ∆HD efficiently pulled-down recombinant PBX1 (respectively lanes 7 and 12 of Fig. 4B), while PREP1 ∆HR1 + 2 and the two quadruple point mutants HR1m and HR2m did not (lanes 8, 9 and 10 of Fig. 4B).

Next, we used fluorescence microscopy to determine the subcellular localization of the transfected PREP1-GFP, wild-type and mutants (Fig. 4C). As expected, fluorescence localized almost uniquely to the nucleus with wild-type PREP1, whereas with ΔHR1 + HR2, HR1m or HR2m it was largely found in the cytoplasm. Normalized values of the nuclear/cytoplasmic ratios of PREP1-GFP is shown in Fig. 4D.

Therefore, we conclude that residues L63, L66, L67 and L70 in HR1, and I122, L125, L129 and L132 in HR2 are required to drive PREP1 to the nucleus and therefore must be the main contact points between PREP1 and PBX1 as their replacement with alanine has the same or greater functional effects as the removal of the entire domains.

**Figure 5:**
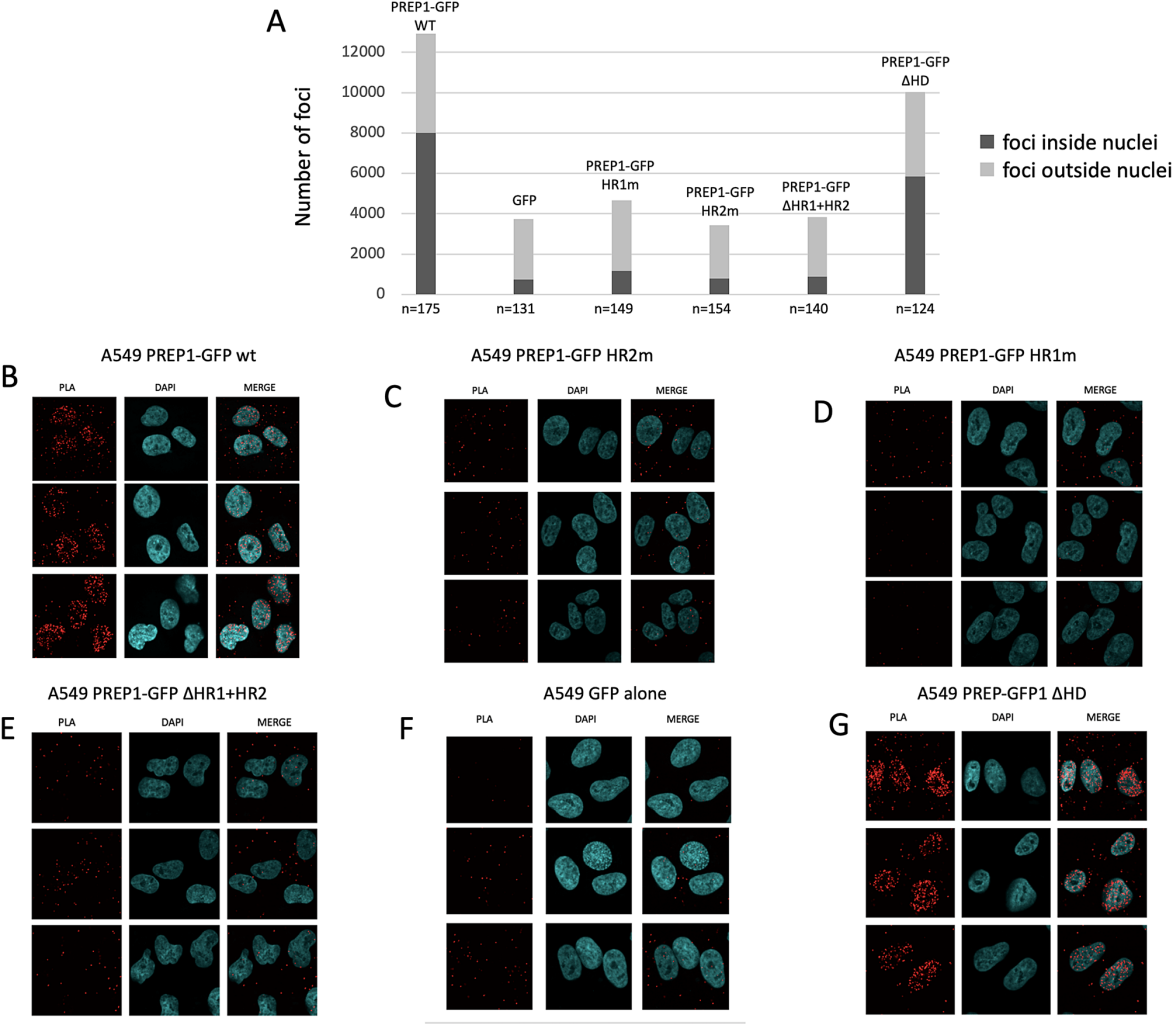
Proximity Ligation Assay of the interaction of PBX1 with wild-type and mutant PREP1**.** The PREP1-PBX1 complex is visualized by PLA in A549 cells transfected with different GFP-PREP1 constructs (indicated) and using anti-PBX1 and anti PREP1 antibodies (see Methods), which generates red foci when the two proteins are in close proximity. **Panel A** shows the number of PLA foci counted in the different cells transfected with the indicated constructs. The data are the average of two independent experiments. Nuclear foci are represented in the histogram in dark grey, cytoplasmic foci in light grey. The number of cells counted is reported at the bottom of each histogram bar. **Panels B-G** show representative confocal images of the PLA experiment in A549 cells transfected with wild-type or mutant PREP1 as indicated. **Panel F**, shows cells in which only GFP was overexpressed to define the background signal. In **panel G**, PREP1 ΔHD nuclear foci are comparable to those of the PREP1 wild-type.

To further confirm this conclusion, we used proximity ligation assays (PLA). This assay allows the in situ detection of target molecules which are in close proximity (< 40 nm) to each other. The method consists in a first step in which single-stranded oligonucleotides are conjugated to specific antibodies to recognize the target proteins; subsequently the signal is amplified by PCR and then DNA is hybridized with complementary oligonucleotides labeled with a fluorophore. Therefore if the target molecules are in sufficient proximity to each other, the DNA signal is amplified and PLA will produce a robust, specific, and visible fluorescence signal^54^. In our PLA set-up, the contiguity of the anti-GFP and anti-PBX1 antibodies becomes visible as red foci. Quantification of the number of nuclear PLA foci showed their almost complete absence in cells transfected with PREP1-GFP mutants HR1m and HR2m (comparable to background PLA foci, obtained with expression of GFP-alone, Fig. 5A) as opposed to their presence in the case of the wild-type PREP1 and of the ΔHD mutant. Specific examples of immunofluorescence of A549 cells transfected with wild-type PREP1 and the various PREP1 mutants are shown in Fig. 5. PREP1∆HD, which only lacks the homeodomain, retains the ability to form nuclear PLA foci with PBX1 indicating an efficient association between the two proteins (Fig. 5B and G).

Altogether, these data corroborate and strengthen the view that the hydrophobic residues substituted in HR1m and HR2m mutants are required for the binding to PBX1.

## Discussion

The results presented in this work confirm the overall type of interaction of PBX1 with PREP1 or MEIS1. Furthermore, the present data provide novel information on the three-dimensional organization of the domains in the heterocomplexes, highlight the relative importance of individual domains and residues involved in the interactions and suggest protein areas that might be targeted to specifically inhibit MEIS1 or PREP1 heterodimerization with PBX1.

### Suggestive spatial organization

Chemical cross-linking analysis strengthens the requirement of the hydrophobic regions of PREP1 and MEIS1 in the interaction with PBX1^37,55^. In addition, it provides an interesting, although very superficial, information on how the individual domains must be organized in space in the heterodimeric molecules (see cartoons, Figure S1C and D). For example, HR1 and HR2 of PREP1 must be located in proximity of PBC-A and PBC-B, respectively; in addition, the homeodomains of PREP1 and PBX1 must be close in space to HR2 and PBC-B (Fig. 1). Indeed, XLMS shows that K65 and K87 (PBC-A) and K195 (PBC-B) are intramolecularly cross-linked with the K242 (homeodomain); likewise, in PREP1, HR2 is intramolecularly cross-linked with its own homeodomain through K134 and K268 (Fig. 2). On the contrary, intramolecular cross-links of MEIS1 homeodomain to its HR1 and HR2 are not evident from our data.

### Relative importance of the individual domains

Our cross-linking data underscore a functional difference between MEIS1 and PREP1 HR1 domains; in the interaction with PBX1, HR1 appears to be less involved in MEIS1 than in PREP1. The lesser involvement of HR1 in MEIS1 may justify the difference in affinity for PBX1 between PREP1 (18 ± 4 nM) and MEIS1 (80 ± 17 nM).

The experiments presented allow to identify the important residues involved in the binding of PREP1 to PBX1, as shown by the mutations HR1m and HR2m. We have excluded by CD analysis that the strong effect we observe in quadruple mutant of the HR2 of PREP1 is due to a grossly altered conformational change. In addition, mutational analysis of PREP1 excludes any contribution of the “HR3” domain (stretch 242–252) in the binding to PBX1.

Experimental evidences in the literature demonstrate a contribution only of PBC-A in the PREP1 and MEIS1 interactions. Even though the present work is mostly focused on PREP1, we also derive information demonstrating for the first time a direct involvement of PBC-B in the interaction. Indeed, XLMS underlines a solid connection between PBC-B and the HR2 domains of both PREP1 and MEIS1 through K153 of PBX1 (Figs. 1A and 1C), even though we could not identify a heptad repeat in the region surrounding K153 of PBX1. The contribution of PBC-B may be more important in MEIS1 than in PREP1 interaction, as shown by the deletion of Δ197-211 (Figs. 3B and 3C). This was unexpected, as XLMS evidences in PREP1 a direct connection between PBC-B (K195) and HR2 (K151/K153). However, K195 (PBC-B) is linked to K268 (PREP1 homeodomain), and intramolecularly to K242 (PBX1 homeodomain). Perhaps, in the case of PREP1, PBC-A interacts with HR1 while PBC-B with HR2 and homeodomain; instead, in the case of MEIS1, HR2 contacts both PBC-A and PBC-B. We can speculate on a different function of PBC-B in the interaction with PREP1 versus. MEIS1. In the interaction with PREP1, PBC-B might have a more structural role, for example it might contribute to bring together the two homeodomains, whereas in the interaction with MEIS1 it might directly contribute to the binding. However, while these speculations still need to be proven, unexplored differences between PREP1 and MEIS1 binding mode to PBX1 cannot be excluded. Unfortunately, we have no XLMS confirmations of an interaction of the homeodomain of MEIS1 with K195 of PBX1, because MEIS1 homeodomain becomes extremely fragmented by trypsin/Glu-C digestion. We can nevertheless conclude that there are two distinct binding sites for PREP1 and MEIS1 in PBX1: one in PBC-A (heptad 77–84) and two in PBC-B (the region around K153 and the heptad 197–211).

In conclusion, based on the evidences obtained by multiple methods including XLMS (Fig. 1), mutagenesis and binding studies (Figs. 2 and 3), proximity ligation assays (Fig. 5) and immunofluorescence analysis of the subcellular localization of mutant proteins (Fig. 4), we have therefore: 1) identified two regions in PREP1 whose mutation prevents the interaction with PBX1, and through mutagenesis we have underlined which specific residues are involved; 2) outlined for the first time a functional difference between the HR1 domains of MEIS1 and PREP1; 3) highlighted a prevalent role played by PBC-B in the binding to MEIS1. All these findings might be potentially exploited to pharmacologically target and inhibit MEIS1-PBX1 complex.

### Possible applications in drug discovery

A more detailed knowledge of the PREP-PBX and MEIS-PBX interaction surfaces opens up the possibility of interesting applications in drug discovery. The formation of PREP1-PBX1 and MEIS1-PBX1 dimers occurs in the cytoplasm and allows their transport to the nucleus where they can bind DNA to exert their physiological role. As PREP1 has no nuclear localization signals^27^, the interaction with PBX1 is essential to reach the nucleus. The same appears to be true for MEIS1. Therefore, disruption of the interaction with PBX1 can block PREP1 and MEIS1 nuclear translocation, and consequently their function. This can be exploited in therapy, for example in the Mixed Lineage Leukemia in which the PBX-MEIS complex appears to have a very important role, together with HOXA9^21,22,56^.

ChIPseq studies in the mouse embryo and cell lines have previously shown that the binding specificity of PBX1 depends on the nature of the interactor, i.e. MEIS1 or PREP1^7^. One of the most important and frequent partners of PBX and MEIS are the HOX proteins, that are essential in determining cell identity but also very important in leukaemia, in particular the HOX paralog group 9. MEIS-PBX and PREP-PBX complexes participate in trimeric DNA-binding complexes with anterior members of the HOX group^37,55,57–59^, because PBX and HOX interact at the level of the homeodomains^60,61^. Since MEIS1 or PREP1 interact with PBX1 at the amino-terminal side^37,55,62–64^, a ternary complex can be formed. Indeed, ChIPseq has shown that a large number of MEIS1 DNA-binding sites coincides with those of HOXC9 while this association is much less for PREP1^7^. Therefore, interfering with the formation of the MEIS-PBX complexes might also affect the activity of the HOX paralog group 9 proteins that are involved in cancer and leukaemia. Furthermore, the role of HOX proteins in increasing the oncogenic effect of MEIS1 is likely to also require PBX as the latter is essential in HOX proteins DNA-binding specificity^65^. Therefore, the disruption or the prevention of MEIS-PBX dimers and of MEIS-HOX-PBX trimers must be considered a therapeutic goal. To date, the only effective inhibitors of HOX-PBX binding are the HXR9 peptide and its derivatives, originally derived from the HOXA9-PBX1 interacting peptide^66^. HXR9 was shown to inhibit the growth of a range of tumours in mouse xenograft models, including non-small cell lung, breast, ovarian, and prostate cancer, mesothelioma, melanoma, and meningioma^66–72^. HXR9 is supposed to act by preventing the binding of HOX-PBX complexes to the DNA. This type of action requires that a drug must be able not only to enter the cell but also localize itself in the nucleus. Therefore, targeting the formation of MEIS-PBX complex in the cytoplasm might be more efficacious. It must be noticed that the use of the HXR9 in clinics has not been reported. Moreover, targeting the MEIS homeodomain by using small molecule derived from compound collections screenings seems effective^73^, but does not impair dimerization with PBX nor nuclear localization of MEIS.

Finally, one must consider that the stability of MEIS and PBX proteins depends on their ability to dimerize. The overall level of PBX1 is decreased in the *Prep1*^*i/i*^ embryos^14^. PBX2 half-life is increased in cells overexpressing PREP1 because it prevents its proteasomal degradation^53^. Likewise, in mice, PBX3 protects MEIS1 from proteasomal degradation, increases MEIS1 affinity for HOXA9 and induces endogenous *Meis1* transcription^20^. Therefore, the prevention of dimerization might have the additional advantage of decreasing also the level of the monomeric proteins.

In conclusion, our work identifies for the first-time an additional potential target for preventing the formation of a MEIS-PBX dimer. As this interaction would occur in the cytoplasm, a drug affecting the heterodimer formation would not only prevent its transcriptional activity, but also leave MEIS and PBX in monomeric form in the cytoplasm, i.e. unable to reach the nucleus, and hence be directed to degradation. Such drugs might be more performing than those acting on the binding to DNA, because the inability to reach the nucleus is likely to induce PBX instability^14^ and this would increase even more a possible therapeutic effect.

## Materials and methods

### Protein alignment and secondary structure prediction

Protein alignments were done using CLUSTAL W^74^, and secondary structure prediction using NetSurfP-2.0^75^.

### Cloning and mutagenesis

Single proteins (PBX1_1-430_, MEIS1_1-390_, and PREP1_1-436,_, named PBX1, MEIS1 and PREP1, respectively) and protein complexes (PBX1_1-430_-PREP1_1-436_, PBX1_1-308_-PREP1_1-344_) were all subcloned in pGEX-6P-2RBS (GenBank: KM817768), that support di-cistronic expression as described previously^47^, with PBX1 in the first cassette and PREP1 in the second cassette. DNA encoding full length MEIS1 was amplified by PCR with primers containing BglII/XhoI (New England Biolabs) restrictions sites.

Primers used for cloning MEIS1 into pGEX were:

MEIS1 1 > forward 5′-CGCAGATCTATGGCGCAAAGGTACGACGATCTACCC-3′.

MEIS1 < 390 reverse 3′-CGCTCGAGTTACATGTAGTGCCACTGCCCCTCCATG-5′.

The amplified PCR product was inserted in pGEX-6P-2RBS vector. The plasmid was transformed into One-Shot chemically competent TOP10 E. coli cells (Thermo Fisher Scientific, Italy). The sequence of the insert was validated by sequencing of DNA clones.

Single‐point mutants and multiple mutants were prepared by PCR using primers listed in Supplementary Information Tables S1 and S2. Deletion mutants were cloned using overlap PCR, as described in^76^. All constructs were verified by sequencing them in both directions.

pBABE-puro was a gift from Hartmut Land & Jay Morgenstern & Bob Weinberg (Addgene plasmid #1764). pBABE-puro-PREP1-GFP wild-type and mutants were obtained by first amplifying the PREP1 sequences with primers PREP1FX/PREP1RK and cloning them using XhoI/KpnI (New England Biolabs) in pEGFP-N1 vector. Retroviral vectors were generated by amplification with primers PREP1SnF/GFPSaR of the sequences corresponding to the fusion proteins and cloning using SnaBI/SalI (New England Biolabs) into the pBABE-puro vector (Addgene#1764)^77^. The initial templates were: the pGEX-PREP1 vectors described above for PREP1 wild-type and the quadruple mutants in the HR1 and HR2 regions, and pMSCV vectors previously described^4^ for the deletion mutants ΔHD and ΔHR1 + 2. To generate the control vector pBABE-puro-GFP, pBABE-puro-PREP1-GFP was amplified with oligos GFPSnF/GFPSaR and the PCR product was inserted in pBABE-puro using SnaBI/SalI restriction sites. Primers used to subclone PREP1-GFP into pBABE retroviral vector are listed in Supplementary Information Table S3.

### Cell culture and retroviral infection

A549 cells were grown in DMEM (Lonza) + 10% FBS + 2 mM L-glutamine. For the retroviral transduction, Phoenix-Ampho cell line was used. Briefly, Phoenix-Ampho cells were transiently transfected with the retroviral vectors pBABE-puro described above by standard calcium-phosphate protocol. Cell supernatants containing retroviruses were filtered using 0.45 µm filters and employed to perform two rounds of infection. Two days after infection, cells were selected by addition of puromycin (1,1 µg/mL) to the culture medium. To check for the expression of the PREP1-GFP-constructs, 5 days post-infection A549 cell lines were assayed by western blot analysis of cell lysate using anti-GFP antibody (AbCam #Ab290) (Supplementary Information Figure S4A) and by flow-cytometry analysis (Supplementary Information Figure S5). Flow cytometry instrument used was FACSCalibur (Becton Dickinson), and the data were analyzed using Kaluza Analysis Software (Beckman Coulter). For PBX1 expression in western blot we used anti-PBX1 (Cell Signaling #4342) (Supplementary Information Figures S4B).

### Expression in *E. coli* and purification of recombinant proteins

Expression and purification of PREP1, MEIS1 and PBX1 were carried out as described previously^47^. For ELISA assay, GST-free proteins were run on SDS-PAGE (Supplementary Information Figure S6) and then harvested and stored at − 80 °C until use. GST-free PBX1-PREP1 full-length complex was loaded into ion exchange buffer (20 mM Tris pH 7.4, 0.1 M NaCl, 10% glycerol, 0.5 mM EDTA, 0.5 mM EGTA and 1 mM dithiothreitol (DTT)) to a final NaCl concentration of 0.1 M and run on a Resource Q (GE Healthcare) anion exchange column. C-terminal truncated (PBX1_1-308_-PREP1_1-344_) complex was run on a Resource S (GE Healthcare) cation exchange column. In both cases the proteins were eluted using a 0.1–1.0 M NaCl gradient. Proteins used for cross-linking and proteolysis analysis were further purified by size exclusion chromatography on a Superose 6 10/300 column (GE Healthcare) equilibrated in 20 mM Tris pH 7.4, 0.3 M NaCl, 5% glycerol, 0.5 mM EDTA and 1 mM DTT and eluted at a flow rate of 0.2 ml/min.

### Cross-linking mass-spectrometry

#### Sample preparation and protein digestion

Purified PBX1-PREP1 complex at 1 mg/ml was dialyzed 2 h at room temperature in 50 mM borate-buffered saline. After dialysis, 1 mM Bis(sulfosuccinimidyl) suberate sodium salt (BS^3^) (Sigma-Aldrich) was added. Cross-linking reaction was performed for 1 h at room temperature with shaking at 1000 rpm as described in^78^ . Reaction was stopped by adding 100 mM sodium bicarbonate (final concentration) and incubated at room temperature for 10 min. Cross-linked proteins were separated by SDS-PAGE electrophoresis and stained by coomassie. Gel slices were excised from the gel, reduced with 10 mM DTT, alkylated with 55 mM Iodoacetamide (IAA) and finally digested overnight with Glu-C and then with Trypsin^79^, before peptides were desalted on StageTip C18^80^.

### nLC-ESI–MS/MS analysis

Samples were analyzed as described in^81^ with few exceptions. In particular, 4 µL of peptide mixture from each sample were analyzed on a nLC–ESI–MS/MS quadrupole Orbitrap QExactive-HF mass spectrometer (Thermo Fisher Scientific). Peptides separation was achieved on a linear gradient from 95% solvent A (2% ACN, 0.1% formic acid) to 60% solvent B (80% acetonitrile, 0.1% formic acid) over 48 min, and from 60 to 100% solvent B in 2 min at flow rate of 0.25 µL/min on a UHPLC Easy-nLC 1000 (Thermo Fischer Scientific) connected to a 23 cm fused-silica emitter of 75 µm inner diameter (New Objective, Inc. Woburn, MA, USA), packed in-house with ReproSil-Pur C18-AQ 1.9 µm beads (Dr. Maisch GmbH, Ammerbuch, Germany). MS data were acquired using a data-dependent top 15 method for HCD fragmentation. Survey full scan MS spectra (300–1800 Th) were acquired in the Orbitrap with 60,000 resolution, AGC target 3^e6^, IT 20 ms. For HCD spectra, resolution was set to 15,000 at *m*/*z* 200, AGC target 1^e^^5^, IT 160 ms; NCE 28% and isolation width 1.4 m/*z*.

### Data analysis

For identification, raw data were processed with pLink 2 ver. 2.3^82^ searching against the database containing the sequences of PREP1_1–344_-PBX_1–308_, and MEIS1_1–390_-PBX1_1–308_; Glu-C and trypsin specificity was indicated and up to five missed cleavages were allowed. BS^3^ was selected as cross linker, cysteine carbamidomethyl was used as fixed modification and methionine oxidation as variable. Mass deviation for MS/MS peaks was set at 20 ppm. Filter tolerance was set at 10 ppm, while the FDR was set to 0.05, corresponding to an E-value of 0.001. For manually curated analysis only those spectra with at least 4 consecutive b or y ions for each cross-linked peptide were considered as confidently identified^83^. The full-list of cross-linked peptides and relatives E-values are available in supplementary materials.

In manually curated analysis, only those spectra with *E*-value < 0.001 (corresponding to FDR < 0.05) were considered confidently identified; moreover, we required the majority of the ions of the spectrum assigned and 4 consecutive b or y ions for each cross-linked peptide observed^83^. The full-list of cross-linked peptides and relatives E-Values are available in Supplementary Information Tables S4–S11. Raw files and the annotated spectra (.tif files) are available via PeptideAtlas (PASS01497).

### ELISA binding

ELISAs were performed as previously reported^48^, introducing slight modifications. Briefly, PBX1 (wild-type) was coated on 96-well plates at 0.1 µM in 100 mM Tris–HCl, pH 7.5, for 16 h at 4 °C. After coating, the plates were washed three times with PBS-T buffer (3.2 mM Na_2_HPO_4_, 0.5 mM KH_2_PO_4_, 1.3 mM KCl, 135 mM NaCl, containing 0.05% Tween 20, pH 7.4) and blocked with 300 µL of PBS containing 3% BSA for 90 min at 37 °C. After washing, solutions of GST-fused PREP1 and MEIS1 wild-type proteins, mutant variants (HR1m and HR2m) and the GST protein, used as control, at concentrations ranging between 0.23 nM and 0.5 µM, were added to coated wells and incubated for 1 h at 37 °C, in 100 mM Tris–HCl pH 7.5. After washing 100 µL of anti-GST antibody (Bio-Rad #MCA1352) diluted 1:250 with 100 mM Tris–HCl pH 7.5 was added. After 1 h incubation at 37 °C, the plates were washed again with PBS-T, and 100 µL of anti-mouse IgG-HRP (HRP, horseradish peroxidase) (Bio-Rad #AAC10P) diluted 1:1000 in PBS was added. Finally, after 1 h incubation and subsequent washing, 100 µL of chromogenic substrate (0.4 mg/mL *o*-phenylenediamine in 50 mM sodium/phosphate/citrate buffer, pH 5.0, containing 0.4 mg/mL H_2_O_2_) was added and the reaction stopped by adding 50 µL of 2.5 M H_2_SO_4_ to each well. The absorbance was measured at 495 nm. Results were measured in triplicate from three independent assays. Results are reported as subtraction between signals obtained with the proteins and that with the GST. Data were fitted with GraphPad Prism, version 5.0, GraphPad Software Inc, San Diego, California. Binding curves were all best fitted with a one-site binding model.

### Circular Dichroism spectroscopy

CD spectra were recorded in the range 190–260 nm on a J-810 spectropolarimeter (Jasco International Co. Ltd., Tokio, Japan) as reported in^48^. Peptide and protein samples were prepared at 1 µM in 10 mM phosphate buffer pH 7.4. Spectra were acquired in triplicate under the following experimental conditions: 1 mm path length (quartz cuvette), scan speed 20 nm/min, band width 1.0 nm, resolution 0.2 nm, sensitivity mdeg, response time 4 s. Spectra were averaged, blank subtracted and the CD signal converted to residue ellipticity expressed in deg × cm^2^ × dmol^−1^ × res^−1^.

### Time-resolved fluorescence immunoassay (TR-FIA)

OptiPlate-96 F HB, black 96-well (Perkin-Elmer Corp., Waltham, Massachusetts) were coated over night at 4 °C with 100 µl of 1 µg/mL monoclonal anti-PBX1 in coating buffer (0.1 M Na Carbonate pH 9.8). After the overnight, the wells were washed 3 times with PBS-T, and then non-specific binding sites were blocked for 2 h at room temperature with 200 µL of The Blocking Solution (CANDOR Bioscience, Wangen, Germany). After washing 3 times with PBS-T, 100 µL of standards (PBX1, MEIS1, or PREP1 at 0,5 mg/mL) wild-type or mutant samples in combination with wild-type proteins diluted to 10 µg/mL in 1% BSA PBS were added and incubated. For the PBX1 mutants screening, MEIS1 or PREP1 wild-type were incubated with PBX1 mutants for 1 h on ice before being added to the TR-FIA plate. In the same way, for PREP1 mutant screening, PBX1 wild-type was incubated with PREP1 mutants. After 1 h at room temperature, wells were washed 3 times before adding 100 µL of rabbit polyclonal anti-PREP1, or biotinylated goat polyclonal anti-MEIS1/2 antibody (at 0.5 µg/mL). After 1 h, the plate was washed 5 times with PBS-T and 100 µL of detection Eu^3+^-conjugated anti-rabbit antibody (DELFIA, Perkin Elmer #AD0105) were added to the wells containing the anti-PREP1 antibody (DELFIA, Perkin-Elmer Corp.) diluted 1:5000 in 1% BSA-PBS; in the case of MEIS1 detection Eu^3+^-conjugated anti-streptavidin antibody (DELFIA, Perkin-Elmer Corp.) was added diluted 1:10,000 in 1% BSA-PBS. After 1 h, the plate was washed 8 times with PBS-T before incubating for 15 min with 100 µL of DELFIA Inducer (DELFIA Enhancement Solution, Perkin-Elmer). The Eu^3+^-label was detected by measuring time-resolved fluorescence intensity using an Envision Xcite plate reader (Perkin Elmer Corp.) employing the DELFIA label protocol. Monoclonal anti-PBX1 antibody was purchased from Sigma Aldrich (#SAB1404170), polyclonal anti-PREP1 antibody from Santa Cruz Biotechnology (#sc6245), polyclonal C17 anti-MEIS1/2 antibody (Santa Cruz Biotechnology) was conjugated (Antibody Facility, Cogentech, Milan) to the NHS-LC-biotin (Thermo-Scientific). The values reported represent the means of four independent experiments each conducted in triplicate. Means and standard deviations were calculated in Excel, which was also used to plot the graphs (we applied STDEV*.*P function, used in calculating the standard deviation for an entire population). The data are reported as normalized to wild-type protein binding (taken as 1). Type 2 (homoscedastic)T-TEST was performed using one-tail function, to determine if there was a significant difference between each mutant binding datasets against the wild-types binding datasets. We assigned * for values below 0.05 (90% significance), ** for values below 0.025 (95% significance), *** for values below 0.01 (significance 98%), **** for values below 0.005 (99% significance), and ***** for values below 0.001 (> 99% significance).

### In situ proximity ligation assay (PLA)

For in situ proximity ligation assay (PLA, Duolink, Sigma), cells were labeled according to the manufacturer's instructions, and as described in^54^. Briefly, A549 cells expressing PREP1-GFP wild-type or PREP1-GFP mutants were fixed in PFA for 10 min at room temperature. After incubation with primary antibodies (Anti-PBX1 (Cell Signaling #4342), Anti-GFP (AbCam #Ab290)), appropriate PLA probes (secondary antibodies conjugated with oligonucleotides) were added to the samples. Ligation of the oligonucleotide probes that were in proximity (less than 40 nm) was then performed, after which fluorescently labeled oligonucleotides were added together with a DNA polymerase to generate a signal detectable by a fluorescence microscope. In order to find the number of foci inside and outside nuclei, a custom Fiji^84^ plugin was developed. The plugin identifies foci using the ImageJ’s Find Maxima^85^ algorithm on the red channel image after background removal (using the rolling ball algorithm^86^). The nuclei are recognized on the DAPI channel image, enhanced with a gaussian blur filter, applying the Huang’s threshold method. Then, the plugin counts the foci inside and outside the nuclei. The values reported represent the count of two independent experiments and for each condition we quantified 125–175 cells.

### Confocal microscopy acquisition and images analysis

In order to evaluate the nuclear localization of GFP-tagged PREP1 a Fiji^84^ plugin was developed. The plugin finds the nuclei on the DAPI channel using the Otsu’s thresholding algorithm^87^, after background removal^88^ and the application of a gaussian filter. Then, for each nucleus found the mean intensity of the GFP signal is extracted after background removal. For each cell, the cytoplasmic mean intensity is extracted from a drawn by hand region. The plugin reports the ratio between the nuclear and the cytoplasmic signal for each cell. To mark the cell perimeter, we stained the cytoskeleton using Alexa Fluor 647 phalloidin (Thermo Fisher Scientific). Type 2 (homoscedastic) T-TEST was performed using one-tail function, to determine if there was a significant difference between each mutant binding datasets against the wild-types binding datasets. We assigned * for values below 0.05 (90% significance), ** for values below 0.025 (95% significance), *** for values below 0.01 (significance 98%), **** for values below 0.005 (99% significance), and ***** for values below 0.001 (> 99% significance).

### Pull-down of PBX1 and PREP1 in A549 cells

2000 µg of total lysate were immunoprecipitated using anti-GFP magnetic beads (ChromoTek GFP-Trap, Chromotek, Planegg-Martinsried, Germany) following the manufacture’s protocol. For the western blot, we employed anti PBX1 (Cell Signaling #4342), anti-GFP (AbCam #Ab290), anti-vinculin (Sigma Aldrich #V4505) antibodies.

## Supplementary information

Supplementary information.
