# Supplementary material for: Mapping the native interaction surfaces of PREP1 with PBX1 by cross-linking mass-spectrometry and mutagenesis
Source: Sci Rep. 2020 Oct 8;10:16809. doi: 10.1038/s41598-020-74032-w (PMC7545097; doi:10.1038/s41598-020-74032-w)
Supplement: Supplementary file 1 — Supplementary information. [file 41598_2020_74032_MOESM1_ESM.pdf]

Supplementary Material

Mapping the native interaction surfaces of PREP1 with PBX1 by cross-linking mass-spectrometry and mutagenesis.

Chiara Bruckmann [1*], Simone Tamburri [1,3], Valentina De Lorenzi [1,4], Nunzianna Doti [2], Alessandra Monti [2], Lisa Mathiasen [1], Angela Cattaneo [1,5], Menotti Ruvo [2], Angela Bachi [1], and Francesco Blasi [1*]

1 IFOM (Foundation FIRC Institute of Molecular Oncology), via Adamello 16, 20139, Milan, Italy

2 Institute of Biostructures and Bioimaging (IBB)-CNR; via Mezzocannone 16, 80134, Naples, Italy

3 Present Address: Department of Experimental Oncology, European Institute of Oncology, via Adamello 16, 20139 Milano, Italy

4 Present Address: Center for Nanotechnology Innovation@NEST, Istituto Italiano di Tecnologia, Piazza San Silvestro 12, 56124, Pisa, Italy

5 Present Address: Cogentech S.R.L. Benefit Corporation IT Via Adamello 16, 20139 Milan, Italy

* co-corresponding authors: chiara.bruckmann@ifom.eu, francesco.blasi@ifom.eu

Supplementary Figures

Figure S1

Panel A: Cross-linking mass-spectrometry workflow.

Panel B: SDS-PAGE of purified samples (PREP1-PBX1 and MEIS1-PBX1 complexes) and their BS^3 cross-link. In lane 1, the PREP1-PBX1 purified complex. In lanes 2-4 the PREP1-PBX1 cross-linked samples, used for mass-spectrometry analysis. In lane 5, the MEIS1-PBX1 purified complex. In lanes 6-8 the MEIS1-PBX1 cross-linked samples, used for mass-spectrometry analysis. The experiment was repeated twice, and the SDS PAGE shown is representative of one of the two experiments.

Panel C: cartoon model summarizing the overall folding of the PBX1-PREP1 complex, as comprehended from the XLMS data. The most relevant inter molecular interactions between domains are highlighted with a star, and the corresponding peptides identified are listed. In grey the PBX1 model, in purple the PREP1 model.

Panel D: cartoon model summarizing the overall folding of the PBX1-MEIS1 complex, as understood from the XLMS data. The most relevant inter molecular interactions between domains are highlighted with a star, and the corresponding peptides identified are listed. In grey the PBX1 model, in azure the MEIS1 model.

Figure S2

Panel A and **B**: HR1 domains are highlighted in the red box, HR2 domains in the green box and the homeodomain (HD) is highlighted in the magenta box. The HR3 domain of PREP1 is highlighted in the dark green box. Stars above and below the sequence indicate the reactive lysines (K) in the cross-linking experiments. Heptad repeats of PREP1 and MEIS1 are highlighted in red.

Panel C: The PBX1,2,3 alignment. The PBC-A domain is highlighted in the grey box, the PBC-B in the orange box and the homeodomain (HD) in the magenta box. The dark-green boxes show the heptad repeats in the PBC-A analysed by mutations, while the red box shows the heptad repeat in the PBC-B domain. Stars above the sequence indicate the reactive lysines (K) in the cross-linking experiments.

Figure S3

Circular Dichroism (CD) spectroscopic analysis of PREP1 and PBX1 proteins used in this study. All PREP1 mutants analyzed are those tested in the binding assays reported in **Figures 2C** and **2D**. All PBX1 deletion mutants analyzed are those tested in the binding assays reported in Figure 3B and **3C**. All PBX1 point mutants analyzed are those tested in the binding assay in **Figure 3D**.

Panel A: Overlay of CD spectra of wild-type PREP1 and point-mutants in the HR1 region.

Panel B: Overlay of CD spectra of wild-type PREP1 and point-mutants in the HR2 region

Panel C: Overlay of CD spectra of wild-type PREP1 and point-mutants in the HR3 region

Panel D: Overlay of CD spectra of wild-type PREP1 and the quadruple mutants HR1m and HR2m.

Panel E: An overlay of CD spectra of the wild-type protein and of the Δ43-53, Δ77-84, Δ105-113 and Δ197-204 analogs is shown.

Panel F: Overlay of CD spectra of wild-type PBX1 and point-mutants in I43, I46, L47, I50 and I53 within the PBC-A region

Panel G: Overlay of CD spectra of wild-type PBX1 and point-mutants in L77, V80, L81 and I84 within the PBC-A region

Panel H: Overlay of CD spectra of wild-type PBX1 and point-mutants in L105, L108 and L112 within the PBC-A region.

Panel I: Overlay of CD spectra of wild-type PBX1 and point-mutants in I197, I204 and I211 within the PBC-B region.

Figure S4

Expression levels of PREP1-GFP constructs in total lysates after retroviral infection of A549 cells. Immunoblot with anti-GFP (**Panel A**), anti-PBX1 (**Panel B**) and anti-vinculin (**Panel C**). In Panel B, above the PBX1 signal, it is also visible at 160 KDa the signal of vinculin, as anti-PBX1 was probed after vinculin.

In **Panel D** are shown the whole membranes of the immunoblots shown in **Figure 4B**.

Figure S5

Flow-cytometry analysis of A549 cells expressing wild-type and mutant PREP1-GFP fusion proteins used for the pull-down and for the immunofluorescence experiments. A549 were retrovirally transduced with wild-type PREP1-GFP (**Panel A**); PREP1-GFP HR1m (L63A/L66A/L67A/L70A) (**Panel B**); PREP1-GFP HR2m (I122A/L125A/L129A/L132A), (**Panel C**); and PREP1-GFP ΔHR1+2 (**Panel D**) for stable expression. In addition, A549 were transduced with GFP (**Panel E**). In **Panel F** are shown control, not infected, cells. The figure shows the percentage of expression for PREP1-GFP wild-type and mutants in A549 cells.

Figure S6

SDS-PAGE of GST-affinity purified heterologous proteins expressed in *E. Coli* and of the constructs used for time-resolved fluorescence immunoassay (TR-FIA) and ELISA. Details of the samples purified are indicated next to each SDS-PAGE.

Supplementary Tables

Supplementary Table S1: List of primers used for PREP1 mutagenesis

Mutation	Forward 5’->3’	Reverse 5’->3’
PREP1 L63A	CAACAAAGCTAATAATGGAAATGCTGGATGCCTATAAATGGCCTGC	GCAGGCCATTTATAGGCATCCAGCATTTCCATTATTAGCTTTGTTG
PREP1 L66A	CACATTTTTCAAACAACAAAGCTAATGCTGGAAATAGTGGATGCCTATAAATGG	CCATTTATAGGCATCCACTATTTCCAGCATTAGCTTTGTTGTTTGAAAAATGTG
PREP1 L67A	TTTCAAACAACAAAGCTGCTAATGGAAATAGTGGATGCCTATAAATGGCCTG	CAGGCCATTTATAGGCATCCACTATTTCCATTAGCAGCTTTGTTGTTTGAAA
PREP1 L69A	TAGATTGTTCACATTTTTCAAACAACGCAGCTAATAATGGAAATAGTGGATGCC	GGCATCCACTATTTCCATTATTAGCTGCGTTGTTTGAAAAATGTGAACAATCTA
PREP1 L70A	CATCCACTATTTCCATTATTAGCTTTGGCGTTTGAAAAATGTGAACAATCTACACA	TGTGTAGATTGTTCACATTTTTCAAACGCCAAAGCTAATAATGGAAATAGTGGATG
PREP1 L117A	TGGATTGCTTTTACCATTGCATTATCAGTTTCTGGATCTTCACAAAAGAAAGG	CCTTTCTTTTGTGAAGATCCAGAAACTGATAATGCAATGGTAAAAGCAATCCA
PREP1 I122A	GAATGCGCAAAACCTGGGCTGCTTTTACCATTAAATTATCAGTTTCTGGAT	ATCCAGAAACTGATAATTTAATGGTAAAAGCAGCCCAGGTTTTGCGCATTC
PREP1 L124A	AATGGTAAAAGCAATCCAGGCTTTGCGCATTCATCTTCTTG	CAAGAAGATGAATGCGCAAAGCCTGGATTGCTTTTACCATT
PREP1 L125A	CAAGAAGATGAATGCGCGCAACCTGGATTGCTTTTACCATTAAATTATCAGT	ACTGATAATTTAATGGTAAAAGCAATCCAGGTTGCGCGCATTCATCTTCTTG
PREP1 L129A	ACCTTTTCCAGCTCAAGAGCATGAATGCGCAAAACCTGGAT	ATCCAGGTTTTGCGCATTCATGCTCTTGAGCTGGAAAAGGT
PREP1 L130A	CCTTTTCCAGCTCAGCAAGATGAATGCGCAAAACCTGGAT	ATCCAGGTTTTGCGCATTCATCTTGCTGAGCTGGAAAAGG
PREP1 L132A	GAGTTCGTTAACCTTTTCCGCCTCAAGAAGATGAATGCGC	GCGCATTCATCTTCTTGAGGCGGAAAAGGTTAACGAACTC
PREP1 L245A	GATGCTGAGATCTTGGTTTGCCTGTAACTGAAGCTGGGAG	CTCCCAGCTTCAGTTACAGGCAAACCAAGATCTCAGCATC
PREP1 L249A	TGATGCAAGATGCTGGCATCTTGGTTTAACTGTAACTGAAGCTGG	CCAGCTTCAGTTACAGTTAAACCAAGATGCCAGCATCTTGCATCA
PREP1 L251A	GAACCATCATCTTGATGCAAGGCGCTGAGATCTTGGTTTAACTG	CAGTTAAACCAAGATCTCAGCGCCTTGCATCAAGATGATGGTTC
PREP1 L252A	AGATGAACCATCATCTTGATGCGCGATGCTGAGATCTTGGTTTAAC	GTTAAACCAAGATCTCAGCATCGCGCATCAAGATGATGGTTCATCT

Supplementary Table S2: List of primers used for PBX1 mutagenesis

Mutation	Forward 5'->3'	Reverse 5'->3'
PBX1 I43A	GCTGTAAAATGTCTCCAGCGTCCTGCTTCCTCCCGC	GCGGGAGGAAGCAGGACGCTGGAGACATTTTACAGC
PBX1 I46A	GATGGTCATAATTTGCTGTAAAGCGTCTCCAATGTCCTGCTTCCTC	GAGGAAGCAGGACATTGGAGACGCTTTACAGCAAATTATGACCATC
PBX1 L47A	GTCATAATTTGCTGTGCAATGTCTCCAATGTCCTGCTTCCTCC	GGAGGAAGCAGGACATTGGAGACATTGCACAGCAAATTATGAC
PBX1 I50A	ACTCTGGTCTGTGATGGTCATAGCTTGCTGTAAAATGTCTCCAATG	CATTGGAGACATTTTACAGCAAGCTATGACCATCACAGACCAGAGT
PBX1 I53A	CTCATCCAAACTCTGGTCTGTGGCGGTCATAATTTGCTGTAAAATG	CATTTTACAGCAAATTATGACCGCCACAGACCAGAGTTTGGATGAG
PBX1 L77A	TTTCACACAACACATTAAACGCGGCAGGCTTCATTCTGTGGC	GCCACAGAATGAAGCCTGCCGCGTTTAATGTGTTGTGTGAAA
PBX1 V80A	TTTTTTCTTTGATTTCACACAACGCATTAAACAAGGCAGGCTTCATT	AATGAAGCCTGCCTTGTTTAATGCGTTGTGTGAAATCAAAGAAAAAA
PBX1 L81A	AACTGTTTTTTCTTTGATTTCACACGCCACATTAAACAAGGCAGGCTTCAT	ATGAAGCCTGCCTTGTTTAATGTGGCGTGTGAAATCAAAGAAAAAACAGTT
PBX1 I84A	GGATACTCAAAACTGTTTTTTCTTTGGCTTCACACAACACATTAAACAAGGCAG	CTGCCTTGTTTAATGTGTTGTGTGAAGCCAAAGAAAAAACAGTTTTGAGTATCC
PBX1 L105A	GTCCAGCCGCATCGCCTGGGGGTCTGTG	CACAGACCCCCAGGCGATGCGGCTGGAC
PBX1 L108A	AACAGCATGTTGTCCGCCCGCATCAGCTGGGG	CCCCAGCTGATGCGGGCGGACAACATGCTGTT
PBX1 L112A	ACGCCTTCCGCTAACGCCATGTTGTCCAGCCG	CGGCTGGACAACATGGCGTTAGCGGAAGGCGT
PBX1 I197A	GCTGACCATCCGCTCAGCCTCCTTTGGGGAGATG	CATCTCCCCAAAGGAGGCTGAGCGGATGGTCAGC
PBX1 I204A	GCTGAACTTGCGGTGGGCGATGCTGACCATCCGC	GCGGATGGTCAGCATCGCCCACCGCAAGTTCAGC
PBX1 I211A	CTTGAGCTGCATCTGGGCGGAGCTGAACTTGCGG	CCGCAAGTTCAGCTCCGCCCAGATGCAGCTCAAG
PBX1Δ 43-55	CGGGAGGAAGCAGGACCAGAGTTTGGATGAGG	CCTCATCCAAACTCTGGTCCTGCTTCCTCCCG
PBX1Δ77-84	GCCACAGAATGAAGCCTGCCAAAGAAAAAACAGTTTTGAGT	ACTCAAAACTGTTTTTTCTTTGGCAGGCTTCATTCTGTGGC
PBX1 Δ105-113	CCACGCCTTCCGCCTGGGGGTCTGTG	CACAGACCCCCAGGCGGAAGGCGTGG

PBX1 Δ 197-204	AGCTGAACTTGCGGTGCTCCTTTGGGGAGATG	CATCTCCCCAAAGGAGCACCGCAAGTTCAGCT

Supplementary Table S3: List of primers used for cloning PREP1-GFP into retroviral vector pBABE

**Primer name**	**Sequence 5'-3'**
PREP1FX	ATCTCGAGGCCACCATGATGGCTACACAGACA
PREP1RK	CGGGTACCATCTGCAGGGAGTCACTGT
PREP1SnF	CGTACGTAGCCACCATGATGGCTACA
GFPSaR	GCGTCGACTTACTTGTACAGCTCGTCCA
GFPSnF	CGTACGTAATGGTGAGCAAGGGCGA

Supplementary Table S4: PREP1-PBX1 cross-linked peptides Experiment #1

Protein	Title	Spectrum Number	Precursor Mass	Peptide	Peptide Mass	Modifications	Evalue	Score	Precursor Mass Error
			(Da)		(Da)			pLINK	(ppm)
		Charge							
sp|P40424|PBX1_HUMAN (87)-sp|P40424|PBX1_HUMAN (195)		26							
	CB_4a_GluC.9754.9754.5.0.dta	5	2279,343935	KTVLSIR(1)-TRPISPKEIER(7)	2279,344428	null	8,80E-10	1,29E-13	-0,21629
	CB_4a_GluC_bis.10057.10057.5.0.dta	5	2279,345545	KTVLSIR(1)-TRPISPKEIER(7)	2279,344428	null	3,11E-10	2,40E-10	0,490053
	CB_4a_GluC_bis.10103.10103.4.0.dta	4	2279,345922	KTVLSIR(1)-TRPISPKEIER(7)	2279,344428	null	9,04E-12	5,64E-10	0,655452
	CB_4a_GluC_bis.10136.10136.5.0.dta	5	2279,345446	KTVLSIR(1)-TRPISPKEIER(7)	2279,344428	null	2,72E-09	1,49E-09	0,44662
	CB_4a_GluC.9742.9742.4.0.dta	4	2279,345322	KTVLSIR(1)-TRPISPKEIER(7)	2279,344428	null	2,04E-12	9,28E-08	0,392218
	CB_4a_GluC_bis.10028.10028.4.0.dta	4	2279,345963	KTVLSIR(1)-TRPISPKEIER(7)	2279,344428	null	9,66E-14	1,27E-07	0,673439
	CB_4a_GluC.9830.9830.4.0.dta	4	2279,345502	KTVLSIR(1)-TRPISPKEIER(7)	2279,344428	null	1,75E-09	1,99E-06	0,471188
	CB_4a_GluC_bis.10197.10197.4.0.dta	4	2279,346156	KTVLSIR(1)-TRPISPKEIER(7)	2279,344428	null	1,82E-07	9,40E-06	0,758113
	CB_4a_GluC_bis.10059.10059.3.0.dta	3	2279,346376	KTVLSIR(1)-TRPISPKEIER(7)	2279,344428	null	2,11E-10	3,52E-04	0,854632
	CB_4a_GluC_bis.9696.9696.3.0.dta	3	1881,122483	KTVLSIR(1)-TRPISPKE(7)	1881,116681	null	2,24E-25	2,56E-13	3,084338
	CB_4a_GluC.9403.9403.3.0.dta	3	1881,12337	KTVLSIR(1)-TRPISPKE(7)	1881,116681	null	6,36E-18	7,08E-12	3,555867
	CB_4a_GluC.9477.9477.4.0.dta	4	1881,123865	KTVLSIR(1)-TRPISPKE(7)	1881,116681	null	1,04E-07	3,62E-10	3,819008
	CB_4a_GluC_bis.9767.9767.4.0.dta	4	1881,122834	KTVLSIR(1)-TRPISPKE(7)	1881,116681	null	2,72E-09	5,97E-10	3,270929
	CB_4a_GluC.9402.9402.4.0.dta	4	1881,12378	KTVLSIR(1)-TRPISPKE(7)	1881,116681	null	1,09E-12	8,91E-10	3,773822
	CB_4a_GluC_bis.9693.9693.4.0.dta	4	1881,122814	KTVLSIR(1)-TRPISPKE(7)	1881,116681	null	2,81E-12	2,19E-09	3,260297
	CB_4a_GluC_bis.9768.9768.3.0.dta	3	1881,122554	KTVLSIR(1)-TRPISPKE(7)	1881,116681	null	8,73E-15	9,02E-09	3,122082
	CB_4a_GluC_bis.9761.9761.4.0.dta	4	1881,122719	KTVLSIR(1)-TRPISPKE(7)	1881,116681	null	6,47E-02	4,63E-02	3,209796
	CB_4a_GluC_bis.9679.9679.4.1.dta	4	1881,123027	KTVLSIR(1)-TRPISPKE(7)	1881,116681	null	8,25E-02	2,06E-01	3,373528
	CB_4a_GluC.10439.10439.3.0.dta	3	1623,972658	KTVLSIR(1)-PISPKE(5)	1623,967905	null	2,58E-12	1,21E-12	2,926782
	CB_4a_GluC_bis.10770.10770.3.0.dta	3	1623,972863	KTVLSIR(1)-PISPKE(5)	1623,967905	null	4,00E-14	2,31E-10	3,053016
	CB_4a_GluC_bis.10893.10893.4.0.dta	4	2022,201093	KTVLSIR(1)-PISPKEIER(5)	2022,195653	null	2,49E-08	1,54E-09	2,690145
	CB_4a_GluC.10538.10538.4.0.dta	4	2022,201371	KTVLSIR(1)-PISPKEIER(5)	2022,195653	null	9,33E-09	1,57E-08	2,82762
	CB_4a_GluC.10816.10816.3.0.dta	3	2123,244962	KTVLSIR(1)-TRPISPKEIE(7)	2123,243326	null	5,14E-10	1,27E-08	0,770519
	CB_4a_GluC_bis.11167.11167.3.0.dta	3	2123,246189	KTVLSIR(1)-TRPISPKEIE(7)	2123,243326	null	1,80E-11	2,38E-06	1,348409
	CB_4a_GluC_bis.11151.11151.4.0.dta	4	2123,243463	KTVLSIR(1)-TRPISPKEIE(7)	2123,243326	null	4,32E-07	5,24E-06	0,064524
	CB_4a_GluC_bis.11212.11212.4.4.dta	4	2123,243416	KTVLSIR(1)-TRPISPKEIE(7)	2123,243326	null	9,03E-07	4,01E-02	0,042388
sp|P40424|PBX1_HUMAN (87)-sp|P55347-2|PKNX1_HUMAN (55)		3							
	CB_4a_GluC_bis.11198.11198.4.1.dta	4	2621,393213	KTVLSIR(1)-SQTPMDVDKQAIYR(9)	2621,396588	Oxidation[M](5)	1,90E-21	7,04E-13	-1,287482
	CB_4a_GluC.10847.10847.4.2.dta	4	2621,394558	KTVLSIR(1)-SQTPMDVDKQAIYR(9)	2621,396588	Oxidation[M](5)	1,84E-20	1,73E-10	-0,774396
	CB_4a_GluC_bis.9730.9730.4.0.dta	4	1732,051638	KTVLSIR(1)-KQAIYR(1)	1732,047881	null	9,61E-14	2,75E-11	2,169109
sp|P40424|PBX1_HUMAN (153)-sp|P40424|PBX1_HUMAN (242)		5							
	CB_4a_GluC_bis.9353.9353.3.0.dta	3	1904,025457	AKLSQIR(2)-NFNKQATE(4)	1904,023512	null	2,03E-08	1,42E-07	1,021521
	CB_4a_GluC.9111.9111.3.0.dta	3	1904,026698	AKLSQIR(2)-NFNKQATE(4)	1904,023512	null	4,27E-08	7,03E-06	1,673299
	CB_4a_GluC_bis.8166.8166.3.0.dta	3	2060,131075	AKLSQIR(2)-RNFNKQATE(5)	2060,124614	null	1,42E-11	7,17E-05	3,136218
	CB_4a_GluC.7938.7938.3.0.dta	3	2060,131447	AKLSQIR(2)-RNFNKQATE(5)	2060,124614	null	2,97E-11	7,94E-05	3,31679
	CB_4a_GluC_bis.8161.8161.4.0.dta	4	2060,127337	AKLSQIR(2)-RNFNKQATE(5)	2060,124614	null	3,60E-07	1,05E-04	1,321765
sp|P40424|PBX1_HUMAN (195)-sp|P40424|PBX1_HUMAN (308)		3							
	CB_4a_GluC.9054.9054.3.0.dta	3	1815,003442	TRPISPKE(7)-ANIYAAK(7)	1815,00099	null	1,42E-13	3,24E-06	1,350963
	CB_4a_GluC_bis.9713.9713.3.0.dta	3	2213,235603	TRPISPKEIER(7)-ANIYAAK(7)	2213,228738	null	2,06E-13	4,87E-04	3,101803
	CB_4a_GluC.9418.9418.3.0.dta	3	2213,237988	TRPISPKEIER(7)-ANIYAAK(7)	2213,228738	null	2,99E-13	1,07E-02	4,179414
sp|P40424|PBX1_HUMAN (153)-sp|P55347-2|PKNX1_HUMAN (268)		6							
	CB_4a_GluC.9116.9116.5.0.dta	5	2275,308994	AKLSQIR(2)-GVLPKHATNVMR(5)	2275,306604	null	2,59E-14	8,99E-12	1,050408
	CB_4a_GluC.9098.9098.4.0.dta	4	2275,30977	AKLSQIR(2)-GVLPKHATNVMR(5)	2275,306604	null	1,80E-09	5,47E-07	1,391461
	CB_4a_GluC_bis.9247.9247.4.0.dta	4	2275,311392	AKLSQIR(2)-GVLPKHATNVMR(5)	2275,306604	null	2,21E-12	1,07E-06	2,104332
	CB_4a_GluC.8127.8127.4.0.dta	4	2291,304888	AKLSQIR(2)-GVLPKHATNVMR(5)	2291,301518	Oxidation[M](11)	4,63E-05	9,68E-06	1,47078
	CB_4a_GluC_bis.9248.9248.5.0.dta	5	2275,310613	AKLSQIR(2)-GVLPKHATNVMR(5)	2275,306604	null	2,98E-07	1,77E-05	1,76196
	CB_4a_GluC_bis.8304.8304.4.0.dta	4	2291,300449	AKLSQIR(2)-GVLPKHATNVMR(5)	2291,301518	Oxidation[M](11)	1,22E-05	7,53E-05	-0,466547
sp|P40424|PBX1_HUMAN (153)-sp|P40424|PBX1_HUMAN (308)		1							
	CB_4a_GluC_bis.10187.10187.3.0.dta	3	1702,989814	AKLSQIR(2)-ANIYAAK(7)	1702,984949	null	3,38E-18	5,98E-11	2,856749
sp|P40424|PBX1_HUMAN (153)-sp|P55347-2|PKNX1_HUMAN (153)		5							
	CB_4a_GluC.7888.7888.2.0.dta	2	1661,894684	AKLSQIR(2)-TKMNSE(2)	1661,889002	null	2,27E-05	6,71E-11	3,419001
	CB_4a_GluC.7867.7867.3.0.dta	3	1661,891897	AKLSQIR(2)-TKMNSE(2)	1661,889002	null	5,46E-11	1,01E-09	1,741994
	CB_4a_GluC_bis.8111.8111.3.0.dta	3	1661,890569	AKLSQIR(2)-TKMNSE(2)	1661,889002	null	7,08E-12	4,24E-08	0,942903
	CB_4a_GluC.6717.6717.3.0.dta	3	1677,887834	AKLSQIR(2)-TKMNSE(2)	1677,883916	Oxidation[M](13)	3,70E-08	9,69E-08	2,335084
	CB_4a_GluC_bis.6937.6937.3.0.dta	3	1677,888735	AKLSQIR(2)-TKMNSE(2)	1677,883916	Oxidation[M](13)	1,09E-08	1,80E-05	2,87207
sp|P40424|PBX1_HUMAN (308)-sp|P55347-2|PKNX1_HUMAN (268)		4							
	CB_4a_GluC.9835.9835.4.1.dta	4	2210,21193	ANIYAAK(7)-GVLPKHATNVMR(5)	2210,211314	null	7,09E-14	1,19E-10	0,278706
	CB_4a_GluC_bis.10205.10205.3.0.dta	3	2210,215623	ANIYAAK(7)-GVLPKHATNVMR(5)	2210,211314	null	1,68E-20	2,52E-10	1,949587
	CB_4a_GluC_bis.10196.10196.4.0.dta	4	2210,217331	ANIYAAK(7)-GVLPKHATNVMR(5)	2210,211314	null	2,66E-14	2,12E-08	2,722364
	CB_4a_GluC_bis.9187.9187.4.1.dta	4	2226,213222	ANIYAAK(7)-GVLPKHATNVMR(5)	2226,206228	Oxidation[M](11)	4,08E-13	3,72E-05	3,141668
sp|P40424|PBX1_HUMAN (242)-sp|P40424|PBX1_HUMAN (308)		2							
	CB_4a_GluC_bis.9103.9103.3.0.dta	3	1995,031981	RNFNKQATE(5)-ANIYAAK(7)	1995,029323	null	1,93E-15	1,95E-06	1,332311
	CB_4a_GluC.8843.8843.3.0.dta	3	1995,034195	RNFNKQATE(5)-ANIYAAK(7)	1995,029323	null	2,17E-13	2,00E-06	2,442069
sp|P55347-2|PKNX1_HUMAN (134)-sp|P55347-2|PKNX1_HUMAN (268)		1							
	CB_4a_GluC_bis.9395.9395.4.0.dta	4	2191,192764	LEKVNE(3)-GVLPKHATNVMR(5)	2191,190244	null	1,81E-05	4,72E-04	1,15006

Table legend

Protein	Accession number of the proteins involved in cross linking; the site position of the xl-Lys on the protein is reported in brackets
Title	File name .dat (and corresponding .tif) of the ms2 annotated spectrum (as reported in the repositoty)
Spectrum Number	Frequency of the ms2 spectra
Charge	Charge of the fragmented ion
Precursor Mass	Observed mass from the precursor ion (Da)
Peptide	Sequence of cross-linked peptide pair, the site position of the Lys involved in cross-linking is reported in brackets
Peptide Mass	Theoretical mass of cross-linked peptide (Da)
Modifications	Cys Carbamidomethyl or Met Oxidation
Evalue	
Score	pLink score
Precursor Mass Error	precursor mass error in ppm

Criteria applied for manual evaluation of spectra confidently identified = E-value < 0,001 (corresponding to FDR < 0,05); the majority of the ions of the spectrum are assigned and 4 consecutive b or y ions for each xl peptide pair observed

Supplementary Table S5: PREP1-PBX1 looped peptides Experiment #1

Protein	Title	Spectrum Number	Precursor Mass	Peptide	Peptide Mass	Modifications	Evalue	Score	Precursor Mass Error
			(Da)		(Da)			pLINK	(ppm)
		Charge							
sp|P40424|PBX1_HUMAN (292)(293)		7							
	CB_4a_GluC.10473.10473.2.0.dta	2	1521,797828	YKKNIGKFQEE(2)(3)	1521,794702	null	3,66E-50	2,10E-02	2,054154
	CB_4a_GluC.7545.7545.3.0.dta	3	1257,7674	IRYKKNIGK(4)(5)	1257,767706	null	3,55E-15	3,40E-02	-0,243288
	CB_4a_GluC.7564.7564.2.0.dta	2	1257,768681	IRYKKNIGK(4)(5)	1257,767706	null	9,76E-28	3,98E-02	0,775183
	CB_4a_GluC.7255.7255.2.0.dta	2	988,584147	YKKNIGK(2)(3)	988,582546	null	9,15E-14	3,48E-02	1,61949
	CB_4a_GluC.7178.7178.2.0.dta	2	988,584185	YKKNIGK(2)(3)	988,582546	null	2,68E-19	4,00E-02	1,657929
	CB_4a_GluC_bis.7423.7423.2.0.dta	2	988,583137	YKKNIGK(2)(3)	988,582546	null	4,14E-26	7,56E-02	0,597826
	CB_4a_GluC.7173.7173.2.0.dta	2	988,584425	YKKNIGK(2)(3)	988,582546	null	2,07E-18	8,70E-02	1,900701
sp|P55347-2|PKNX1_HUMAN (334)(335)		4							
	CB_4a_GluC.3713.3713.2.0.dta	2	1463,844217	KKTAQNRPVQR(1)(2)	1463,844048	null	1,90E-16	5,05E-02	0,115449
	CB_4a_GluC.3698.3698.3.0.dta	3	1463,844439	KKTAQNRPVQR(1)(2)	1463,844048	null	2,49E-26	6,40E-02	0,267105
	CB_4a_GluC.3372.3372.2.0.dta	2	983,565388	KKTAQNR(1)(2)	983,563206	null	1,21E-30	5,90E-02	2,218464
	CB_4a_GluC_bis.3462.3462.2.0.dta	2	983,565925	KKTAQNR(1)(2)	983,563206	null	2,59E-23	1,16E-01	2,764439
sp|P55347-2|PKNX1_HUMAN (331)(333)		2							
	CB_4a_GluC.4661.4661.2.0.dta	2	1390,689554	SSCSETPKTKK(8)(10)	1390,688191	Carbamidomethyl[C](3)	2,04E-26	3,10E-02	0,98009
	CB_4a_GluC_bis.4856.4856.2.0.dta	2	1390,688445	SSCSETPKTKK(8)(10)	1390,688191	Carbamidomethyl[C](3)	2,27E-44	7,11E-02	0,182643
sp|P55347-2|PKNX1_HUMAN (260)(262)		5							
	CB_4a_GluC.7682.7682.4.0.dta	4	1835,962896	LSILHQDDGSSKNKR(12)(14)	1835,960913	null	3,46E-28	4,87E-02	1,080088
	CB_4a_GluC.8535.8535.3.0.dta	3	1835,968132	LSILHQDDGSSKNKR(12)(14)	1835,960913	null	4,19E-32	6,15E-02	3,932001
	CB_4a_GluC_bis.8825.8825.3.0.dta	3	1835,967448	LSILHQDDGSSKNKR(12)(14)	1835,960913	null	1,28E-65	6,90E-02	3,559444
	CB_4a_GluC_bis.8904.8904.3.0.dta	3	1835,967345	LSILHQDDGSSKNKR(12)(14)	1835,960913	null	3,93E-33	9,89E-02	3,503343
	CB_4a_GluC_bis.9017.9017.3.0.dta	3	1835,960322	LSILHQDDGSSKNKR(12)(14)	1835,960913	null	4,63E-24	1,25E-01	-0,321902

Table legend

Protein	Accession number of the proteins involved in cross linking; the site position of the xl-Lys on the protein is reported in brackets
Title	File name .dat (and corresponding .tif) of the ms2 annotated spectrum (as reported in the repositoty)
Spectrum Number	Frequency of the ms2 spectra
Charge	Charge of the fragmented ion
Precursor Mass	Observed mass from the precursor ion (Da)
Peptide	Sequence of cross-linked peptide pair, the site position of the Lys involved in cross-linking is reported in brackets
Peptide Mass	Theoretical mass of cross-linked peptide (Da)
Modifications	Cys Carbamidomethyl or Met Oxidation
Evalue	
Score	pLink score
Precursor Mass Error	precursor mass error in ppm

Criteria applied for manual evaluation of spectra confidently identified = E-value < 0,001 (corresponding to FDR < 0,05); the majority of the ions of the spectrum are assigned and 4 consecutive b or y ions for each xl peptide pair observed

Supplementary Table S6: PREP1-PBX1 cross-linked peptides Experiment #2

<table>
<tr><th>Protein</th><th>Title</th><th>Spectrum Number</th><th>Precursor Mass</th><th>Peptide</th><th>Peptide Mass</th><th>Modifications</th><th>Evalue</th><th>Score</th><th>Precursor Mass Error</th></tr>
<tr><td></td><td></td><td></td><td>(Da)</td><td></td><td>(Da)</td><td></td><td></td><td>pLINK</td><td>(ppm)</td></tr>
<tr><td></td><td></td><td>Charge</td><td></td><td></td><td></td><td></td><td></td><td></td><td></td></tr>
<tr><td>sp|P40424|PBX1_HUMAN (87)-sp|P40424|PBX1_HUMAN (195)</td><td></td><td>19</td><td></td><td></td><td></td><td></td><td></td><td></td><td></td></tr>
<tr><td></td><td>CB_FB_180416_Prep1_Tryp_GluC_3.11563.11563.5.0.dta</td><td>5</td><td>2279,359429</td><td>KTVLSIR(1)-TRPISPKEIER(7)</td><td>2279,344428</td><td>null</td><td>8,55E-16</td><td>1,85E-13</td><td>6,581278</td></tr>
<tr><td></td><td>CB_FB_180416_Prep1_Tryp_GluC_3.11664.11664.5.0.dta</td><td>5</td><td>2279,358459</td><td>KTVLSIR(1)-TRPISPKEIER(7)</td><td>2279,344428</td><td>null</td><td>9,13E-10</td><td>1,20E-10</td><td>6,155717</td></tr>
<tr><td></td><td>CB_FB_180416_Prep1_Tryp_GluC_3.11478.11478.5.0.dta</td><td>5</td><td>2279,359209</td><td>KTVLSIR(1)-TRPISPKEIER(7)</td><td>2279,344428</td><td>null</td><td>1,21E-12</td><td>8,71E-10</td><td>6,484759</td></tr>
<tr><td></td><td>CB_FB_180416_Prep1_Tryp_GluC_3.11562.11562.4.0.dta</td><td>4</td><td>2279,360169</td><td>KTVLSIR(1)-TRPISPKEIER(7)</td><td>2279,344428</td><td>null</td><td>3,95E-13</td><td>9,19E-09</td><td>6,905933</td></tr>
<tr><td></td><td>CB_FB_180416_Prep1_Tryp_GluC_3.11659.11659.4.0.dta</td><td>4</td><td>2279,357757</td><td>KTVLSIR(1)-TRPISPKEIER(7)</td><td>2279,344428</td><td>null</td><td>6,42E-10</td><td>1,10E-08</td><td>5,847734</td></tr>
<tr><td></td><td>CB_FB_180416_Prep1_Tryp_GluC_3.11758.11758.4.0.dta</td><td>4</td><td>2279,356112</td><td>KTVLSIR(1)-TRPISPKEIER(7)</td><td>2279,344428</td><td>null</td><td>1,48E-09</td><td>1,51E-07</td><td>5,126035</td></tr>
<tr><td></td><td>CB_FB_180416_Prep1_Tryp_GluC_3.11469.11469.4.0.dta</td><td>4</td><td>2279,360279</td><td>KTVLSIR(1)-TRPISPKEIER(7)</td><td>2279,344428</td><td>null</td><td>2,97E-11</td><td>1,73E-07</td><td>6,954193</td></tr>
<tr><td></td><td>CB_FB_180416_Prep1_Tryp_GluC_3.12296.12296.4.0.dta</td><td>4</td><td>2279,357191</td><td>KTVLSIR(1)-TRPISPKEIER(7)</td><td>2279,344428</td><td>null</td><td>8,73E-13</td><td>3,78E-07</td><td>5,599417</td></tr>
<tr><td></td><td>CB_FB_180416_Prep1_Tryp_GluC_3.11572.11572.4.0.dta</td><td>4</td><td>2279,360169</td><td>KTVLSIR(1)-TRPISPKEIER(7)</td><td>2279,344428</td><td>null</td><td>1,14E-06</td><td>2,64E-04</td><td>6,905933</td></tr>
<tr><td></td><td>CB_FB_180416_Prep1_Tryp_GluC_3.11476.11476.3.0.dta</td><td>3</td><td>2279,358399</td><td>KTVLSIR(1)-TRPISPKEIER(7)</td><td>2279,344428</td><td>null</td><td>1,94E-17</td><td>2,04E-01</td><td>6,129394</td></tr>
<tr><td></td><td>CB_FB_180416_Prep1_Tryp_GluC_3.12723.12723.4.0.dta</td><td>4</td><td>2022,200532</td><td>KTVLSIR(1)-PISPKEIER(5)</td><td>2022,195653</td><td>null</td><td>1,79E-09</td><td>2,71E-13</td><td>2,412724</td></tr>
<tr><td></td><td>CB_FB_180416_Prep1_Tryp_GluC_3.12418.12418.3.0.dta</td><td>3</td><td>1623,97707</td><td>KTVLSIR(1)-PISPKE(5)</td><td>1623,967905</td><td>null</td><td>8,36E-09</td><td>4,14E-13</td><td>5,643584</td></tr>
<tr><td></td><td>CB_FB_180416_Prep1_Tryp_GluC_3.11070.11070.3.0.dta</td><td>3</td><td>1881,125879</td><td>KTVLSIR(1)-TRPISPKE(7)</td><td>1881,116681</td><td>null</td><td>3,88E-13</td><td>1,41E-11</td><td>4,889649</td></tr>
<tr><td></td><td>CB_FB_180416_Prep1_Tryp_GluC_3.11132.11132.4.0.dta</td><td>4</td><td>1881,126678</td><td>KTVLSIR(1)-TRPISPKE(7)</td><td>1881,116681</td><td>null</td><td>6,42E-10</td><td>8,72E-11</td><td>5,314397</td></tr>
<tr><td></td><td>CB_FB_180416_Prep1_Tryp_GluC_3.12893.12893.3.0.dta</td><td>3</td><td>2123,256433</td><td>KTVLSIR(1)-TRPISPKEIE(7)</td><td>2123,243326</td><td>null</td><td>1,05E-13</td><td>5,35E-07</td><td>6,173103</td></tr>
<tr><td></td><td>CB_FB_180416_Prep1_Tryp_GluC_3.12987.12987.3.0.dta</td><td>3</td><td>2123,257251</td><td>KTVLSIR(1)-TRPISPKEIE(7)</td><td>2123,243326</td><td>null</td><td>1,70E-10</td><td>6,78E-06</td><td>6,558363</td></tr>
<tr><td></td><td>CB_FB_180416_Prep1_Tryp_GluC_3.12892.12892.4.0.dta</td><td>4</td><td>2123,256962</td><td>KTVLSIR(1)-TRPISPKEIE(7)</td><td>2123,243326</td><td>null</td><td>7,76E-09</td><td>1,42E-05</td><td>6,42225</td></tr>
<tr><td></td><td>CB_FB_180416_Prep1_Tryp_GluC_3.12986.12986.4.0.dta</td><td>4</td><td>2123,257839</td><td>KTVLSIR(1)-TRPISPKEIE(7)</td><td>2123,243326</td><td>null</td><td>5,07E-06</td><td>2,36E-04</td><td>6,835298</td></tr>
<tr><td></td><td>CB_FB_180416_Prep1_Tryp_GluC_3.11976.11976.4.1.dta</td><td>4</td><td>2408,401589</td><td>EKTVLSIR(2)-TRPISPKEIER(7)</td><td>2408,387016</td><td>null</td><td>1,28E-11</td><td>2,34E-04</td><td>6,050938</td></tr>
<tr><td>sp|P40424|PBX1_HUMAN (87)-sp|P55347-2|PKNX1_HUMAN (55)</td><td></td><td>16</td><td></td><td></td><td></td><td></td><td></td><td></td><td></td></tr>
<tr><td></td><td>CB_FB_180416_Prep1_Tryp_GluC_3.11125.11125.3.0.dta</td><td>3</td><td>1732,056322</td><td>KTVLSIR(1)-KQAIYR(1)</td><td>1732,047881</td><td>null</td><td>2,74E-15</td><td>7,28E-14</td><td>4,873422</td></tr>
<tr><td></td><td>CB_FB_180416_Prep1_Tryp_GluC_3.11118.11118.4.0.dta</td><td>4</td><td>1732,05599</td><td>KTVLSIR(1)-KQAIYR(1)</td><td>1732,047881</td><td>null</td><td>1,64E-12</td><td>2,32E-13</td><td>4,681741</td></tr>
<tr><td></td><td>CB_FB_180416_Prep1_Tryp_GluC_3.13027.13027.4.0.dta</td><td>4</td><td>2621,414281</td><td>KTVLSIR(1)-SQTPMDVDKQAIYR(9)</td><td>2621,396588</td><td>Oxidation[M](5)</td><td>6,85E-27</td><td>5,08E-13</td><td>6,749456</td></tr>
<tr><td></td><td>CB_FB_180416_Prep1_Tryp_GluC_3.13130.13130.4.0.dta</td><td>4</td><td>2621,413312</td><td>KTVLSIR(1)-SQTPMDVDKQAIYR(9)</td><td>2621,396588</td><td>Oxidation[M](5)</td><td>1,26E-21</td><td>2,37E-10</td><td>6,379805</td></tr>
<tr><td></td><td>CB_FB_180416_Prep1_Tryp_GluC_3.13052.13052.3.0.dta</td><td>3</td><td>2621,413207</td><td>KTVLSIR(1)-SQTPMDVDKQAIYR(9)</td><td>2621,396588</td><td>Oxidation[M](5)</td><td>1,73E-16</td><td>9,91E-10</td><td>6,33975</td></tr>
<tr><td></td><td>CB_FB_180416_Prep1_Tryp_GluC_3.14385.14385.4.0.dta</td><td>4</td><td>2605,41678</td><td>KTVLSIR(1)-SQTPMDVDKQAIYR(9)</td><td>2605,401674</td><td>null</td><td>3,34E-19</td><td>5,45E-08</td><td>5,797954</td></tr>
<tr><td></td><td>CB_FB_180416_Prep1_Tryp_GluC_3.13152.13152.3.0.dta</td><td>3</td><td>2621,411985</td><td>KTVLSIR(1)-SQTPMDVDKQAIYR(9)</td><td>2621,396588</td><td>Oxidation[M](5)</td><td>2,63E-12</td><td>2,21E-01</td><td>5,873587</td></tr>
<tr><td></td><td>CB_FB_180416_Prep1_Tryp_GluC_3.14202.14202.4.0.dta</td><td>4</td><td>3383,816763</td><td>KTVLSIR(1)-GVSPPPVESQTPMDVDKQAIYR(17)</td><td>3383,787757</td><td>Oxidation[M](13)</td><td>2,73E-18</td><td>6,33E-08</td><td>8,572051</td></tr>
<tr><td></td><td>CB_FB_180416_Prep1_Tryp_GluC_3.14099.14099.4.0.dta</td><td>4</td><td>3383,811298</td><td>KTVLSIR(1)-GVSPPPVESQTPMDVDKQAIYR(17)</td><td>3383,787757</td><td>Oxidation[M](13)</td><td>1,16E-23</td><td>2,83E-07</td><td>6,956997</td></tr>
<tr><td></td><td>CB_FB_180416_Prep1_Tryp_GluC_3.14298.14298.4.0.dta</td><td>4</td><td>3383,818207</td><td>KTVLSIR(1)-GVSPPPVESQTPMDVDKQAIYR(17)</td><td>3383,787757</td><td>Oxidation[M](13)</td><td>3,01E-16</td><td>9,27E-07</td><td>8,998791</td></tr>
<tr><td></td><td>CB_FB_180416_Prep1_Tryp_GluC_3.14395.14395.4.0.dta</td><td>4</td><td>3383,809634</td><td>KTVLSIR(1)-GVSPPPVESQTPMDVDKQAIYR(17)</td><td>3383,787757</td><td>Oxidation[M](13)</td><td>6,11E-11</td><td>1,12E-06</td><td>6,46524</td></tr>
<tr><td></td><td>CB_FB_180416_Prep1_Tryp_GluC_3.14207.14207.5.0.dta</td><td>5</td><td>3383,812066</td><td>KTVLSIR(1)-GVSPPPVESQTPMDVDKQAIYR(17)</td><td>3383,787757</td><td>Oxidation[M](13)</td><td>4,39E-15</td><td>7,49E-06</td><td>7,183961</td></tr>
<tr><td></td><td>CB_FB_180416_Prep1_Tryp_GluC_3.15396.15396.5.0.dta</td><td>5</td><td>3367,819835</td><td>KTVLSIR(1)-GVSPPPVESQTPMDVDKQAIYR(17)</td><td>3367,792843</td><td>null</td><td>7,06E-20</td><td>1,11E-05</td><td>8,014745</td></tr>
<tr><td></td><td>CB_FB_180416_Prep1_Tryp_GluC_3.15313.15313.4.0.dta</td><td>4</td><td>3367,820597</td><td>KTVLSIR(1)-GVSPPPVESQTPMDVDKQAIYR(17)</td><td>3367,792843</td><td>null</td><td>3,12E-21</td><td>4,33E-04</td><td>8,241006</td></tr>
<tr><td></td><td>CB_FB_180416_Prep1_Tryp_GluC_3.14497.14497.4.4.dta</td><td>4</td><td>3383,823445</td><td>KTVLSIR(1)-GVSPPPVESQTPMDVDKQAIYR(17)</td><td>3383,787757</td><td>Oxidation[M](13)</td><td>1,79E-22</td><td>8,11E-04</td><td>10,546761</td></tr>
<tr><td></td><td>CB_FB_180416_Prep1_Tryp_GluC_3.15386.15386.4.0.dta</td><td>4</td><td>3367,821689</td><td>KTVLSIR(1)-GVSPPPVESQTPMDVDKQAIYR(17)</td><td>3367,792843</td><td>null</td><td>2,78E-16</td><td>2,69E-03</td><td>8,565254</td></tr>
<tr><td>sp|P40424|PBX1_HUMAN (242)-sp|P40424|PBX1_HUMAN (308)</td><td></td><td>3</td><td></td><td></td><td></td><td></td><td></td><td></td><td></td></tr>
<tr><td></td><td>CB_FB_180416_Prep1_Tryp_GluC_3.12344.12344.2.0.dta</td><td>2</td><td>1967,983303</td><td>NFNKQATE(4)-EANIYAAK(8)</td><td>1967,970809</td><td>null</td><td>3,35E-08</td><td>1,27E-07</td><td>6,348671</td></tr>
<tr><td></td><td>CB_FB_180416_Prep1_Tryp_GluC_3.10255.10255.2.0.dta</td><td>2</td><td>1995,03927</td><td>RNFNKQATE(5)-ANIYAAK(7)</td><td>1995,029323</td><td>null</td><td>4,74E-10</td><td>1,58E-06</td><td>4,985892</td></tr>
<tr><td></td><td>CB_FB_180416_Prep1_Tryp_GluC_3.11996.11996.2.0.dta</td><td>2</td><td>1838,940423</td><td>NFNKQATE(4)-ANIYAAK(7)</td><td>1838,928221</td><td>null</td><td>2,06E-05</td><td>9,65E-05</td><td>6,635387</td></tr>
<tr><td>sp|P40424|PBX1_HUMAN (65)-sp|P40424|PBX1_HUMAN (195)</td><td></td><td>6</td><td></td><td></td><td></td><td></td><td></td><td></td><td></td></tr>
<tr><td></td><td>CB_FB_180416_Prep1_Tryp_GluC_3.6884.6884.5.0.dta</td><td>5</td><td>2498,35256</td><td>KHALNCHR(1)-TRPISPKEIER(7)</td><td>2498,34075</td><td>Carbamidomethyl[C](20)</td><td>7,54E-15</td><td>6,75E-15</td><td>4,727137</td></tr>
<tr><td></td><td>CB_FB_180416_Prep1_Tryp_GluC_3.6794.6794.5.0.dta</td><td>5</td><td>2498,351333</td><td>KHALNCHR(1)-TRPISPKEIER(7)</td><td>2498,34075</td><td>Carbamidomethyl[C](20)</td><td>1,89E-12</td><td>2,93E-13</td><td>4,236011</td></tr>
<tr><td></td><td>CB_FB_180416_Prep1_Tryp_GluC_3.5318.5318.4.0.dta</td><td>4</td><td>2100,125903</td><td>KHALNCHR(1)-TRPISPKE(7)</td><td>2100,113002</td><td>Carbamidomethyl[C](6)</td><td>1,22E-12</td><td>1,64E-10</td><td>6,143003</td></tr>
<tr><td></td><td>CB_FB_180416_Prep1_Tryp_GluC_3.5324.5324.5.0.dta</td><td>5</td><td>2100,124388</td><td>KHALNCHR(1)-TRPISPKE(7)</td><td>2100,113002</td><td>Carbamidomethyl[C](6)</td><td>2,19E-08</td><td>4,82E-09</td><td>5,421613</td></tr>
<tr><td></td><td>CB_FB_180416_Prep1_Tryp_GluC_3.5334.5334.3.0.dta</td><td>3</td><td>2100,125128</td><td>KHALNCHR(1)-TRPISPKE(7)</td><td>2100,113002</td><td>Carbamidomethyl[C](6)</td><td>1,80E-11</td><td>2,06E-04</td><td>5,773975</td></tr>
<tr><td></td><td>CB_FB_180416_Prep1_Tryp_GluC_3.7590.7590.5.0.dta</td><td>5</td><td>2342,252694</td><td>KHALNCHR(1)-TRPISPKEIE(7)</td><td>2342,239647</td><td>Carbamidomethyl[C](19)</td><td>9,97E-12</td><td>1,58E-06</td><td>5,57031</td></tr>
<tr><td>sp|P40424|PBX1_HUMAN (195)-sp|P40424|PBX1_HUMAN (308)</td><td></td><td>4</td><td></td><td></td><td></td><td></td><td></td><td></td><td></td></tr>
<tr><td></td><td>CB_FB_180416_Prep1_Tryp_GluC_3.11089.11089.4.0.dta</td><td>4</td><td>2213,236968</td><td>TRPISPKEIER(7)-ANIYAAK(7)</td><td>2213,228738</td><td>null</td><td>3,44E-13</td><td>3,21E-09</td><td>3,718549</td></tr>
<tr><td></td><td>CB_FB_180416_Prep1_Tryp_GluC_3.10503.10503.2.0.dta</td><td>2</td><td>1815,015195</td><td>TRPISPKE(7)-ANIYAAK(7)</td><td>1815,00099</td><td>null</td><td>4,42E-11</td><td>8,87E-07</td><td>7,826442</td></tr>
<tr><td></td><td>CB_FB_180416_Prep1_Tryp_GluC_3.10488.10488.3.0.dta</td><td>3</td><td>1815,014094</td><td>TRPISPKE(7)-ANIYAAK(7)</td><td>1815,00099</td><td>null</td><td>4,98E-13</td><td>3,89E-03</td><td>7,219831</td></tr>
<tr><td></td><td>CB_FB_180416_Prep1_Tryp_GluC_3.12600.12600.3.0.dta</td><td>3</td><td>2057,135549</td><td>TRPISPKEIE(7)-ANIYAAK(7)</td><td>2057,127636</td><td>null</td><td>2,92E-12</td><td>3,53E-05</td><td>3,846626</td></tr>
<tr><td>sp|P40424|PBX1_HUMAN (153)-sp|P40424|PBX1_HUMAN (308)</td><td></td><td>5</td><td></td><td></td><td></td><td></td><td></td><td></td><td></td></tr>
<tr><td></td><td>CB_FB_180416_Prep1_Tryp_GluC_3.11614.11614.2.0.dta</td><td>2</td><td>1702,9955</td><td>AKLSQIR(2)-ANIYAAK(7)</td><td>1702,984949</td><td>null</td><td>1,36E-11</td><td>4,07E-19</td><td>6,195592</td></tr>
<tr><td></td><td>CB_FB_180416_Prep1_Tryp_GluC_3.11612.11612.3.0.dta</td><td>3</td><td>1702,996352</td><td>AKLSQIR(2)-ANIYAAK(7)</td><td>1702,984949</td><td>null</td><td>1,76E-20</td><td>9,18E-11</td><td>6,69589</td></tr>
<tr><td></td><td>CB_FB_180416_Prep1_Tryp_GluC_3.11707.11707.3.0.dta</td><td>3</td><td>1702,995935</td><td>AKLSQIR(2)-ANIYAAK(7)</td><td>1702,984949</td><td>null</td><td>2,46E-15</td><td>1,43E-09</td><td>6,451026</td></tr>
<tr><td></td><td>CB_FB_180416_Prep1_Tryp_GluC_3.11922.11922.3.1.dta</td><td>3</td><td>1702,995032</td><td>AKLSQIR(2)-ANIYAAK(7)</td><td>1702,984949</td><td>null</td><td>1,91E-11</td><td>5,76E-09</td><td>5,92078</td></tr>
<tr><td></td><td>CB_FB_180416_Prep1_Tryp_GluC_3.11638.11638.4.0.dta</td><td>4</td><td>1702,99401</td><td>AKLSQIR(2)-ANIYAAK(7)</td><td>1702,984949</td><td>null</td><td>1,32E-06</td><td>1,41E-05</td><td>5,320658</td></tr>
<tr><td>sp|P40424|PBX1_HUMAN (153)-sp|P40424|PBX1_HUMAN (242)</td><td></td><td>2</td><td></td><td></td><td></td><td></td><td></td><td></td><td></td></tr>
<tr><td></td><td>CB_FB_180416_Prep1_Tryp_GluC_3.10639.10639.2.0.dta</td><td>2</td><td>1904,031906</td><td>AKLSQIR(2)-NFNKQATE(4)</td><td>1904,023512</td><td>null</td><td>7,04E-13</td><td>1,60E-17</td><td>4,408559</td></tr>
<tr><td></td><td>CB_FB_180416_Prep1_Tryp_GluC_3.8994.8994.4.0.dta</td><td>4</td><td>2060,133109</td><td>AKLSQIR(2)-RNFNKQATE(5)</td><td>2060,124614</td><td>null</td><td>2,60E-06</td><td>4,93E-04</td><td>4,123537</td></tr>
<tr><td>sp|P40424|PBX1_HUMAN (87)-sp|P40424|PBX1_HUMAN (308)</td><td></td><td>2</td><td></td><td></td><td></td><td></td><td></td><td></td><td></td></tr>
<tr><td></td><td>CB_FB_180416_Prep1_Tryp_GluC_3.13460.13460.2.0.dta</td><td>2</td><td>1704,012436</td><td>KTVLSIR(1)-ANIYAAK(7)</td><td>1704,00535</td><td>null</td><td>5,08E-13</td><td>8,15E-20</td><td>4,158438</td></tr>
<tr><td></td><td>CB_FB_180416_Prep1_Tryp_GluC_3.13455.13455.3.0.dta</td><td>3</td><td>1704,011255</td><td>KTVLSIR(1)-ANIYAAK(7)</td><td>1704,00535</td><td>null</td><td>3,11E-12</td><td>1,32E-10</td><td>3,465365</td></tr>
<tr><td>sp|P55347-2|PKNX1_HUMAN (134)-sp|P55347-2|PKNX1_HUMAN (140)</td><td></td><td>3</td><td></td><td></td><td></td><td></td><td></td><td></td><td></td></tr>
<tr><td></td><td>CB_FB_180416_Prep1_Tryp_GluC_3.11343.11343.2.0.dta</td><td>2</td><td>1953,946564</td><td>LEKVNE(3)-LCKDFCSR(3)</td><td>1953,94077</td><td>Carbamidomethyl[C](2);Carbamidomethyl[C](6)</td><td>1,32E-10</td><td>1,51E-13</td><td>2,965289</td></tr>
<tr><td></td><td>CB_FB_180416_Prep1_Tryp_GluC_3.11333.11333.3.0.dta</td><td>3</td><td>1953,948688</td><td>LEKVNE(3)-LCKDFCSR(3)</td><td>1953,94077</td><td>Carbamidomethyl[C](2);Carbamidomethyl[C](6)</td><td>3,46E-10</td><td>7,45E-09</td><td>4,052323</td></tr>
<tr><td></td><td>CB_FB_180416_Prep1_Tryp_GluC_3.11446.11446.3.0.dta</td><td>3</td><td>1953,948843</td><td>LEKVNE(3)-LCKDFCSR(3)</td><td>1953,94077</td><td>Carbamidomethyl[C](2);Carbamidomethyl[C](6)</td><td>5,08E-05</td><td>3,80E-03</td><td>4,13165</td></tr>
<tr><td>sp|P40424|PBX1_HUMAN (87)-sp|P40424|PBX1_HUMAN (153)</td><td></td><td>2</td><td></td><td></td><td></td><td></td><td></td><td></td><td></td></tr>
<tr><td></td><td>CB_FB_180416_Prep1_Tryp_GluC_3.11934.11934.4.0.dta</td><td>4</td><td>1769,113021</td><td>KTVLSIR(1)-AKLSQIR(2)</td><td>1769,10064</td><td>null</td><td>1,27E-09</td><td>1,52E-11</td><td>6,998471</td></tr>
</table>

	CB_FB_180416_Prep1_Tryp_GluC_3.11952.11952.3.0.dta	3	1769,115016	KTVLSIR(1)-AKLSQIR(2)	1769,10064	null	5,98E-09	2,06E-10	8,126163
sp|P40424|PBX1_HUMAN (308)-sp|P55347-2|PKNX1_HUMAN (134)		1							
	CB_FB_180416_Prep1_Tryp_GluC_3.11861.11861.2.0.dta	2	1618,87949	ANIYAAK(7)-LEKVNE(3)	1618,868589	null	8,79E-10	1,43E-09	6,733715
sp|P40424|PBX1_HUMAN (65)-sp|P40424|PBX1_HUMAN (308)		1							
	CB_FB_180416_Prep1_Tryp_GluC_3.7161.7161.4.0.dta	4	1923,013309	KHALNCHR(1)-ANIYAAK(7)	1923,001671	Carbamidomethyl[C](6)	2,84E-08	3,57E-09	6,051997
sp|P40424|PBX1_HUMAN (65)-sp|P40424|PBX1_HUMAN (74)		3							
	CB_FB_180416_Prep1_Tryp_GluC_3.14528.14528.3.0.dta	3	2510,260131	KHALNCHR(1)-MKPALFNVLCE(2)	2510,246384	Carbamidomethyl[C](10);Oxidation[M](1);Carbamidomethyl[C](20)	1,40E-13	1,05E-08	5,476355
	CB_FB_180416_Prep1_Tryp_GluC_3.14544.14544.5.0.dta	5	2510,25566	KHALNCHR(1)-MKPALFNVLCE(2)	2510,246384	Carbamidomethyl[C](10);Oxidation[M](1);Carbamidomethyl[C](20)	1,33E-07	3,01E-05	3,695255
	CB_FB_180416_Prep1_Tryp_GluC_3.15057.15057.3.0.dta	3	2494,267085	KHALNCHR(1)-MKPALFNVLCE(2)	2494,25147	Carbamidomethyl[C](10);Carbamidomethyl[C](20)	1,24E-10	1,76E-04	6,260395
sp|P40424|PBX1_HUMAN (65)-sp|P40424|PBX1_HUMAN (87)		1							
	CB_FB_180416_Prep1_Tryp_GluC_3.7771.7771.4.2.dta	4	1989,127062	KHALNCHR(1)-KTVLSIR(1)	1989,117362	Carbamidomethyl[C](6)	1,89E-09	1,77E-06	4,876535
sp|P40424|PBX1_HUMAN (308)-sp|P55347-2|PKNX1_HUMAN (268)		4							
	CB_FB_180416_Prep1_Tryp_GluC_3.11541.11541.3.0.dta	3	2210,227111	ANIYAAK(7)-GVLPKHATNVMR(5)	2210,211314	null	5,37E-18	1,82E-06	7,14728
	CB_FB_180416_Prep1_Tryp_GluC_3.11549.11549.4.0.dta	4	2210,226008	ANIYAAK(7)-GVLPKHATNVMR(5)	2210,211314	null	3,25E-11	7,44E-04	6,648233
	CB_FB_180416_Prep1_Tryp_GluC_3.10416.10416.4.0.dta	4	2226,221772	ANIYAAK(7)-GVLPKHATNVMR(5)	2226,206228	Oxidation[M](11)	2,67E-12	9,16E-04	6,982282
	CB_FB_180416_Prep1_Tryp_GluC_3.11626.11626.3.0.dta	3	2210,227032	ANIYAAK(7)-GVLPKHATNVMR(5)	2210,211314	null	1,75E-12	3,67E-02	7,111537
sp|P40424|PBX1_HUMAN (87)-sp|P55347-2|PKNX1_HUMAN (268)		2							
	CB_FB_180416_Prep1_Tryp_GluC_3.11800.11800.5.0.dta	5	2276,339091	KTVLSIR(1)-GVLPKHATNVMR(5)	2276,327005	null	9,81E-10	2,21E-06	5,30943
	CB_FB_180416_Prep1_Tryp_GluC_3.10849.10849.5.0.dta	5	2292,330628	KTVLSIR(1)-GVLPKHATNVMR(5)	2292,321919	Oxidation[M](11)	1,93E-04	4,63E-03	3,799205
sp|P40424|PBX1_HUMAN (87)-sp|P55347-2|PKNX1_HUMAN (73)		2							
	CB_FB_180416_Prep1_Tryp_GluC_3.9431.9431.3.0.dta	3	2107,082479	KTVLSIR(1)-KCEQSTQGSE(1)	2107,069859	Carbamidomethyl[C](2)	8,13E-15	2,26E-06	5,98936
	CB_FB_180416_Prep1_Tryp_GluC_3.9468.9468.2.0.dta	2	2107,078565	KTVLSIR(1)-KCEQSTQGSE(1)	2107,069859	Carbamidomethyl[C](2)	8,89E-11	1,37E-02	4,131804
sp|P40424|PBX1_HUMAN (308)-sp|P55347-2|PKNX1_HUMAN (140)		1							
	CB_FB_180416_Prep1_Tryp_GluC_3.12398.12398.3.0.dta	3	1972,974595	ANIYAAK(7)-LCKDFCSR(3)	1972,96184	Carbamidomethyl[C](2);Carbamidomethyl[C](6)	4,02E-14	3,87E-06	6,464899
sp|P40424|PBX1_HUMAN (153)-sp|P55347-2|PKNX1_HUMAN (268)		5							
	CB_FB_180416_Prep1_Tryp_GluC_3.10373.10373.5.0.dta	5	2275,318296	AKLSQIR(2)-GVLPKHATNVMR(5)	2275,306604	null	4,37E-08	5,39E-06	5,138648
	CB_FB_180416_Prep1_Tryp_GluC_3.10482.10482.5.0.dta	5	2275,320514	AKLSQIR(2)-GVLPKHATNVMR(5)	2275,306604	null	1,05E-08	1,26E-05	6,113462
	CB_FB_180416_Prep1_Tryp_GluC_3.9111.9111.4.0.dta	4	2291,312125	AKLSQIR(2)-GVLPKHATNVMR(5)	2291,301518	Oxidation[M](11)	6,34E-06	4,94E-02	4,629247
	CB_FB_180416_Prep1_Tryp_GluC_3.10378.10378.4.0.dta	4	2275,318867	AKLSQIR(2)-GVLPKHATNVMR(5)	2275,306604	null	1,54E-09	1,33E-01	5,389603
	CB_FB_180416_Prep1_Tryp_GluC_3.9117.9117.3.0.dta	3	2291,308453	AKLSQIR(2)-GVLPKHATNVMR(5)	2291,301518	Oxidation[M](11)	3,74E-11	1,97E-01	3,026664
sp|P55347-2|PKNX1_HUMAN (140)-sp|P55347-2|PKNX1_HUMAN (151)		2							
	CB_FB_180416_Prep1_Tryp_GluC_3.11965.11965.3.0.dta	3	2219,118045	LCKDFCSR(3)-YIACLKTK(6)	2219,102025	Carbamidomethyl[C](2);Carbamidomethyl[C](6);Carbamidomethyl[C](15)	4,45E-10	2,28E-04	7,219136
	CB_FB_180416_Prep1_Tryp_GluC_3.11951.11951.4.0.dta	4	2219,117154	LCKDFCSR(3)-YIACLKTK(6)	2219,102025	Carbamidomethyl[C](2);Carbamidomethyl[C](6);Carbamidomethyl[C](15)	4,25E-07	1,79E-02	6,817623
sp|P40424|PBX1_HUMAN (65)-sp|P55347-2|PKNX1_HUMAN (134)		7							
	CB_FB_180416_Prep1_Tryp_GluC_3.6041.6041.4.0.dta	4	1903,987187	KHALNCHR(1)-LEKVNE(3)	1903,980601	Carbamidomethyl[C](6)	8,86E-05	2,83E-04	3,459069
	CB_FB_180416_Prep1_Tryp_GluC_3.5852.5852.4.0.dta	4	1903,988696	KHALNCHR(1)-LEKVNE(3)	1903,980601	Carbamidomethyl[C](6)	1,42E-05	4,64E-04	4,251619
	CB_FB_180416_Prep1_Tryp_GluC_3.6247.6247.4.0.dta	4	1903,992024	KHALNCHR(1)-LEKVNE(3)	1903,980601	Carbamidomethyl[C](6)	2,53E-05	8,60E-04	5,999536
	CB_FB_180416_Prep1_Tryp_GluC_3.6148.6148.4.0.dta	4	1903,989296	KHALNCHR(1)-LEKVNE(3)	1903,980601	Carbamidomethyl[C](6)	7,15E-05	2,07E-03	4,566748
	CB_FB_180416_Prep1_Tryp_GluC_3.6454.6454.4.0.dta	4	1903,987391	KHALNCHR(1)-LEKVNE(3)	1903,980601	Carbamidomethyl[C](6)	8,19E-05	3,38E-03	3,566213
	CB_FB_180416_Prep1_Tryp_GluC_3.5854.5854.3.0.dta	3	1903,987774	KHALNCHR(1)-LEKVNE(3)	1903,980601	Carbamidomethyl[C](6)	5,41E-06	4,19E-03	3,76737
	CB_FB_180416_Prep1_Tryp_GluC_3.6859.6859.4.2.dta	4	1903,989531	KHALNCHR(1)-LEKVNE(3)	1903,980601	Carbamidomethyl[C](6)	1,98E-05	1,12E-02	4,690174
sp|P40424|PBX1_HUMAN (87)-sp|P55347-2|PKNX1_HUMAN (25)		1							
	CB_FB_180416_Prep1_Tryp_GluC_3.11184.11184.3.0.dta	3	1686,972671	KTVLSIR(1)-LKTEQD(2)	1686,963545	null	2,04E-05	7,15E-04	5,40972
sp|P40424|PBX1_HUMAN (74)-sp|P40424|PBX1_HUMAN (195)		1							
	CB_FB_180416_Prep1_Tryp_GluC_3.17071.17071.3.0.dta	3	2402,264905	MKPALFNVLCE(2)-TRPISPKE(7)	2402,245703	Carbamidomethyl[C](10);Oxidation[M](1)	5,00E-13	3,83E-03	7,993354
sp|P40424|PBX1_HUMAN (195)-sp|P55347-2|PKNX1_HUMAN (268)		1							
	CB_FB_180416_Prep1_Tryp_GluC_3.9166.9166.5.0.dta	5	2801,557108	TRPISPKEIER(7)-GVLPKHATNVMR(5)	2801,545307	Oxidation[M](11)	2,37E-08	1,81E-02	4,212318
sp|P40424|PBX1_HUMAN (153)-sp|P55347-2|PKNX1_HUMAN (151)		1							
	CB_FB_180416_Prep1_Tryp_GluC_3.11216.11216.4.0.dta	4	1949,137576	AKLSQIR(2)-YIACLKTK(6)	1949,125135	Carbamidomethyl[C](4)	6,73E-06	5,36E-02	6,382864
sp|P40424|PBX1_HUMAN (65)-sp|P55347-2|PKNX1_HUMAN (25)		1							
	CB_FB_180416_Prep1_Tryp_GluC_3.6023.6023.3.0.dta	3	2905,338727	KHALNCHR(1)-LKTEQDPNCSEPDAE(2)	2905,320197	Carbamidomethyl[C](9);Carbamidomethyl[C](24)	4,19E-17	2,90E-01	6,377954
sp|P40424|PBX1_HUMAN (153)-sp|P55347-2|PKNX1_HUMAN (153)		1							
	CB_FB_180416_Prep1_Tryp_GluC_3.7547.7547.3.0.dta	3	1677,891223	AKLSQIR(2)-TKMNSE(2)	1677,883916	Oxidation[M](13)	3,92E-04	3,55E-01	4,35489

Table legend

Protein	Accession number of the proteins involved in cross linking; the site position of the xl-Lys on the protein is reported in brackets
Title	File name .dat (and corresponding .tif) of the ms2 annotated spectrum (as reported in the repositoty)
Spectrum Number	Frequency of the ms2 spectra
Charge	Charge of the fragmented ion
Precursor Mass	Observed mass from the precursor ion (Da)
Peptide	Sequence of cross-linked peptide pair, the site position of the Lys involved in cross-linking is reported in brackets
Peptide Mass	Theoretical mass of cross-linked peptide (Da)
Modifications	Cys Carbamidomethyl or Met Oxidation
Evalue	
Score	pLink score
Precursor Mass Error	precursor mass error in ppm

Criteria applied for manual evaluation of spectra confidently identified = E-value < 0,001 (corresponding to FDR < 0,05); the majority of the ions of the spectrum are assigned and 4 consecutive b or y ions for each xl peptide pair observed

Supplementary Table S7: PREP1-PBX1 looped peptides Experiment #2

Protein	Title	Spectrum Number	Precursor Mass	Peptide	Peptide Mass	Modifications	Evalue	Score	Precursor Mass Error
			(Da)		(Da)			pLINK	(ppm)
		Charge							
sp|P55347-2|PKNX1_HUMAN (260)(262)		9							
	CB_FB_180416_Prep1_Tryp_GluC_3.10137.10137.2.0.dta	2	1835,968731	LSILHQDDGSSKNKR(12)(14)	1835,960913	null	4,06E-79	9,32E-17	4,258261
	CB_FB_180416_Prep1_Tryp_GluC_3.9933.9933.2.0.dta	2	1835,968955	LSILHQDDGSSKNKR(12)(14)	1835,960913	null	3,28E-43	8,73E-16	4,380268
	CB_FB_180416_Prep1_Tryp_GluC_3.9926.9926.2.0.dta	2	1835,968956	LSILHQDDGSSKNKR(12)(14)	1835,960913	null	2,62E-45	2,04E-14	4,380812
	CB_FB_180416_Prep1_Tryp_GluC_3.10070.10070.3.0.dta	3	1835,969641	LSILHQDDGSSKNKR(12)(14)	1835,960913	null	2,13E-32	4,98E-13	4,753914
	CB_FB_180416_Prep1_Tryp_GluC_3.10308.10308.2.1.dta	2	1835,966195	LSILHQDDGSSKNKR(12)(14)	1835,960913	null	1,15E-39	1,37E-12	2,876968
	CB_FB_180416_Prep1_Tryp_GluC_3.9827.9827.2.0.dta	2	1835,968925	LSILHQDDGSSKNKR(12)(14)	1835,960913	null	3,81E-22	1,35E-10	4,363927
	CB_FB_180416_Prep1_Tryp_GluC_3.10421.10421.2.1.dta	2	1835,972486	LSILHQDDGSSKNKR(12)(14)	1835,960913	null	2,17E-20	2,18E-06	6,303511
	CB_FB_180416_Prep1_Tryp_GluC_3.3614.3614.2.0.dta	2	1029,536995	DGSSKNKR(5)(7)	1029,532299	null	3,30E-11	2,04E-05	4,561294
	CB_FB_180416_Prep1_Tryp_GluC_3.2982.2982.2.0.dta	2	914,508004	GSSKNKR(4)(6)	914,505361	null	6,42E-08	4,85E-04	2,890087
sp|P55347-2|PKNX1_HUMAN (102)(105)		7							
	CB_FB_180416_Prep1_Tryp_GluC_3.16715.16715.2.0.dta	2	1620,733702	KEGKPFFCEDPE(1)(4)	1620,724967	Carbamidomethyl[C](8)	7,42E-13	9,39E-13	5,389563
	CB_FB_180416_Prep1_Tryp_GluC_3.17175.17175.2.0.dta	2	1620,735116	KEGKPFFCEDPE(1)(4)	1620,724967	Carbamidomethyl[C](8)	2,28E-12	3,77E-08	6,262012
	CB_FB_180416_Prep1_Tryp_GluC_3.17040.17040.2.0.dta	2	1620,7374	KEGKPFFCEDPE(1)(4)	1620,724967	Carbamidomethyl[C](8)	1,78E-16	5,57E-08	7,671258
	CB_FB_180416_Prep1_Tryp_GluC_3.16874.16874.2.0.dta	2	1620,737029	KEGKPFFCEDPE(1)(4)	1620,724967	Carbamidomethyl[C](8)	2,62E-14	1,37E-06	7,442348
	CB_FB_180416_Prep1_Tryp_GluC_3.16794.16794.2.0.dta	2	1620,733904	KEGKPFFCEDPE(1)(4)	1620,724967	Carbamidomethyl[C](8)	4,84E-16	3,36E-06	5,514199
	CB_FB_180416_Prep1_Tryp_GluC_3.17331.17331.2.1.dta	2	1620,73517	KEGKPFFCEDPE(1)(4)	1620,724967	Carbamidomethyl[C](8)	1,64E-13	3,83E-04	6,295331
	CB_FB_180416_Prep1_Tryp_GluC_3.17230.17230.2.2.dta	2	1620,734928	KEGKPFFCEDPE(1)(4)	1620,724967	Carbamidomethyl[C](8)	1,12E-15	3,98E-03	6,146015
sp|P40424|PBX1_HUMAN (292)(293)		1							
	CB_FB_180416_Prep1_Tryp_GluC_3.8063.8063.2.0.dta	2	988,587947	YKKNIGK(2)(3)	988,582546	null	4,04E-17	1,41E-11	5,463378
sp|P40424|PBX1_HUMAN (85)(87)		1							
	CB_FB_180416_Prep1_Tryp_GluC_3.14434.14434.2.0.dta	2	1324,826938	IKEKTVLSIR(2)(4)	1324,819797	null	1,10E-30	8,95E-10	5,390167

Table legend

Protein	Accession number of the proteins involved in cross linking; the site position of the xl-Lys on the protein is reported in brackets
Title	File name .dat (and corresponding .tif) of the ms2 annotated spectrum (as reported in the repositoty)
Spectrum Number	Frequency of the ms2 spectra
Charge	Charge of the fragmented ion
Precursor Mass	Observed mass from the precursor ion (Da)
Peptide	Sequence of cross-linked peptide pair, the site position of the Lys involved in cross-linking is reported in brackets
Peptide Mass	Theoretical mass of cross-linked peptide (Da)
Modifications	Cys Carbamidomethyl or Met Oxidation
Evalue	
Score	pLink score
Precursor Mass Error	precursor mass error in ppm

Criteria applied for manual evaluation of spectra confidently identified = E-value < 0,001 (corresponding to FDR < 0,05); the majority of the ions of the spectrum are assigned and 4 consecutive b or y ions for each xl peptide pair observed

Supplementary Table S8: MEIS1-PBX1 cross-linked peptides Experiment #1

Protein	Title	Spectrum Number	Precursor Mass	Peptide	Peptide Mass	Modifications	Evalue	Score	Precursor Mass Error
			(Da)		(Da)			pLINK	(ppm)
		Charge							
sp|O00470|MEIS1_HUMAN (195)-sp|P40424|PBX1_HUMAN (153)		3							
	CB_FB_180212_Meis1_Tryp_GluC_1.10186.10186.3.0.dta	3	2204,156338	GGSKSDSEDITR(4)-AKLSQIR(2)	2204,151609	null	5,30E-10	2,95E-10	2,145497
	CB_FB_180212_Meis1_Tryp_GluC_1.10350.10350.3.0.dta	3	2333,20422	EGGSKSDSEDITR(5)-AKLSQIR(2)	2333,194197	null	7,48E-08	1,20E-09	4,295828
	CB_FB_180212_Meis1_Tryp_GluC_1.10404.10404.4.0.dta	4	2333,202239	EGGSKSDSEDITR(5)-AKLSQIR(2)	2333,194197	null	6,60E-10	1,11E-06	3,446777
sp|P40424|PBX1_HUMAN (153)-sp|P40424|PBX1_HUMAN (242)		2							
	CB_FB_180212_Meis1_Tryp_GluC_1.12142.12142.2.0.dta	2	1904,037031	AKLSQIR(2)-NFNKQATE(4)	1904,023512	null	4,64E-16	1,04E-08	7,100227
	CB_FB_180212_Meis1_Tryp_GluC_1.10613.10613.4.0.dta	4	2060,126643	AKLSQIR(2)-RNFNKQATE(5)	2060,124614	null	2,02E-06	2,45E-02	0,984892
sp|O00470|MEIS1_HUMAN (195)-sp|P40424|PBX1_HUMAN (242)		3							
	CB_FB_180212_Meis1_Tryp_GluC_1.10412.10412.3.0.dta	3	2469,137814	EGGSKSDSEDITR(5)-NFNKQATE(4)	2469,137469	null	3,56E-09	2,79E-05	0,139725
	CB_FB_180212_Meis1_Tryp_GluC_1.10433.10433.2.0.dta	2	2469,133872	EGGSKSDSEDITR(5)-NFNKQATE(4)	2469,137469	null	5,00E-04	1,24E-01	-1,456784
	CB_FB_180212_Meis1_Tryp_GluC_1.10274.10274.3.0.dta	3	2340,102758	GGSKSDSEDITR(4)-NFNKQATE(4)	2340,094881	null	2,42E-10	6,81E-05	3,366103
sp|O00470|MEIS1_HUMAN (178)-sp|P40424|PBX1_HUMAN (153)		1							
	CB_FB_180212_Meis1_Tryp_GluC_1.12464.12464.3.0.dta	3	1921,09885	YISCLKGK(6)-AKLSQIR(2)	1921,093837	Carbamidomethyl[C](4)	1,35E-19	4,97E-12	2,609451
sp|P40424|PBX1_HUMAN (153)-sp|P40424|PBX1_HUMAN (308)		1							
	CB_FB_180212_Meis1_Tryp_GluC_1.13196.13196.3.0.dta	3	1702,990327	AKLSQIR(2)-ANIYAAK(7)	1702,984949	null	1,94E-20	1,53E-11	3,157984
sp|O00470|MEIS1_HUMAN (161)-sp|P40424|PBX1_HUMAN (65)		2							
	CB_FB_180212_Meis1_Tryp_GluC_1.6521.6521.5.0.dta	5	1927,002383	LEKVHE(3)-KHALNCHR(1)	1926,996585	Carbamidomethyl[C](6)	1,19E-06	7,50E-09	3,008827
	CB_FB_180212_Meis1_Tryp_GluC_1.6538.6538.3.0.dta	3	1927,005073	LEKVHE(3)-KHALNCHR(1)	1926,996585	Carbamidomethyl[C](6)	7,21E-09	1,88E-08	4,404782
sp|O00470|MEIS1_HUMAN (195)-sp|P40424|PBX1_HUMAN (308)		1							
	CB_FB_180212_Meis1_Tryp_GluC_1.11496.11496.3.0.dta	3	2268,109023	EGGSKSDSEDITR(5)-ANIYAAK(7)	2268,098907	null	2,57E-16	1,27E-08	4,460123
sp|P40424|PBX1_HUMAN (297)-sp|P40424|PBX1_HUMAN (308)		1							
	CB_FB_180212_Meis1_Tryp_GluC_1.15874.15874.2.0.dta	2	1851,960175	NIGKFQEE(4)-ANIYAAK(7)	1851,948622	null	2,73E-06	1,15E-05	6,238294
sp|O00470|MEIS1_HUMAN (178)-sp|O00470|MEIS1_HUMAN (195)		1							
	CB_FB_180212_Meis1_Tryp_GluC_1.10734.10734.4.2.dta	4	2357,175313	YISCLKGK(6)-GGSKSDSEDITR(4)	2357,165206	Carbamidomethyl[C](19)	5,96E-07	8,33E-05	4,287778
sp|O00470|MEIS1_HUMAN (195)-sp|P40424|PBX1_HUMAN (297)		1							
	CB_FB_180212_Meis1_Tryp_GluC_1.12715.12715.3.0.dta	3	2482,166163	EGGSKSDSEDITR(5)-NIGKFQEE(4)	2482,15787	null	3,02E-07	3,53E-03	3,341045
sp|O00470|MEIS1_HUMAN (126)-sp|O00470|MEIS1_HUMAN (132)		1							
	CB_FB_180212_Meis1_Tryp_GluC_1.18959.18959.3.1.dta	3	2516,350802	DIAVFAKQIR(7)-AEKPLFSSNPE(3)	2516,3394	null	2,36E-08	2,21E-02	4,531185

Table legend

Protein	Accession number of the proteins involved in cross linking; the site position of the xl-Lys on the protein is reported in brackets
Title	File name .dat (and corresponding .tif) of the ms2 annotated spectrum (as reported in the repositoty)
Spectrum Number	Frequency of the ms2 spectra
Charge	Charge of the fragmented ion
Precursor Mass	Observed mass from the precursor ion (Da)
Peptide	Sequence of cross-linked peptide pair, the site position of the Lys involved in cross-linking is reported in brackets
Peptide Mass	Theoretical mass of cross-linked peptide (Da)
Modifications	Cys Carbamidomethyl or Met Oxidation
Evalue	
Score	pLink score
Precursor Mass Error	precursor mass error in ppm

Criteria applied for manual evaluation of spectra confidently identified = E-value < 0,001 (corresponding to FDR < 0,05); the majority of the ions of the spectrum are assigned and 4 consecutive b or y ions for each xl peptide pair observed

Supplementary Table S9: MEIS1-PBX1 looped peptides Experiment #1

Protein	Title	Spectrum Number	Precursor Mass	Peptide	Peptide Mass	Modifications	Evalue	Score	Precursor Mass Error
			(Da)		(Da)			pLINK	(ppm)
		Charge							
sp|P40424|PBX1_HUMAN (292)(293)		10							
	CB_FB_180212_Meis1_Tryp_GluC_1.14086.14086.2.0.dta	2	1521,799292	YKKNIGKFQEE(2)(3)	1521,794702	null	1,55E-41	7,83E-16	3,016176
	CB_FB_180212_Meis1_Tryp_GluC_1.9800.9800.2.0.dta	2	988,584694	YKKNIGK(2)(3)	988,582546	null	3,44E-15	2,18E-13	2,172808
	CB_FB_180212_Meis1_Tryp_GluC_1.9708.9708.2.0.dta	2	988,584559	YKKNIGK(2)(3)	988,582546	null	1,66E-19	5,37E-13	2,036249
	CB_FB_180212_Meis1_Tryp_GluC_1.9896.9896.2.0.dta	2	988,585122	YKKNIGK(2)(3)	988,582546	null	1,39E-15	2,00E-12	2,605751
	CB_FB_180212_Meis1_Tryp_GluC_1.9931.9931.2.1.dta	2	988,586181	YKKNIGK(2)(3)	988,582546	null	4,90E-17	7,44E-11	3,676982
	CB_FB_180212_Meis1_Tryp_GluC_1.14223.14223.2.0.dta	2	1392,762496	YKKNIGKFQE(2)(3)	1392,752114	null	3,74E-35	1,91E-11	7,454306
	CB_FB_180212_Meis1_Tryp_GluC_1.10317.10317.2.0.dta	2	1257,771428	IRYKKNIGK(4)(5)	1257,767706	null	1,18E-19	5,78E-11	2,959211
	CB_FB_180212_Meis1_Tryp_GluC_1.10345.10345.3.0.dta	3	1257,770542	IRYKKNIGK(4)(5)	1257,767706	null	1,50E-16	1,52E-09	2,254788
	CB_FB_180212_Meis1_Tryp_GluC_1.13716.13716.2.0.dta	2	1790,984771	IRYKKNIGKFQEE(4)(5)	1790,979862	null	8,64E-42	1,24E-09	2,740958
	CB_FB_180212_Meis1_Tryp_GluC_1.13704.13704.3.0.dta	3	1790,984642	IRYKKNIGKFQEE(4)(5)	1790,979862	null	1,55E-46	1,54E-08	2,66893
sp|P40424|PBX1_HUMAN (293)(297)		2							
	CB_FB_180212_Meis1_Tryp_GluC_1.14784.14784.2.0.dta	2	1521,799196	YKKNIGKFQEE(3)(7)	1521,794702	null	1,37E-11	2,43E-06	2,953092
	CB_FB_180212_Meis1_Tryp_GluC_1.15713.15713.2.0.dta	2	1230,642114	KNIGKFQEE(1)(5)	1230,636423	null	3,46E-09	9,67E-05	4,624437
sp|O00470|MEIS1_HUMAN (270)(271)		5							
	CB_FB_180212_Meis1_Tryp_GluC_1.3713.3713.2.0.dta	2	1024,580156	PDKDKKR(5)(6)	1024,578521	null	1,29E-22	2,73E-14	1,595778
	CB_FB_180212_Meis1_Tryp_GluC_1.3695.3695.3.0.dta	3	1024,579677	PDKDKKR(5)(6)	1024,578521	null	1,88E-15	4,06E-14	1,128269
	CB_FB_180212_Meis1_Tryp_GluC_1.3874.3874.3.0.dta	3	1024,578817	PDKDKKR(5)(6)	1024,578521	null	2,04E-16	2,60E-12	0,288899
	CB_FB_180212_Meis1_Tryp_GluC_1.4435.4435.3.1.dta	3	1024,579647	PDKDKKR(5)(6)	1024,578521	null	5,08E-13	1,74E-09	1,098988
	CB_FB_180212_Meis1_Tryp_GluC_1.4439.4439.2.0.dta	2	1024,580785	PDKDKKR(5)(6)	1024,578521	null	2,11E-15	7,60E-09	2,209689

Table legend

Protein	Accession number of the proteins involved in cross linking; the site position of the xl-Lys on the protein is reported in brackets
Title	File name .dat (and corresponding .tif) of the ms2 annotated spectrum (as reported in the repositoty)
Spectrum Number	Frequency of the ms2 spectra
Charge	Charge of the fragmented ion
Precursor Mass	Observed mass from the precursor ion (Da)
Peptide	Sequence of cross-linked peptide pair, the site position of the Lys involved in cross-linking is reported in brackets
Peptide Mass	Theoretical mass of cross-linked peptide (Da)
Modifications	Cys Carbamidomethyl or Met Oxidation
Evalue	
Score	pLink score
Precursor Mass Error	precursor mass error in ppm

Criteria applied for manual evaluation of spectra confidently identified = E-value < 0,001 (corresponding to FDR < 0,05); the majority of the ions of the spectrum are assigned and 4 consecutive b or y ions for each xl peptide pair observed

Supplementary Table S10: MEIS1-PBX1 cross-linked peptides Experiment #2

Protein	Title	Spectrum Number	Precursor Mass	Peptide	Peptide Mass	Modifications	Evalue	Score	Precursor Mass Error
			(Da)		(Da)			pLINK	(ppm)
		Charge							
sp|O00470|MEIS1_HUMAN (195)-sp|P40424|PBX1_HUMAN (153)		8							
	CB_FB_180416_Meis1_Tryp_GluC_7.8044.8044.3.0.dta	3	2333,204641	EGGSKSDSEDITR(5)-AKLSQIR(2)	2333,194197	null	9,12E-14	2,54E-17	4,476267
	CB_FB_180416_Meis1_Tryp_GluC_7.8131.8131.4.0.dta	4	2333,204031	EGGSKSDSEDITR(5)-AKLSQIR(2)	2333,194197	null	4,01E-12	8,35E-15	4,214823
	CB_FB_180416_Meis1_Tryp_GluC_7.8034.8034.4.0.dta	4	2333,203856	EGGSKSDSEDITR(5)-AKLSQIR(2)	2333,194197	null	1,24E-14	7,58E-14	4,139818
	CB_FB_180416_Meis1_Tryp_GluC_7.8024.8024.4.0.dta	4	2333,203901	EGGSKSDSEDITR(5)-AKLSQIR(2)	2333,194197	null	5,45E-12	1,98E-06	4,159105
	CB_FB_180416_Meis1_Tryp_GluC_7.7868.7868.4.0.dta	4	2204,163668	GGSKSDSEDITR(4)-AKLSQIR(2)	2204,151609	null	6,80E-09	6,21E-15	5,471039
	CB_FB_180416_Meis1_Tryp_GluC_7.7933.7933.4.1.dta	4	2204,163795	GGSKSDSEDITR(4)-AKLSQIR(2)	2204,151609	null	1,90E-03	3,54E-01	5,528658
	CB_FB_180416_Meis1_Tryp_GluC_7.7342.7342.3.0.dta	3	1847,941575	EGGSKSDSE(5)-AKLSQIR(2)	1847,934425	null	5,00E-03	3,44E-05	3,869185
	CB_FB_180416_Meis1_Tryp_GluC_7.7158.7158.3.0.dta	3	1718,898544	GGSKSDSE(4)-AKLSQIR(2)	1718,891837	null	9,49E-04	3,64E-05	3,901933
sp|O00470|MEIS1_HUMAN (195)-sp|P40424|PBX1_HUMAN (242)		4							
	CB_FB_180416_Meis1_Tryp_GluC_7.8109.8109.3.0.dta	3	2469,14994	EGGSKSDSEDITR(5)-NFNKQATE(4)	2469,137469	null	3,05E-07	8,12E-07	5,050752
	CB_FB_180416_Meis1_Tryp_GluC_7.7999.7999.3.0.dta	3	2340,107503	GGSKSDSEDITR(4)-NFNKQATE(4)	2340,094881	null	1,70E-04	4,90E-05	5,393798
	CB_FB_180416_Meis1_Tryp_GluC_7.6996.6996.3.0.dta	3	2625,258863	EGGSKSDSEDITR(5)-RNFNKQATE(5)	2625,238571	null	2,36E-06	3,20E-04	7,729583
	CB_FB_180416_Meis1_Tryp_GluC_7.6823.6823.4.0.dta	4	2496,208006	GGSKSDSEDITR(4)-RNFNKQATE(5)	2496,195983	null	1,01E-05	1,73E-03	4,816529
sp|P40424|PBX1_HUMAN (87)-sp|P40424|PBX1_HUMAN (195)		9							
	CB_FB_180416_Meis1_Tryp_GluC_7.10344.10344.5.0.dta	5	2279,355629	KTVLSIR(1)-TRPISPKEIER(7)	2279,344428	null	4,68E-14	6,69E-20	4,914132
	CB_FB_180416_Meis1_Tryp_GluC_7.10313.10313.4.0.dta	4	2279,35591	KTVLSIR(1)-TRPISPKEIER(7)	2279,344428	null	1,44E-10	3,01E-14	5,037413
	CB_FB_180416_Meis1_Tryp_GluC_7.10396.10396.4.0.dta	4	2279,356188	KTVLSIR(1)-TRPISPKEIER(7)	2279,344428	null	1,93E-08	1,14E-12	5,159378
	CB_FB_180416_Meis1_Tryp_GluC_7.10321.10321.3.0.dta	3	2279,356111	KTVLSIR(1)-TRPISPKEIER(7)	2279,344428	null	1,07E-13	4,81E-07	5,125597
	CB_FB_180416_Meis1_Tryp_GluC_7.9873.9873.3.0.dta	3	1881,127616	KTVLSIR(1)-TRPISPKE(7)	1881,116681	null	2,27E-17	3,10E-19	5,813037
	CB_FB_180416_Meis1_Tryp_GluC_7.9960.9960.3.0.dta	3	1881,12797	KTVLSIR(1)-TRPISPKE(7)	1881,116681	null	8,58E-10	7,88E-18	6,001223
	CB_FB_180416_Meis1_Tryp_GluC_7.9896.9896.4.0.dta	4	1881,127158	KTVLSIR(1)-TRPISPKE(7)	1881,116681	null	2,48E-09	3,55E-14	5,569564
	CB_FB_180416_Meis1_Tryp_GluC_7.9916.9916.4.0.dta	4	1881,127146	KTVLSIR(1)-TRPISPKE(7)	1881,116681	null	1,09E-04	6,54E-06	5,563185
	CB_FB_180416_Meis1_Tryp_GluC_7.11441.11441.3.0.dta	3	2123,256909	KTVLSIR(1)-TRPISPKEIE(7)	2123,243326	null	3,64E-14	2,81E-14	6,397288
sp|P40424|PBX1_HUMAN (242)-sp|P40424|PBX1_HUMAN (308)		3							
1	CB_FB_180416_Meis1_Tryp_GluC_7.10682.10682.2.0.dta	2	1838,942311	NFNKQATE(4)-ANIYAAK(7)	1838,928221	null	4,63E-09	4,20E-13	7,662072
5	CB_FB_180416_Meis1_Tryp_GluC_7.10994.10994.2.0.dta	2	1967,983086	NFNKQATE(4)-EANIYAAK(8)	1967,970809	null	7,38E-09	1,31E-12	6,238406
6	CB_FB_180416_Meis1_Tryp_GluC_7.9248.9248.2.0.dta	2	1995,036839	RNFNKQATE(5)-ANIYAAK(7)	1995,029323	null	3,88E-10	7,07E-10	3,767363
sp|O00470|MEIS1_HUMAN (195)-sp|P40424|PBX1_HUMAN (308)		2							
1	CB_FB_180416_Meis1_Tryp_GluC_7.8812.8812.3.0.dta	3	2139,067874	GGSKSDSEDITR(4)-ANIYAAK(7)	2139,056319	null	5,26E-12	3,28E-19	5,401915
2	CB_FB_180416_Meis1_Tryp_GluC_7.9005.9005.3.0.dta	3	2268,109765	EGGSKSDSEDITR(5)-ANIYAAK(7)	2268,098907	null	3,00E-10	2,53E-12	4,787269
sp|P40424|PBX1_HUMAN (153)-sp|P40424|PBX1_HUMAN (308)		3							
1	CB_FB_180416_Meis1_Tryp_GluC_7.10384.10384.3.0.dta	3	1702,996098	AKLSQIR(2)-ANIYAAK(7)	1702,984949	null	1,77E-18	1,07E-18	6,54674
2	CB_FB_180416_Meis1_Tryp_GluC_7.10395.10395.2.0.dta	2	1702,996694	AKLSQIR(2)-ANIYAAK(7)	1702,984949	null	5,50E-10	1,16E-16	6,896714
3	CB_FB_180416_Meis1_Tryp_GluC_7.10643.10643.3.2.dta	3	1832,038783	AKLSQIR(2)-EANIYAAK(8)	1832,027537	null	2,21E-16	2,76E-07	6,138554
sp|P40424|PBX1_HUMAN (153)-sp|P40424|PBX1_HUMAN (242)		5							
1	CB_FB_180416_Meis1_Tryp_GluC_7.9535.9535.2.0.dta	2	1904,036346	AKLSQIR(2)-NFNKQATE(4)	1904,023512	null	2,41E-09	4,92E-14	6,740463
2	CB_FB_180416_Meis1_Tryp_GluC_7.9520.9520.3.0.dta	3	1904,035873	AKLSQIR(2)-NFNKQATE(4)	1904,023512	null	6,43E-09	1,47E-10	6,492042
4	CB_FB_180416_Meis1_Tryp_GluC_7.8182.8182.3.0.dta	3	2060,136096	AKLSQIR(2)-RNFNKQATE(5)	2060,124614	null	3,43E-08	2,50E-10	5,573449
5	CB_FB_180416_Meis1_Tryp_GluC_7.8167.8167.3.0.dta	3	2060,136096	AKLSQIR(2)-RNFNKQATE(5)	2060,124614	null	3,23E-04	4,26E-09	5,573449
6	CB_FB_180416_Meis1_Tryp_GluC_7.8190.8190.4.0.dta	4	2060,136032	AKLSQIR(2)-RNFNKQATE(5)	2060,124614	null	5,39E-05	1,00E-08	5,542383
sp|O00470|MEIS1_HUMAN (195)-sp|P40424|PBX1_HUMAN (87)		2							
1	CB_FB_180416_Meis1_Tryp_GluC_7.9528.9528.4.0.dta	4	2334,228502	EGGSKSDSEDITR(5)-KTVLSIR(1)	2334,214597	null	3,20E-12	2,44E-12	5,957036
2	CB_FB_180416_Meis1_Tryp_GluC_7.8044.8044.3.1.dta	3	2334,207154	GGSKSDSEDITR(4)-EKTVLSIR(2)	2334,214597	null	6,23E-02	6,74E-02	-3,188653
sp|P40424|PBX1_HUMAN (195)-sp|P40424|PBX1_HUMAN (308)		4							
1	CB_FB_180416_Meis1_Tryp_GluC_7.9408.9408.3.0.dta	3	1815,0174	TRPISPKE(7)-ANIYAAK(7)	1815,00099	null	2,20E-12	2,29E-09	9,041317
2	CB_FB_180416_Meis1_Tryp_GluC_7.9432.9432.2.0.dta	2	1815,015918	TRPISPKE(7)-ANIYAAK(7)	1815,00099	null	3,23E-08	1,43E-08	8,224789
3	CB_FB_180416_Meis1_Tryp_GluC_7.9874.9874.4.1.dta	4	2213,238917	TRPISPKEIER(7)-ANIYAAK(7)	2213,228738	null	1,26E-06	4,44E-07	4,599163
4	CB_FB_180416_Meis1_Tryp_GluC_7.9890.9890.3.0.dta	3	2213,241247	TRPISPKEIER(7)-ANIYAAK(7)	2213,228738	null	9,52E-11	1,96E-04	5,651924
sp|O00470|MEIS1_HUMAN (126)-sp|O00470|MEIS1_HUMAN (132)		1							
2	CB_FB_180416_Meis1_Tryp_GluC_7.15195.15195.3.0.dta	3	2516,346402	DIAVFAKQIR(7)-AEKPLFSSNPE(3)	2516,3394	null	1,23E-05	1,63E-03	2,782614
sp|P40424|PBX1_HUMAN (87)-sp|P40424|PBX1_HUMAN (153)		2							
1	CB_FB_180416_Meis1_Tryp_GluC_7.10616.10616.4.0.dta	4	1769,111238	KTVLSIR(1)-AKLSQIR(2)	1769,10064	null	9,51E-09	2,66E-18	5,990615
2	CB_FB_180416_Meis1_Tryp_GluC_7.10622.10622.3.0.dta	3	1769,112603	KTVLSIR(1)-AKLSQIR(2)	1769,10064	null	5,04E-09	4,61E-18	6,762193
sp|P40424|PBX1_HUMAN (87)-sp|P40424|PBX1_HUMAN (242)		2							
1	CB_FB_180416_Meis1_Tryp_GluC_7.11056.11056.3.0.dta	3	1905,056021	KTVLSIR(1)-NFNKQATE(4)	1905,043912	null	1,78E-08	2,06E-14	6,356284
2	CB_FB_180416_Meis1_Tryp_GluC_7.11069.11069.2.0.dta	2	1905,05776	KTVLSIR(1)-NFNKQATE(4)	1905,043912	null	2,00E-07	2,54E-14	7,269124
sp|O00470|MEIS1_HUMAN (161)-sp|P40424|PBX1_HUMAN (308)		1							
1	CB_FB_180416_Meis1_Tryp_GluC_7.9617.9617.3.0.dta	3	1641,89188	LEKVHE(3)-ANIYAAK(7)	1641,884573	null	1,74E-14	7,81E-12	4,450374
sp|O00470|MEIS1_HUMAN (161)-sp|O00470|MEIS1_HUMAN (195)		1							
1	CB_FB_180416_Meis1_Tryp_GluC_7.7254.7254.4.1.dta	4	2272,10574	LEKVHE(3)-EGGSKSDSEDITR(5)	2272,093821	null	5,50E-05	3,74E-09	5,245822

Table legend

Protein	Accession number of the proteins involved in cross linking; the site position of the xl-Lys on the protein is reported in brackets
Title	File name .dat (and corresponding .tif) of the ms2 annotated spectrum (as reported in the repositoty)
Spectrum Number	Frequency of the ms2 spectra
Charge	Charge of the fragmented ion
Precursor Mass	Observed mass from the precursor ion (Da)
Peptide	Sequence of cross-linked peptide pair, the site position of the Lys involved in cross-linking is reported in brackets
Peptide Mass	Theoretical mass of cross-linked peptide (Da)
Modifications	Cys Carbamidomethyl or Met Oxidation
Evalue	
Score	pLink score
Precursor Mass Error	precursor mass error in ppm

Criteria applied for manual evaluation of spectra confidently identified = E-value < 0,001 (corresponding to FDR < 0,05); the majority of the ions of the spectrum are assigned and 4 consecutive b or y ions for each xl peptide pair observed

Supplementary Table S11: MEIS1-PBX1 looped peptides Experiment #2

Protein	Title	Spectrum Number	Precursor Mass	Peptide	Peptide Mass	Modifications	Evalue	Score	Precursor Mass Error
			(Da)		(Da)			pLINK	(ppm)
		Charge							
sp|P40424|PBX1_HUMAN (85)(87)		1							
	CB_FB_180416_Meis1_Tryp_GluC_7.12585.12585.2.0.dta	2	1324,829242	IKEKTVLSIR(2)(4)	1324,819797	null	3,42E-11	1,27E-03	7,129271
sp|P40424|PBX1_HUMAN (292)(293)		1							
	CB_FB_180416_Meis1_Tryp_GluC_7.7278.7278.2.0.dta	2	988,587026	YKKNIGK(2)(3)	988,582546	null	1,24E-16	5,26E-03	4,531741
sp|O00470|MEIS1_HUMAN (305)(306)		1							
	CB_FB_180416_Meis1_Tryp_GluC_7.8586.8586.2.1.dta	2	1096,607915	QKKQLAQD(2)(3)	1096,599648	null	5,46E-07	4,05E-02	7,538759

Table legend

Protein	Accession number of the proteins involved in cross linking; the site position of the xl-Lys on the protein is reported in brackets
Title	File name .dat (and corresponding .tif) of the ms2 annotated spectrum (as reported in the repositoty)
Spectrum Number	Frequency of the ms2 spectra
Charge	Charge of the fragmented ion
Precursor Mass	Observed mass from the precursor ion (Da)
Peptide	Sequence of cross-linked peptide pair, the site position of the Lys involved in cross-linking is reported in brackets
Peptide Mass	Theoretical mass of cross-linked peptide (Da)
Modifications	Cys Carbamidomethyl or Met Oxidation
Evalue	
Score	pLink score
Precursor Mass Error	precursor mass error in ppm

Criteria applied for manual evaluation of spectra confidently identified = E-value < 0,001 (corresponding to FDR < 0,05); the majority of the ions of the spectrum are assigned and 4 consecutive b or y ions for each xl peptide pair observed

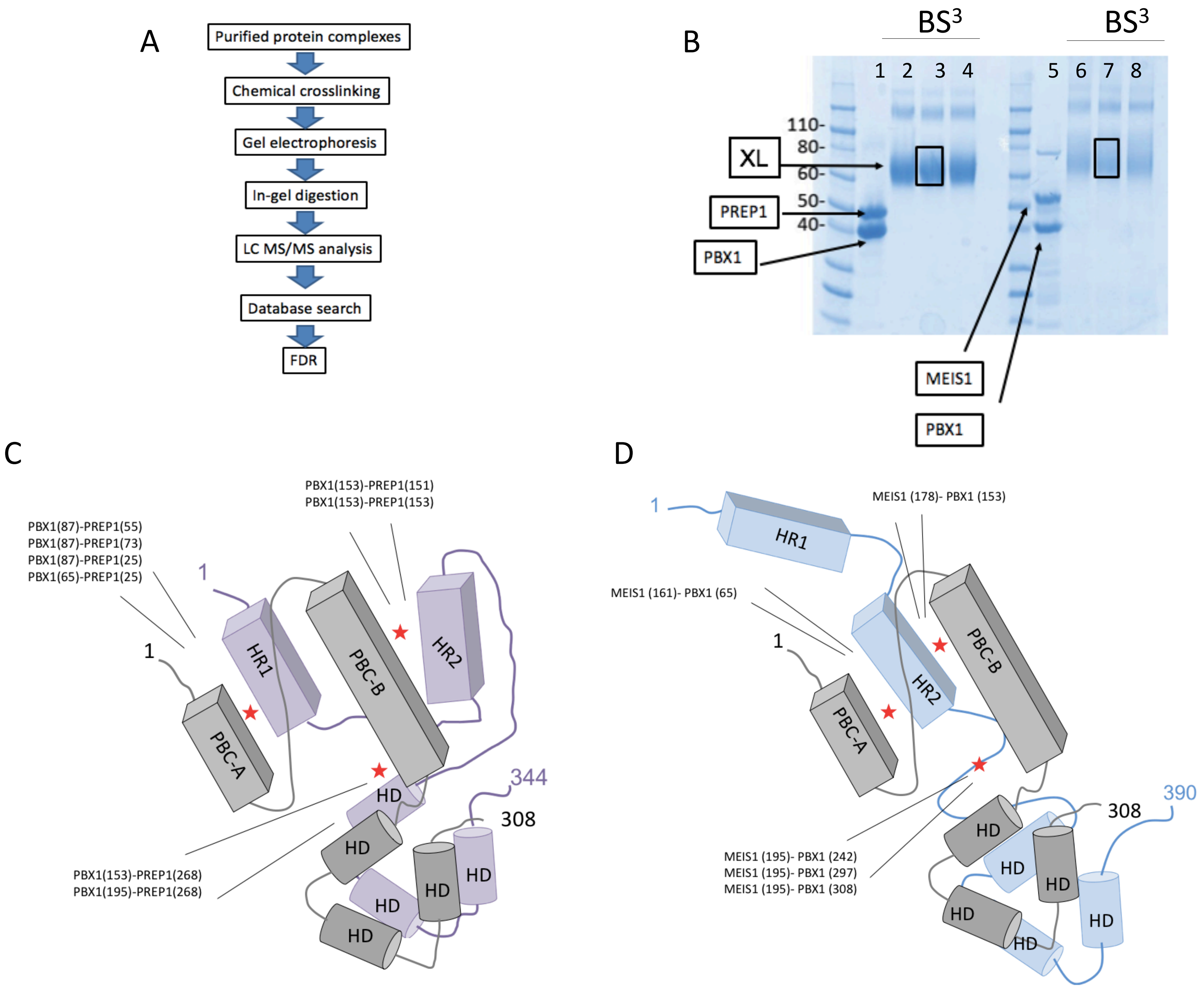

Supplementary S1

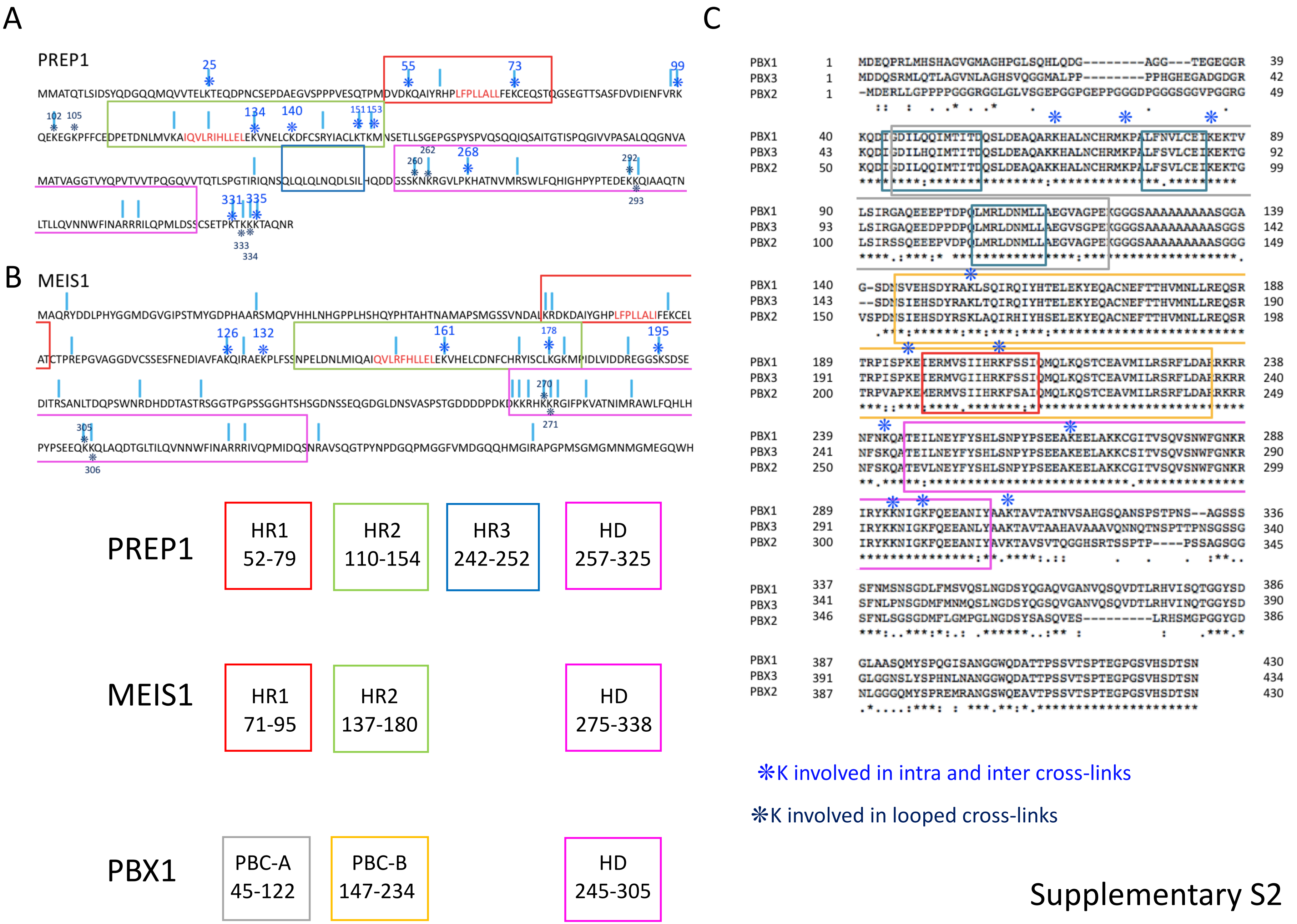

Supplementary S2

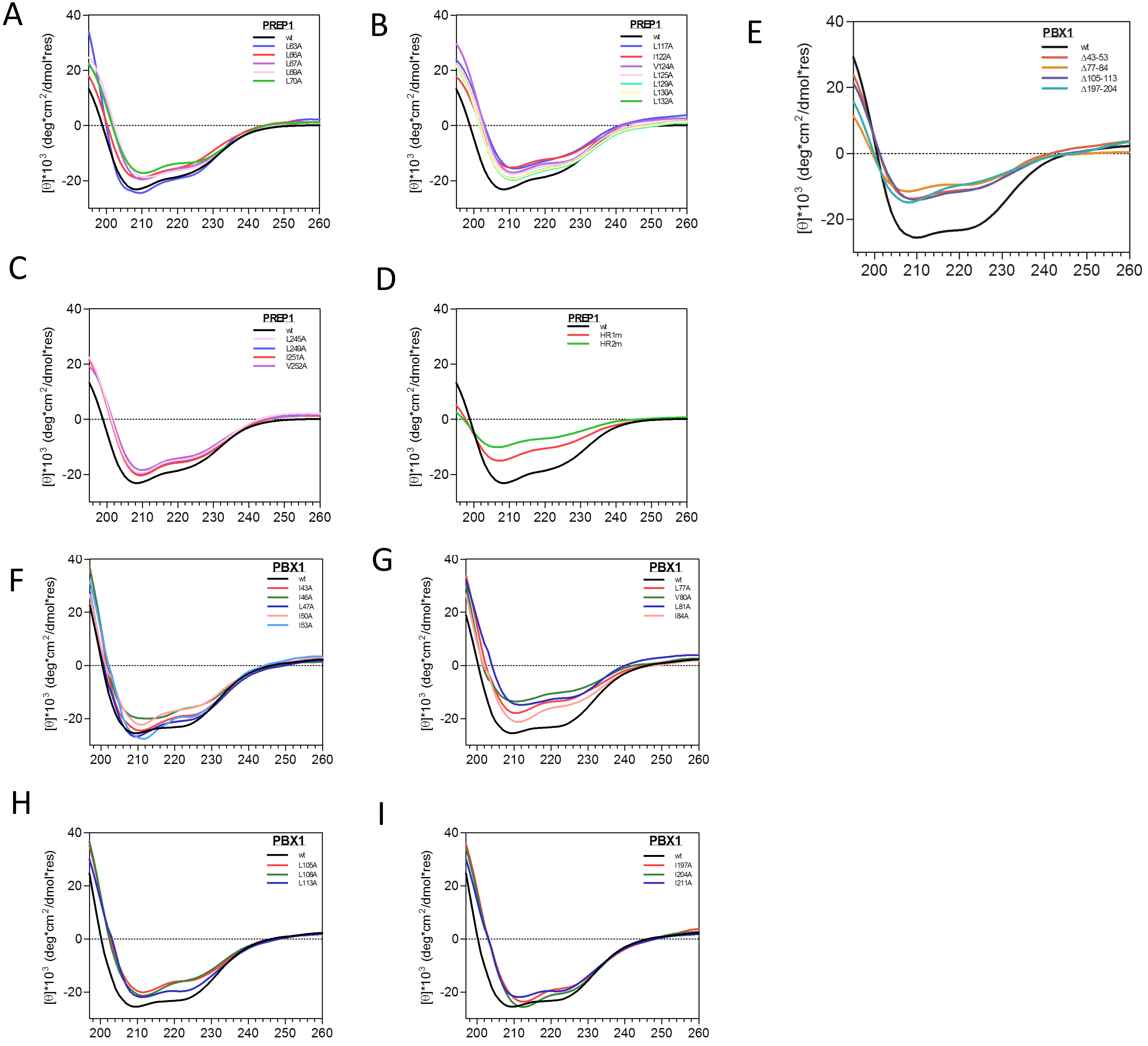

Supplementary S3

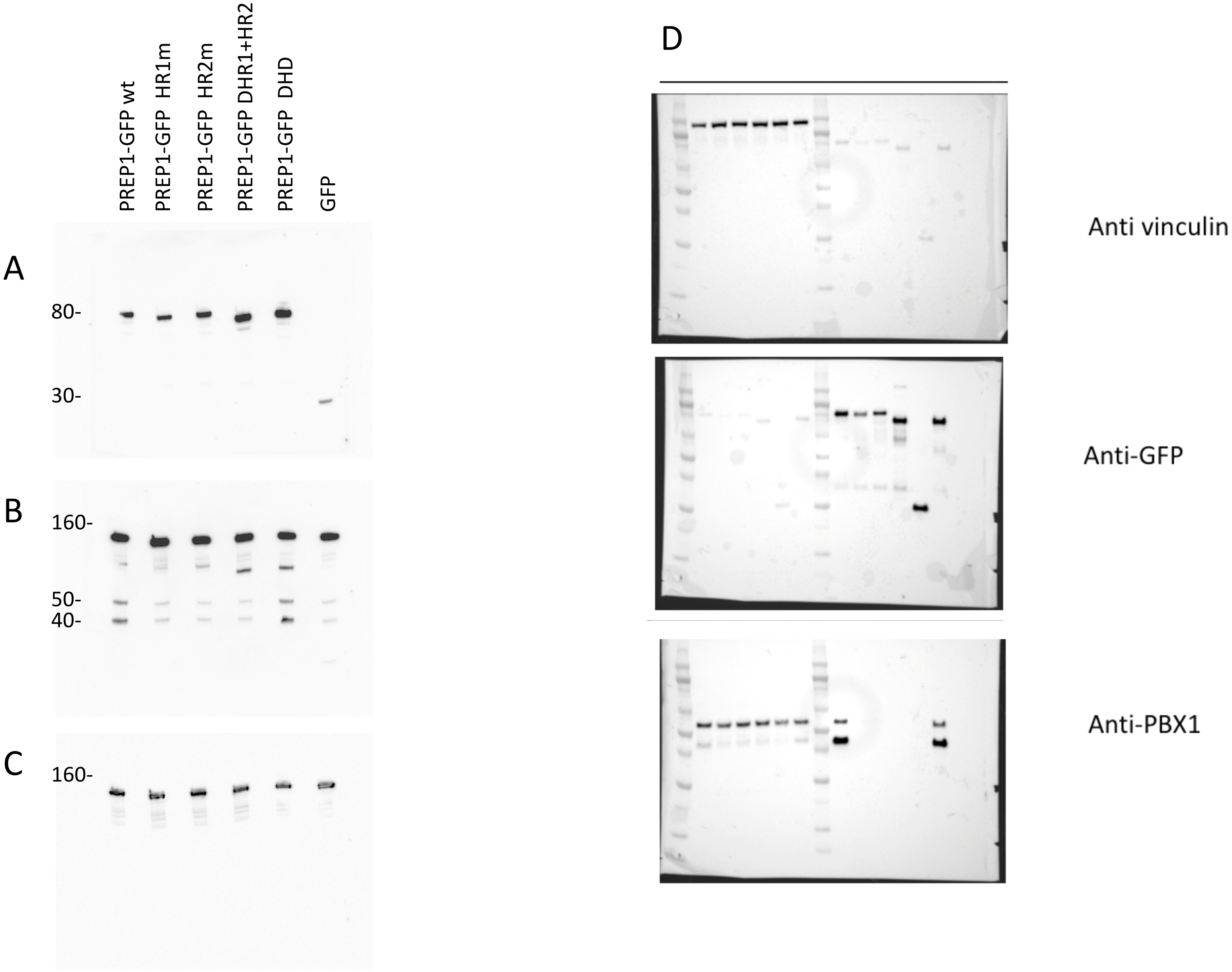

Supplementary S4

Supplementary S5

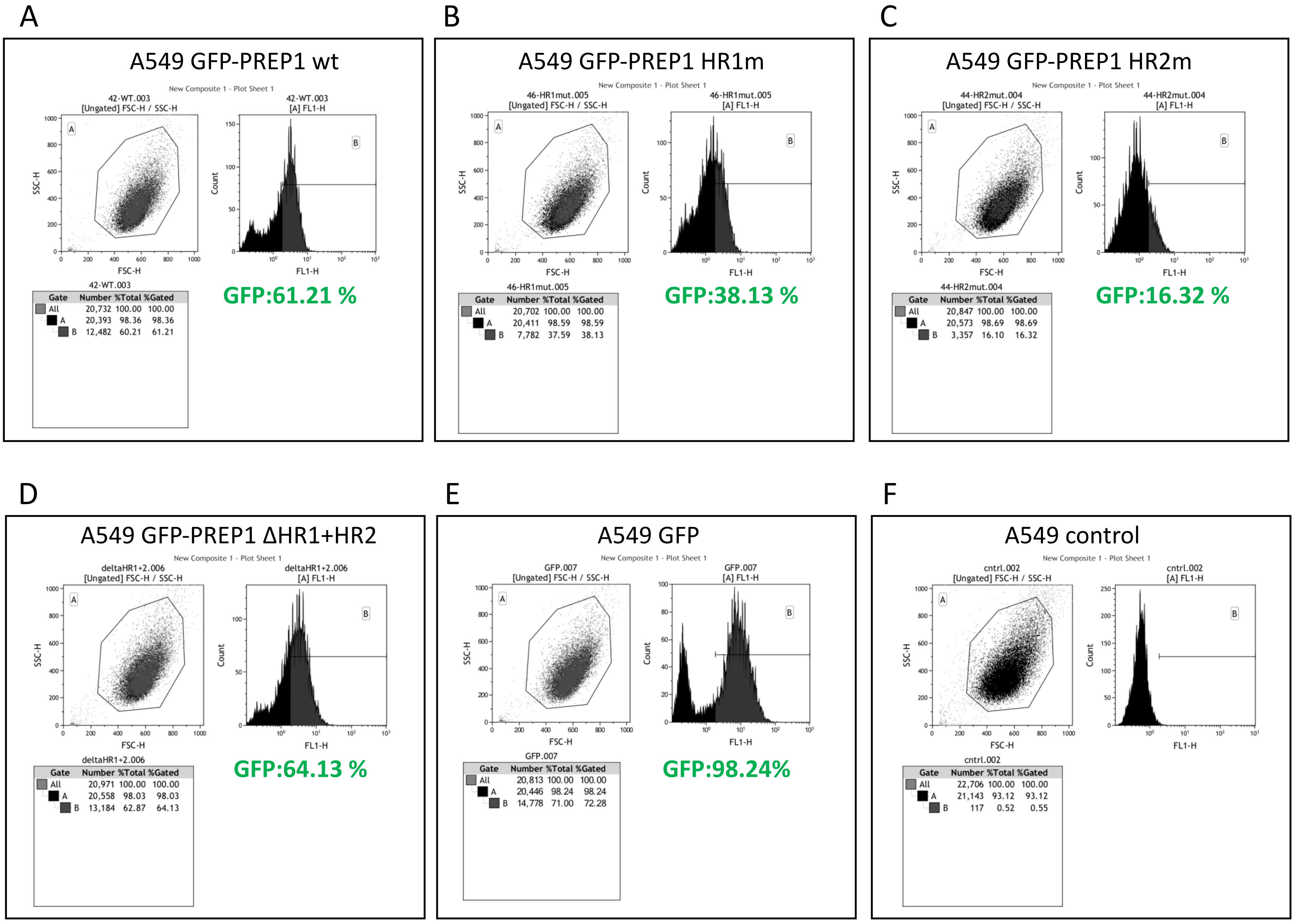

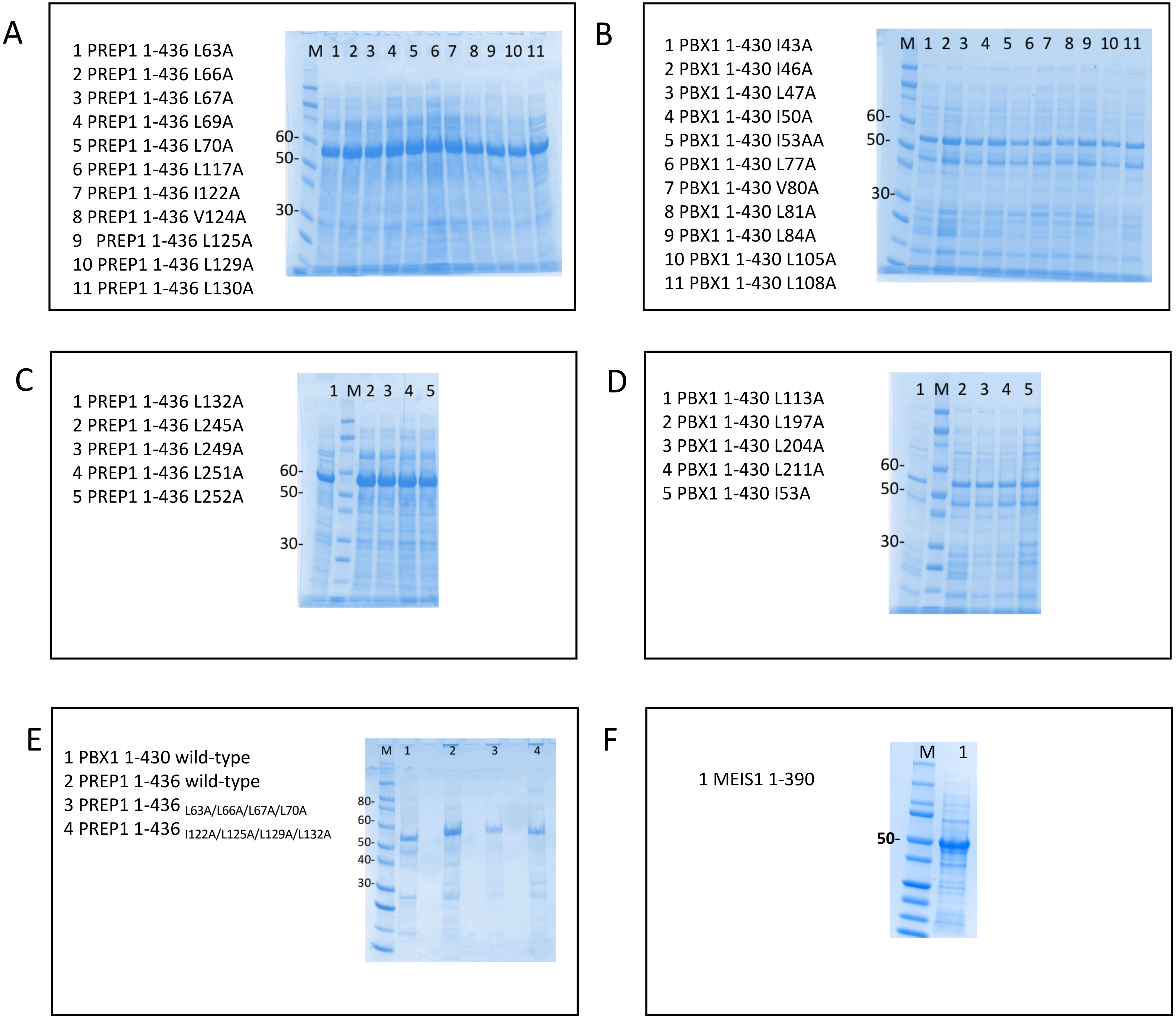

Supplementary S6
